# Multitargeted Aza-Arylcarboxamides for Neurodegenerative Diseases: Potent Histamine H_3_ Receptor Ligands with Anticholinesterase and Metal-Chelating Activities

**DOI:** 10.1021/acschemneuro.5c00803

**Published:** 2026-01-29

**Authors:** Flavia B. Lopes, Tobias Werner, Izilda A. Bagatin, Holger Stark, João Paulo S. Fernandes

**Affiliations:** † Department of Pharmaceutical Sciences, 28105 Federal University of São Paulo, Diadema 09913-030, Brazil; ‡ Department of Medicine, Federal University of São Paulo, São Paulo 04023-062, Brazil; § Institute of Pharmaceutical and Medicinal Chemistry, 9170 Heinrich Heine University Düsseldorf, Duesseldorf 40225, Germany; ∥ Department of Chemistry, Federal University of São Paulo, Diadema 09972-270, Brazil

**Keywords:** multitargeting, histamine H_3_ receptor, cholinesterase inhibitor, metal chelating compound, neurodegenerative disease

## Abstract

Neurodegenerative diseases are conditions characterized by neuronal loss in the nervous system, leading to diverse symptoms associated with complex pathological mechanisms. Dysregulation of metal ions such as iron and copper is linked to oxidative stress and consequently contributes to neuronal toxicity. Considering this, multitarget agents represent promising therapeutic strategies for the treatment of neurodegenerative disorders. In this study, a series of 24 novel multitarget compounds were designed to interact with histamine H_3_ receptors (H_3_R) and acetyl- and butyrylcholinesterases (AChE and BChE, respectively), incorporating additional metal-chelating groups. The compounds were synthesized and evaluated for their potency at H_3_R, for cholinesterase inhibitionand for metal-chelating activity toward Fe^2+^, Fe^3+^, and Cu^2+^ using spectrophotometric assays. The compounds displayed considerable affinities for H_3_R, AChE and BChE, with isoquinoline derivatives **LINS05413** and **LINS05414** standing out as multitarget agents due to their nanomolar affinities for H_3_R (p*K*_i_ = 6.41 and 6.37, respectively), moderate AChE inhibitory activities (pIC_50_ = 4.31 and 4.03, respectively) and metal-chelating properties. Isoquinoline-based compounds exhibited the strongest metal-chelating properties, particularly against copper, whereas 4-pyridylpiperazine derivatives were more effective in chelating iron ions. Molecular docking analyses revealed the role of aromatic substituents on multitargeting through interactions with key aromatic residues from each target. Structure–activity relationship and ligand efficiency analyses underscored the importance of the benzylpiperazine moiety for multitarget activity, while metal-chelating groups contributed to increased lipophilic ligand efficiency.

## Graphical abstract

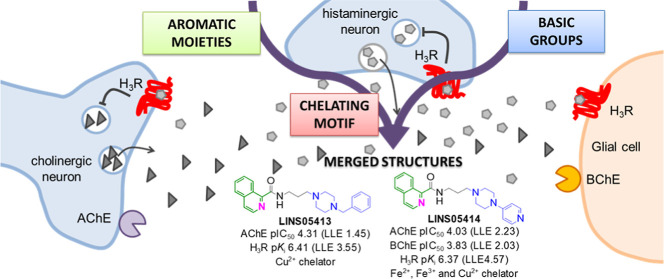

## Introduction

Neurodegeneration is a process characterized by a progressive and irreversible loss of neurons, predominantly affecting those within the central nervous system (CNS). Among the most prevalent neurodegenerative disorders are Alzheimer’s (AD) and Parkinson’s (PD) diseases, amyotrophic lateral sclerosis (ALS) and multiple sclerosis. The neurodegenerative process involves complex pathological mechanisms, with neuroinflammatory responses and oxidative stress being the most prominent contributors.^1^ The clinical symptoms of neurodegeneration vary depending on the localization and extension affected, but usually include motor, autonomic and cognitive alterations. For instance, AD and PD are tipically associated with cognitive and motor symptoms, respectively, but as the diseases progress, motor impairment and dementia occur in both.^2,3^ Therefore, therapeutic agents capable of preventing or slowing neurodegeneration (i.e., disease-modifying agents) while also alleviating clinical symptoms are highly desirable.

Restoring neurotransmitter activity associated with neuronal loss remains one of the main strategies to ameliorate the clinical symptoms of neurodegeneration, including cognitive decline. Histamine (HA) and acetylcholine (ACh) are key modulators of cognitive processes,^4,5^ and their cooperative roles were extensively demonstrated.^6,7^ The HA H_3_ receptor (H_3_R) is a presynaptic receptor expressed not only in histaminergic neurons but also as heteroreceptor on cholinergic and other neuronal populations, thereby regulating the synthesis and release of both HA and ACh (Figure 1). The pharmacological antagonism at H_3_R may increase the activity of both neurotransmitters, and indeed the cognitive-enhancing effects mediated by H_3_R blockers have been demonstrated in literature.^8^ Pitolisant, a selective H_3_R antagonist approved for the treatment of narcolepsy and obstructive sleep apnoea, has shown benefical effects on cognitive performance of children affected by Prader–Willi syndrome (PWS),^9^ and other H_3_R antagonists have also shown procognitive effects in (pre)­clinical models.^10^

**Figure 1:**
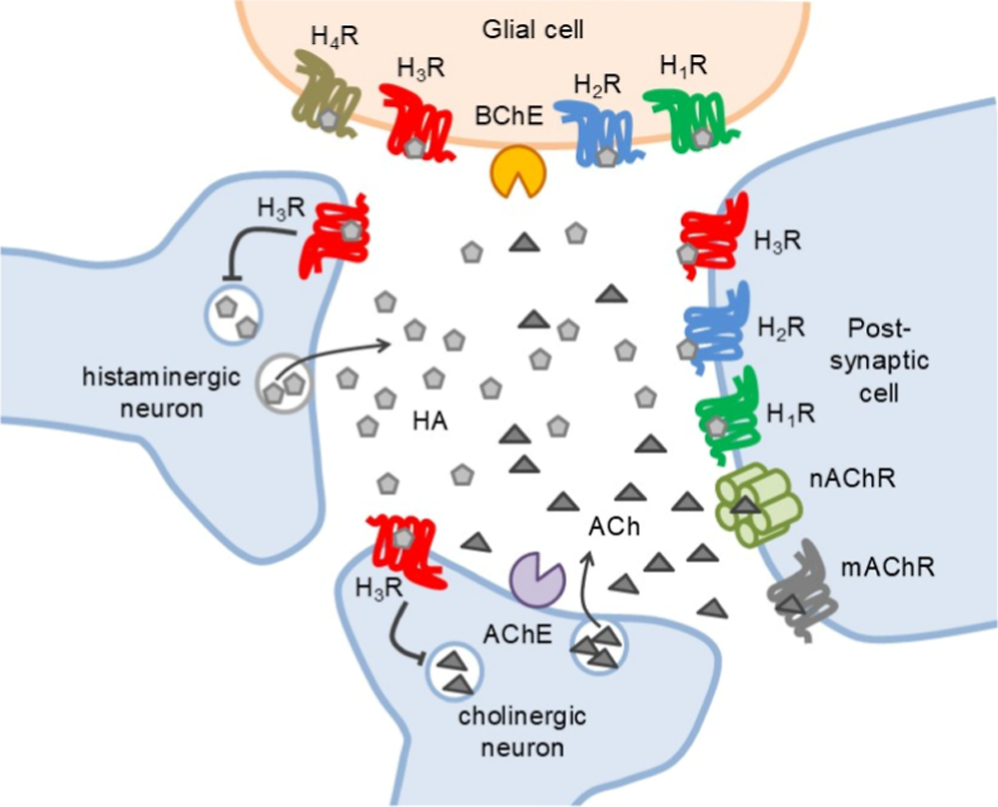
Schematic representation of a histaminergic-cholinergic synapse. Histamine (HA); acetylcholine (ACh); histamine receptor subtypes H_1_R to H_4_R (H_*x*_R); acetylcholinesterase (AChE); butyrylcholinesterase (BChE); nicotinic cholinergic receptor (nAChR); muscarinic cholinergic receptor (mAChR).

Since the primary mechanism terminating cholinergic transmission involves the hydrolysis by cholinesterases (ChEs), inhibition of acetyl (AChE) and/or butyrylcholinesterase (BChE) yields procognitive effects, which can be potentiated through H_3_R blockade.^7^ A clinical study reported significant cognitive improvement by coadministration of the inverse H_3_R agonist MK-3134 with the selective AChE inhibitor donepezil. The cognitive effect of this drug combination was higher than of either drug alone, highlighting the synergistic potential of dual H_3_R and ChEs inhibition.^11^ These findings support the design of dual H_3_R/ChEs inhibitors as promising agents against dementia and other cognitive disorders,^12,13^ and the potential of such agents was recently reviewed with several examples discussed therein.^7,14,15^ Moreover, accumulating evidence supports that dual AChE/BChE inhibitors (e.g., rivastigmine) promotes increased clinical benefits than selective AChE inhibitors (e.g., donepezil),^16^ suggesting that dual agents should be prioritized in drug discovery.

In addition, dysregulation of metal ion homeostasis can exacerbate neuronal oxidative stress and consequently increase neuronal toxicity. Iron and copper are particularly relevant, as their involvement in redox cycling promotes the formation of reactive oxygen species (ROS) through the Fenton reaction. ROS cause severe cellular damage and mitochondrial dysfunction, ultimately leading to neuronal death.^17^ Owing to its high metabolic rate, the brain is particularly susceptible to ROS-induced injury, and the link between neurodegeneration and iron^18^ and copper^19^ dyshomeostasis is well documented, especially in AD^20^ and PD.^21^ Moreover, iron is known to accumulate in amyloid plaques from AD patients, and may increase the BChE levels from glial cells.^22^ Consequently, brain metal-scavenging therapies have been explored as strategies against neurodegenerative diseases, including in clinical trials.^17,23^

In this work, we combined the procognitive effects of dual H_3_R/ChE ligands with metal-chelating functionality to provide enhanced neuroprotection against metal-induced toxicity. To achieve this, structural features from known H_3_R ligands, ChE inhibitors, and metal-chelating moieties were integrated into single molecular frameworks using a multitargeting strategy to combat neurodegenerative diseases.

## Results and Discussion

A set of 24 final compounds (**LINS05**xxx) was synthesized and evaluated. The compounds were designed using the general scaffold described in literature for dual H_3_R/ChEs ligands (Figure 2), comprising an aromatic lipophilic region and a substituted basic moiety connected through a variable linker.^6,7^ For **LINS05** series, the linkers consisted of either three (x**1**x) or five (x**3**x) methylene units, since linker length is known to influence both H_3_R and ChEs affinities. The aromatic substructures incorporated known metal-chelating motifs such as pyrazine (**2**xx), isoquinoline (**4**xx) and quinoline (**6**xx) carboxamides. As proof-of-concept, the corresponding benzene (**113**) and 1- and 2-naphthalene (**3**xx and **5**xx, respectively) carboxamides were also included. The basic substructures consisted of piperazines or piperidines substituted with propyl (xx**1**), allyl (xx**2**), benzyl (xx**3** and xx**6**) and 4-pyridyl (xx**4**) groups, selected based on previously reported H_3_R and ChEs ligands.^24–26^

**Figure 2:**
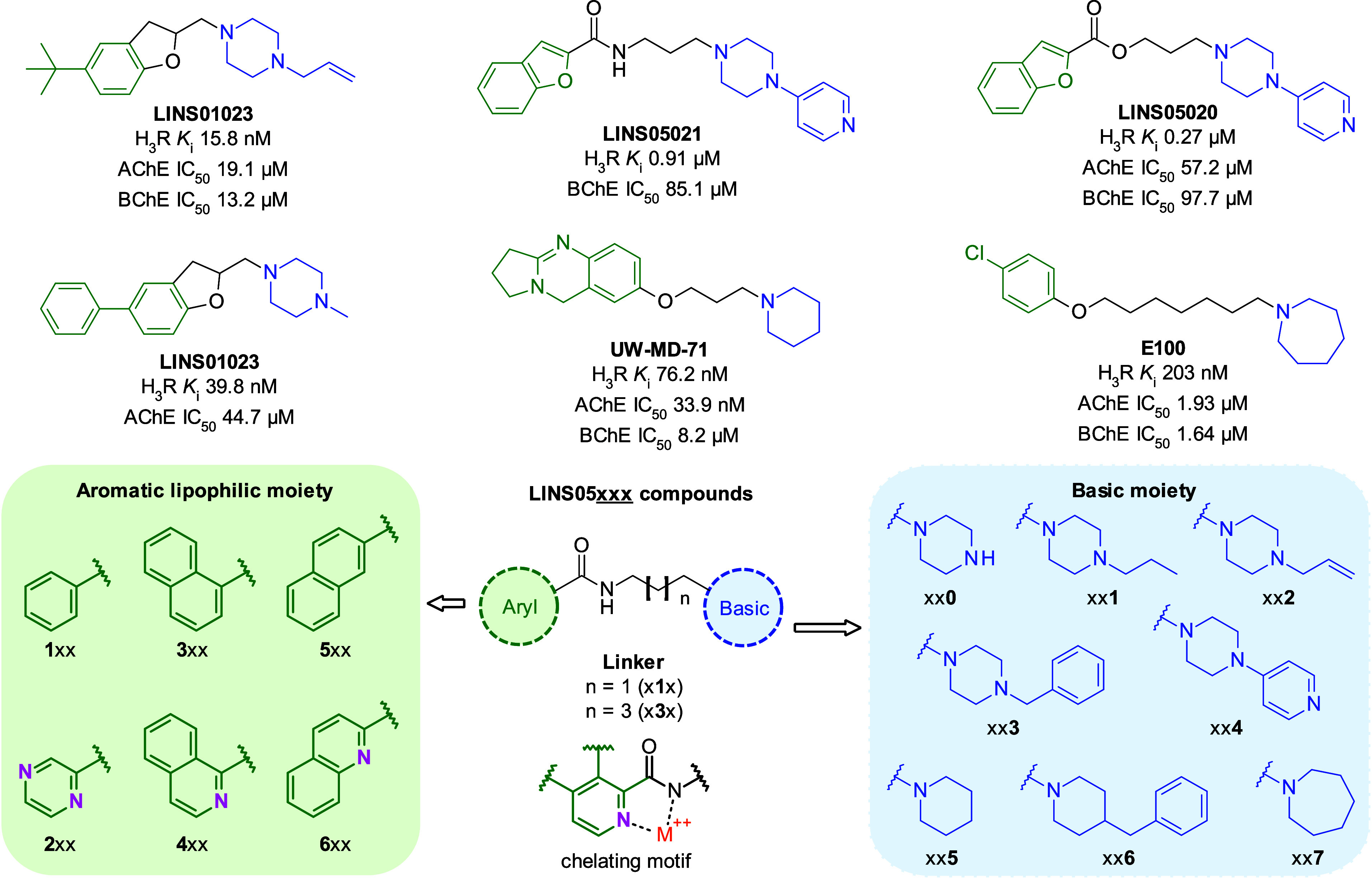
Conceptual design of the compounds **LINS05**. Green regions represent the aromatic lipophilic moiety and blue represent the basic substructures.

The compounds were synthesized from the corresponding carboxylic acid derivatives as depicted in the Scheme 1. For the amide formation, the appropriate acyl chlorides were directly reacted with corresponding amines (method B),^24,27^ affording the intermediates **1a–1d** in high yields. In the case of pyrazine-2-carboxamide **215**, pyrazinoic acid was converted in situ into the corresponding acyl chloride (method A) and subsequently reacted with the 1-(3-aminopropyl)­piperidine in a one-pot procedure previously described.^27,28^ For the (iso)­quinoline acids, the synthetic strategy was modified, as acyl chloride formation resulted in complex mixtures. Thus, intermediates **1e–1h** and the final compound **415** were obtained in moderate yields from the carboxylic acids using EDC as the coupling reagent and HOBt as a catalyst (method C).^27,29,30^

**Scheme 1:**
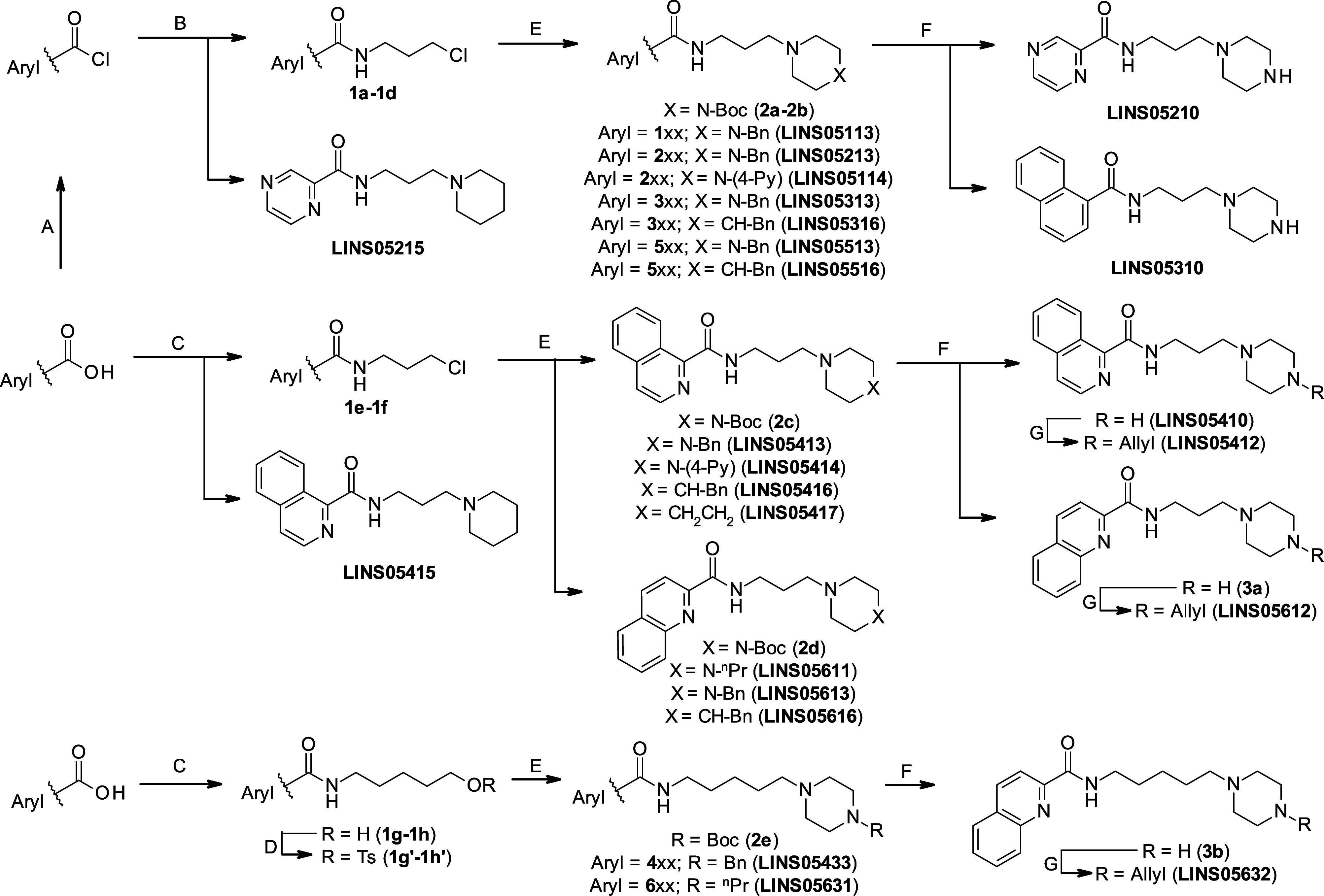
Reagents and Conditions: (A) Pyrazine-2-Carboxylic Acid, SOCl_2_ (1.5 Equiv), DCM, 50 °C, 3 h; (B) Amine (1.1 Equiv), TEA, DCM, r.t., 5–10 h; (C) EDC·HCl, HOBt·*x*H_2_O, DCM, 1–2 h Then Amine, 18–24 h; (D) TsCl, TEA, DCM, 50 °C, 12 h; (E) KI, K_2_CO_3_ (1.5 Equiv), Cycloamine (1.5 Equiv), AcN, 80 °C, 20–24 h; (F) TFA (3 Equiv), Water/DCM (1:12) r.t., 12–18 h; (G) Allyl Bromide, K_2_CO_3_ (2 Equiv), THF, r.t., 12–24 h

Alkyl halide intermediates **1a–1f** were directly reacted with substituted cycloamines (1-Boc-piperazine, 1-benzylpiperazine, 4-benzylpiperidine, 1-propylpiperazine, 1-(4-pyridyl)­piperazine or homopiperidine), to afford either the final compounds or Boc-protected intermediates **2a–2e** (method E).^24^ The hydroxylated intermediates **1g** and **1h** were converted into the corresponding tosylates^31^ **1g′** and **1h′** (method D), which were subsequently reacted with *N*-substituted piperazines (method E).

Boc-protected intermediates **2a–2e** were deprotected (method F) to obtain the corresponding piperazines **3a**-**3b** and final compounds **210**, **310** and **410**. The allylated derivatives (xx**2**) were prepared in good yields by alkylation of the corresponding piperazines with allyl bromide, following a previously reported procedure (method G).^26^ The final compounds were obtained with adequate purity (>95%) for pharmacological assays and were assessed for their inhibitory potency on AChE and BChE, as well as for their binding affinity at H_3_R.^24^ The final compounds were also evaluated for their metal-chelating activity toward Fe^2+^, Fe^3+^ and Cu^2+^ ions.^32,33^

The results (Table 1) highlight the influence of specific structural features on the observed activity profiles. Figure 3 provides a graphical representation of the **LINS05** compounds’ activity landscape through a structure activity relationship matrix (SARM).^34^

**Table 1:**
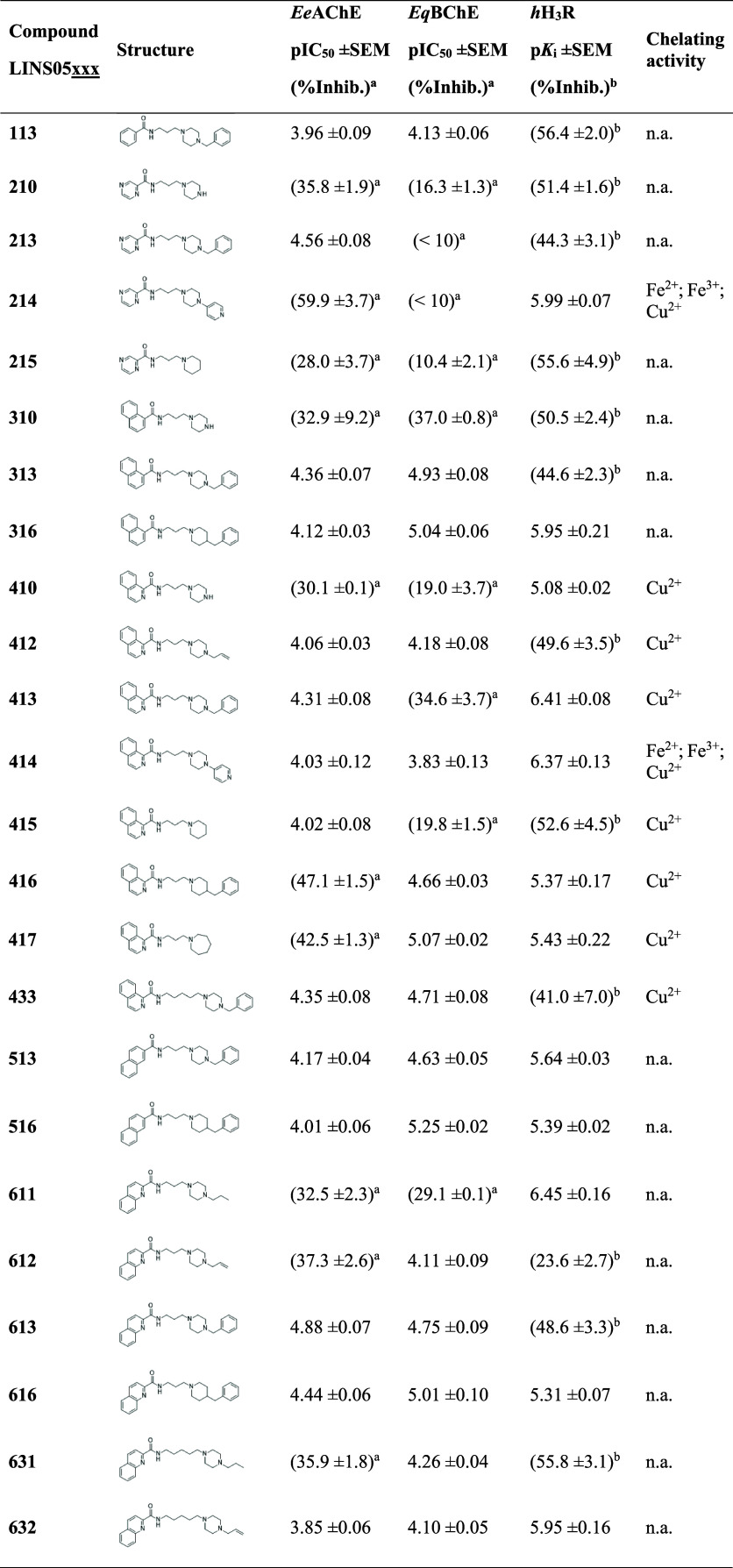
Results for the Pharmacological and Metal-Chelating Activity of the Compounds **LINS05**

a % inhibition at 100 μM.

b % inhibition ± SEM at 1 μM; n.a.: not active.

**Figure 3:**
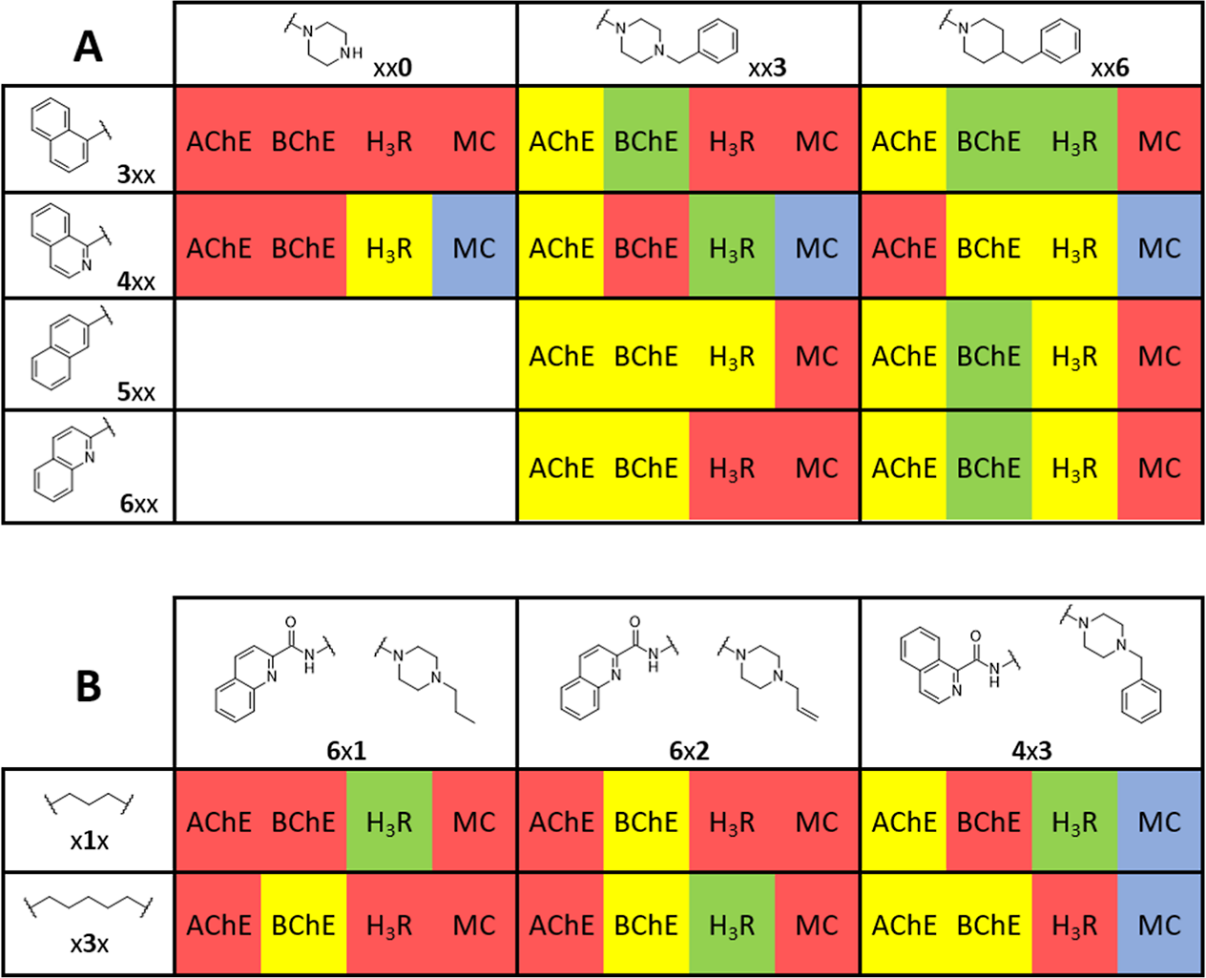
Structure activity relationship matrix (SARM) built with the results from compounds **LINS05**. (A) The rows represent the substructures in the aromatic lipophilic moiety and columns represent the substructures in the basic moiety. (B) Rows represent the substructures in the linker while columns represent the aromatic lipophilic (left) and basic (right) moieties. MC: metal chelating; red: not active (pIC_50_ < 4.0 and p*K*_i_ < 5.0); yellow: moderate activity; green: high activity (pIC_50_ > 5.0 and p*K*_i_ > 6.0); blue: metal chelating activity positive.

The logP and TPSA values clearly indicate that the heteroaromatic compounds (**2**xx, **4**xx and **6**xx) are less lipophilic than the phenyl or naphthyl derivatives. The correlation between these values and the blood–brain barrier (BBB) permeation is used to estimate their potential penetration into the CNS (Table 2). Except for the more hydrophilic pyrazinamides (**210**, **213** and **214**) and the unsubstituted piperazine **410**, all compounds were predicted to reach the CNS and exert the expected pharmacological effect in the brain.

**Table 2:** Calculated logP, BBB Permeation, Ligand Efficiency (LE) and Lipophilic Ligand Efficiency (LLE) Values of the **LINS05** Compounds for the Selected Targets

				LE			LLE		
compound **LINS05**xxx	logP	TPSA	BBB permeation	AChE	BChE	H_3_R	AChE	BChE	H_3_R
**113**	2.70	35.58	+	0.22	0.23	-	1.26	1.43	-
**210**	–1.46	70.15	-	-	-	-	-	-	-
**213**	0.65	61.36	-	0.26	-	-	3.91	-	-
**214**	–0.40	74.25	-	-	-	0.35	-	-	6.39
**215**	–0.07	58.12	+	-	-	-	-	-	-
**310**	1.58	44.37	+	-	-	-	-	-	-
**313**	3.69	32.34	+	0.21	0.24	-	0.67	1.24	-
**316**	4.83	35.58	+	0.20	0.24	0.29	–0.71	0.21	1.12
**410**	0.75	57.26	-	-	-	0.32	-	-	4.33
**412**	1.86	45.23	+	0.23	0.23	-	2.2	2.32	-
**413**	2.86	48.47	+	0.21	-	0.31	1.45	-	3.55
**414**	1.80	48.47	+	0.20	0.19	0.32	2.23	2.03	4.57
**415**	2.13	61.36	+	0.26	-	-	1.89	-	-
**416**	4.00	45.23	+	-	0.22	0.26	-	0.66	1.37
**417**	2.58	45.23	+	-	0.31	0.33	-	2.49	2.85
**433**	3.82	48.47	+	0.20	0.21	-	0.53	0.89	-
**513**	3.69	32.34	+	0.20	0.22	0.27	0.48	0.94	1.95
**516**	4.83	35.58	+	0.19	0.25	0.26	–0.82	0.42	0.56
**611**	2.40	45.23	+	-	-	0.36	-	-	4.05
**612**	2.25	48.47	+	-	0.23	-	-	1.86	-
**613**	3.24	48.47	+	0.24	0.23	-	1.64	1.51	-
**616**	4.39	48.47	+	0.21	0.24	0.26	0.05	0.62	0.92
**631**	3.36	48.47	+	-	0.22	-	-	0.9	-
**632**	3.21	48.47	+	0.20	0.21	0.31	0.64	0.89	2.74

With respect to H_3_R activity, compounds **214**, **316**, **413**, **414**, **611** and **632** displayed nanomolar affinities, while compounds **410**, **416**, **513**, **516** and **616** showed moderate affinities (p*K*_i_ 5.0–6.0) (Table 1 and Figure 3A). The benzylpiperazine **413**, propylpiperazine **611** and 4-pyridylpiperazine **414** displayed nanomolar affinities at H_3_R (p*K*_i_ 6.41, 6.45 and 6.37, respectively) ranking among the most potent compounds in the series. Other benzyl-containing compounds (e.g., **316**) also demonstrated improved affinities relative to their nonbenzylated analogues (Figure 3), suggesting that this group contributes to H_3_R binding through specific interactions. To the best of our knowledge, benzyl-substituted compounds exhibiting such considerable H_3_R affinity have been only rarely reported.

Regarding ChEs inhibition, the **LINS05** compounds displayed a general preference for BChE over AChE. The compounds **313**, **316**, **417**, **516** and **616** showed stronger inhibitory activity toward BChE, with compound **516** being the most potent (pIC_50_ 5.25). The 4-benzylpiperidines (xx**6**) were more potent and selective BChE inhibitors than their benzylpiperazine (xx**3**) counterparts (Figure 3A), reinforcing the role of this motif in modulating both potency and selectivity.

Earlier studies on allylpiperazines from the LINS01 series suggested that this group could enhance H_3_R affinity though performing specific interactions with aromatic residues from this receptor,^26^ and possibly establish a π-interaction with tryptophan residues from both AChE and BChE,^25^ as previously observed for compound LINS01023 (Figure 2). However, except for compound **632**, the allylpiperazines (xx**2**) showed low affinity for both H_3_R and ChEs.

Regarding the lipophilic aromatic region, no remarkable differences were observed between carbon-containing or nitrogen-containing compounds, suggesting that the aromatic nitrogen does not play a major role to bind at ChEs (Figure 3A). Furthermore, variation in the linker length did not result notable differences in the inhibitory activities on ChEs (Figure 3B). To perform a more accurate analysis, ligand efficiency (LE)^35^ values were calculated to evaluate how the structural differences among the ligands contributed to the efficiency of their interaction with the targets (Table 2). Considering that acceptable LE values for drug candidates are typically greater than 0.3, the compounds exhibited low efficiency as ChEs inhibitors (LE 0.19–0.31), while their efficiency on the binding at H_3_R was generally higher, with values ranging from 0.26 to 0.35.

Although the modifications in the aromatic region did not directly influence the binding activity toward the targets, lipophilicity plays an important role in the efficiency of such binding. Since the compounds containing heteroaromatic moieties (**2**xx, **4**xx and **6**xx) are considerably less lipophilic than their carbon-containing counterparts (**1**xx, **3**xx and **5**xx, respectively), LE should be weighted by the modification on lipophilicity as indicated by lipophilic ligand efficiency (LLE).^35^

The impact of lipophilicity during development stages must be carefully evaluated, since excessive lipophilicity may lead to nonspecific hydrophobic interactions resulting in off-target effects related to poor drug-likeness and ADMET properties. Analysis of the LLE values for the heteroaromatic compounds showed higher efficiency for these compounds than for their carbon-containing analogues across all targets (Table 2). Considering that lipophilicity usually increases during development stages as a consequence of further structural modifications,^36^ less lipophilic molecules should be prioritized as lead structures in early stages. For instance, although compounds **313** (pIC_50_ 4.36) and **413** (pIC_50_ 4.31) present the same LE (0.21) values for the activity on AChE, their LLE values differ markedly (0.67 and 1.45, respectively), indicating that isoquinoline nitrogen of compound **413** increased its lipophilic efficiency in comparison to compound **313**, offering more opportunity for structural exploitation and improved drug-likeness. The LLE values were also higher for compounds **213** and **613** than for their matched pairs **113** and **513** (Table 2). Likewise, higher LLE values were observed for the efficiency of heteroaromatic compounds in both BChE and H_3_R; for example, compounds **416** and **616** showed higher LLE values than their naphthalene counterparts **316** and **516** in both targets.

Although compounds with longer linkers (x**3**x) displayed increased activity on BChE but not toward AChE, their efficiency values were penalized by the insertion of two heavy atoms. For example, compounds with shorter linkers (**413**, **611** and **612**) displayed higher efficiency values for AChE inhibition than their higher homologues (**433**, **631** and **632**, respectively). For instance, although compounds **612** and **632** have similar pIC_50_ values on BChE (4.11 and 4.10, respectively), the shorter homologue **612** (LLE 1.86) is more efficient than **632** (LLE 0.89). The increment in activity on ChEs for higher homologues was expected from literature ligands data,^37^ however the opposite was noted in H_3_R affinities, except for compound **632**. For example, the higher homologues **433** and **631** (p*K*_i_ < 5.0) had lower efficiency than their shorter counterparts **413** and **611**, (p*K*_i_ 6.41—LLE 3.55 and p*K*_i_ 6.45—LLE 4.05, respectively).

Regarding the basic region, LLE values for the benzylated compounds suggested that benzylpiperazines (xx**3**) were generally more efficient (due to their increased hydrophilicity) than the corresponding benzylpiperidines (xx**6**) at ChEs. For instance, compounds **313**, **413**, **513** and **613** showed higher LLE values than their piperidine analogues **316**, **416**, **516** and **616**. Conversely, this role is not clear for the efficiency at H_3_R. The 4-pyridyl substituent (xx**4**) provided comparable affinities to the benzylated analogues at H_3_R with decreased lipophilicity. For instance, compound **414** showed notably higher LLE values across all three targets, confirming the role observed for the 4-pyridyl substituent from previous literature reports.^24,38^

Importantly, the affinity and LLE values for both **413** and **414** denotes that these compounds present the best balanced mutitarget profile from the series. Although the activity of these compounds at AChE is about 100-fold lower than at H_3_R, it is important to emphasize that moderate inhibition at ChEs considerably potentiates the cholinergic effects produced by the antagonism at H_3_R, since H_3_R blockade significantly increases the ACh release from cholinergic neurons.^25,39^ This synergism was already observed in other literature reports (including on clinical studies),^11,40^ and it is recommended to avoid excessive cholinergic activity^41^ that may occur with dual H_3_R/ChEs inhibition.

### Molecular Docking Analyses

It was noted that compounds containing benzyl groups (xx**3** and xx**6**) in the basic region displayed superior pharmacological profiles across all targets. To unveil the potential role of this structural motif on each target, molecular docking studies were carried out at the *h*H_3_R, *h*AChE and *h*BChE.

Compounds **413** and **414** were used in docking experiments on the recently reported experimental structure of the *h*H_3_R (PDB 7F61).^42^ It is known that H_3_R possess two key acidic residues involved in the interaction with the natural agonist HA, the Asp114 and Glu206 (respectively 3.32 and 5.46 in Ballesteros numbering system), where the binding pocket was defined for the docking experiments. The compounds **413** and **414** have shown to bind at two opposite orientations in the binding pocket (Figure 4A,B) with similar frequencies and scores. In the first binding mode (Figure 4C,D), the protonated nitrogens from benzylamine (**413**) or 4-pyridyl (**414**) interact through ionic bond with the Asp114 (and complementary cation–π interactions with Tyr115 and Phe398) and the aromatic rings (benzyl or 4-pyridyl) demonstrate π contacts with Tyr374. The isoquinoline moiety performs π-interactions with Tyr94 and Glu395. This binding mode is similar to the observed with the experimental ligand PF-03654746.^42^

**Figure 4:**
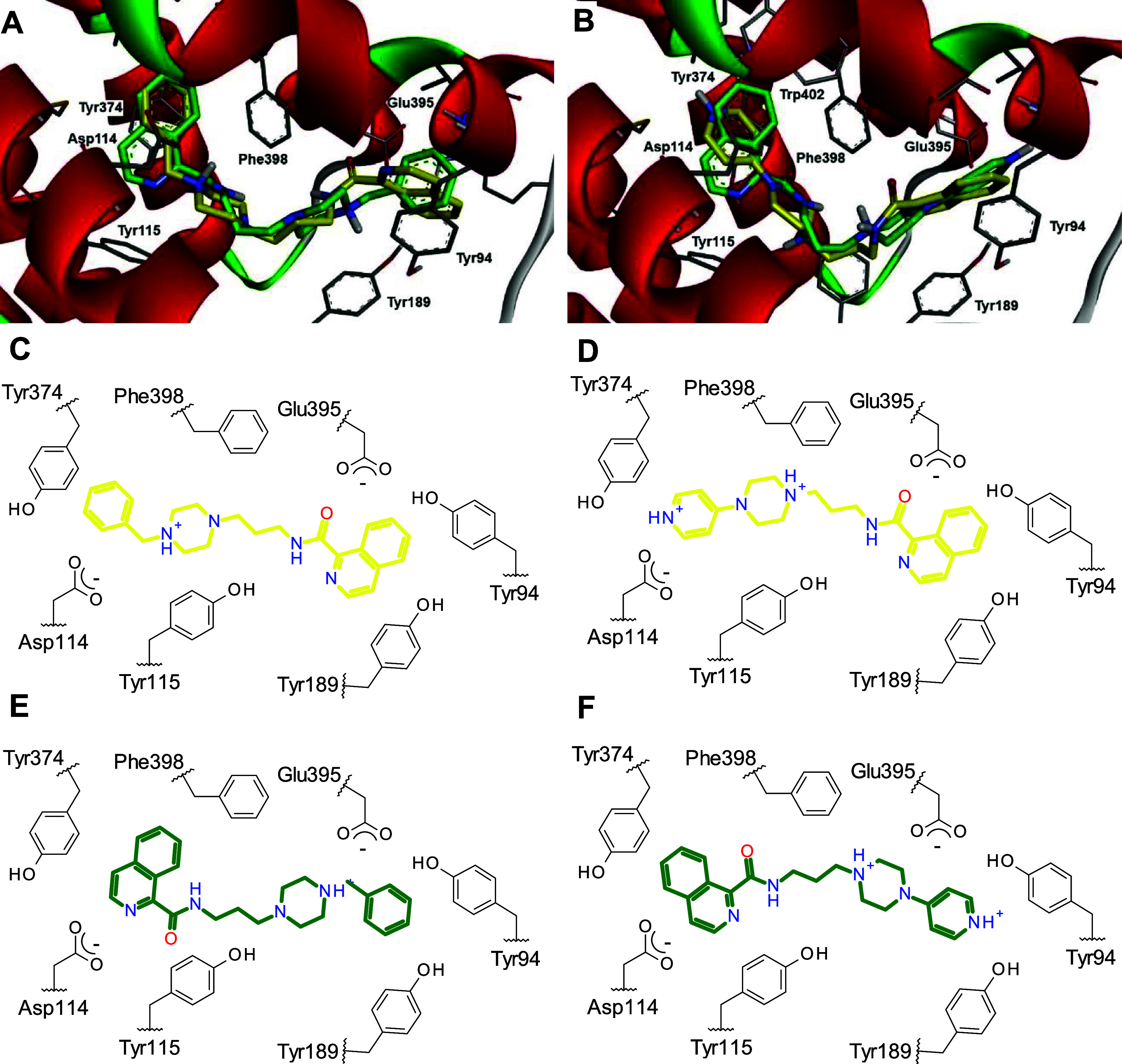
Representations of the binding orientations of the compounds **413** (A,C,E) and **414** (B,D,F) at *h*H_3_R. The structures in yellow (C,D) represent the first binding mode where the protonated nitrogens interact with Asp114, while the green structures (E,F) represent the second binding mode.

The second binding mode (Figure 4E,F) has an inverted orientation, where the protonated nitrogens from benzylamine (**413**) or 4-pyridyl (**414**) interact with Glu395. Additional π-interactions were identified with Tyr91, Tyr94 and Tyr189. The isoquinoline motif performed contacts with Asp114, Tyr374, Phe398 and Trp402. This inverted binding mode was already reported to other H_3_R ligands, however experimental data (including studies with Asp114Ala mutants) support that non-imidazole H_3_R ligands may bind in the first binding mode.^42^

Both ChEs are known to have key tryptophan residues in catalytic anionic site (CAS) and peripheral anionic site (PAS), where several inhibitors were reported to bind.^43–45^ The improved inhibitory potency of the benzyl-containing compounds (xx**3** and xx**6**) suggest that this motif may engage in specific interactions with the enzymes, possibly interacting with these tryptophan residues. The most potent compounds **613** and **516** were used in the docking experiments on human AChE (PDB 6O4W) and BChE (PDB 9R3C), respectively (Figure 5A,B). Considering its multitarget profile, compound **413** was also evaluated on AChE (Figure 5A).

**Figure 5:**
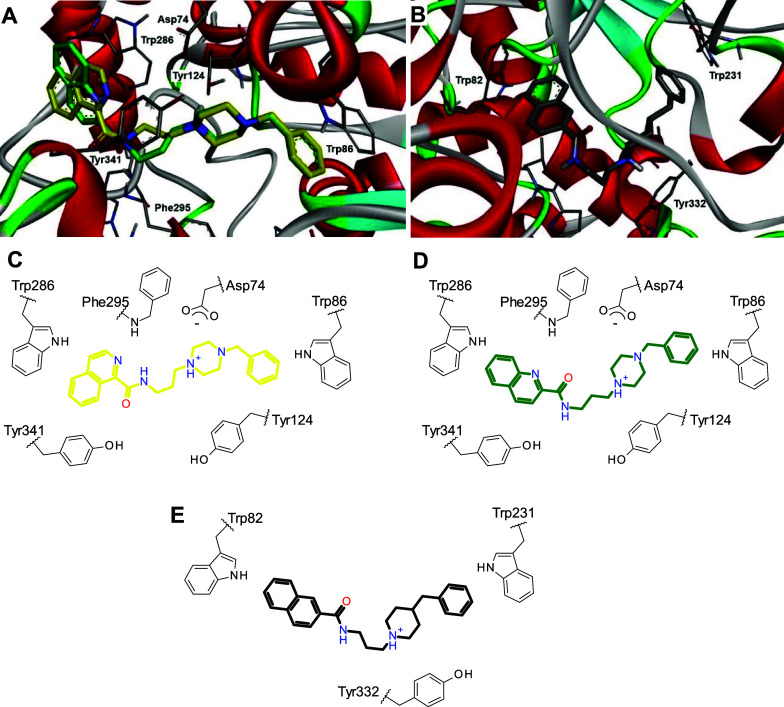
Representations of the binding orientations of the compounds **413** (yellow, A,C), **613** (green, A,D) and **516** (gray, E) at *h*AChE (A,C,D) and *h*BChE (B,E).

The results from docking experiments at AChE showed that compounds **613** and **413** may perform π interactions with both CAS and PAS tryptophan residues. The benzylamine group was involved in the interaction with Trp86 from CAS, while the (iso)­quinoline moiety performed contact with Trp286 from PAS. The piperazine nitrogens were also involved in hydrogen-bond and cation–π contacts with Asp74, Tyr124 and Tyr341 in a similar binding mode than the adopted by donepezil in the experimental crystal structure.^45^ Unfavorable clash between amide NH from **413** and Phe295 may explain its lower inhibitory potency, while compound **613** showed a favorable hydrogen-bond between amide carbonyl and Phe295, also observed for the indanone carbonyl from donepezil.^45^ Opposite orientations were also identified for both compounds (benzylamine toward Trp286 and quinoline toward Trp86), but this binding mode only occurred with lower frequency and scores. Docking results at BChE showed expected π interactions of benzyl and naphthyl groups from compound **516** with both CAS and PAS tryptophan residues (Trp82 and Trp231, respectively). The protonated piperidine nitrogen also performs cation–π interaction with Tyr332. This binding mode is quite similar to the obtained with the experimental ligand.^46^

### Kinetic Studies

To elucidate the mechanisms involved in the inhibition of ChEs, compounds **413**, **516** and **613** were selected for kinetic studies on AChE (**413** and **613**) and BChE (**516** and **613**). These assays were conducted using a range of inhibitor concentrations, and the results were analyzed by Lineweaver–Burk plots (Figure 6).^25^ Graphical analysis revealed that, for all tested inhibitors, the regression lines intersected above the *x*-axis (Figure 6). Specifically, increasing the inhibitor concentrations led to a decrease in the apparent maximum velocity (*V*_maxapp_) accompanied by an increase in the apparent Michaelis constant (*K*_Mapp_). This pattern is characteristic of mixed-type inhibition, indicating that these compounds interact with both the free enzyme and the enzyme–substrate complex, acting through competitive and noncompetitive mechanisms simultaneously, so as donepezil.^47–50^ This reinforces that the compounds may interact in both CAS and PAS, adopting a similar binding mode from donepezil in the binding site.

**Figure 6:**
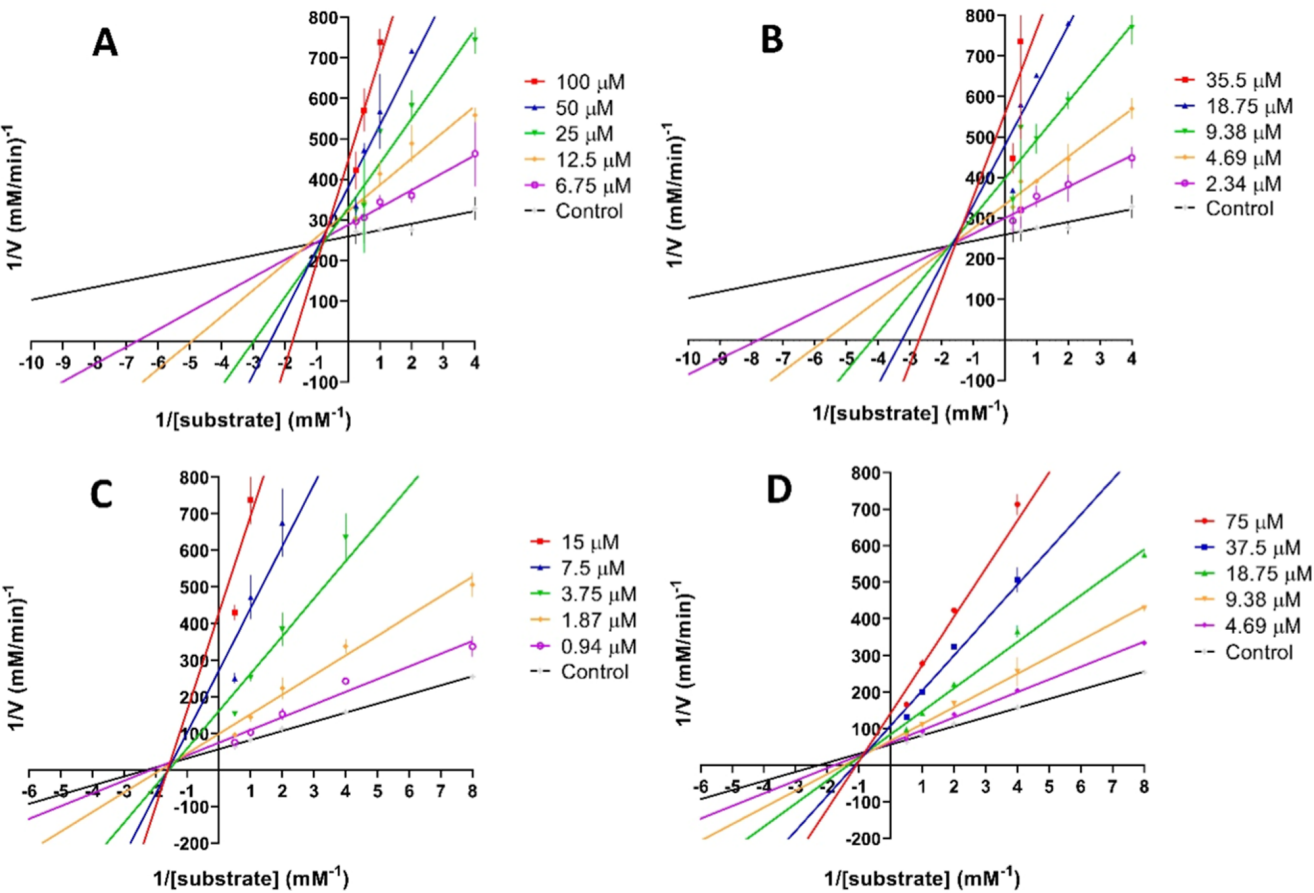
Lineweaver–Burk plots suggesting a mixed-type inhibition of *ee*AChE activity by compounds **413** (A) and **613** (B), and *eq*BChE activity by compounds **516** (C) and **613** (D). The plots are representative of four independent experiments performed in triplicates with different preparations of the compounds.

### Metal Chelating Activity

To assess the metal-chelating activity of the compounds toward copper and iron ions, UV–Vis absorption spectra were acquired and analyzed in the absence and presence of the respective metals.^32,33^ Except for compound **214**, the isoquinoline-based derivatives (**4**xx) within the aromatic lipophilic region demonstrated detectable metal-chelating properties (Table 1 and Figure 3A). Complex formation with copper ions (Cu^2+^) was observed for all isoquinoline-containing compounds (**4**xx), while compounds bearing the 4-pyridylpiperazine group also showed complexation with iron ions (Fe^2+^ and Fe^3+^).

The bathochromic shift of the carbonyl band from approximately 210 to 235 nm in the presence of copper ion was detected for the compounds **4**xx (but not for their naphthalene counterparts **3**xx), suggesting that copper may bind to the isoquinoline carboxamide moiety from these compounds. As illustrated in Figure 5, the absorption spectra of compound **413** (Figure 7A) exhibited a red shift of the carbonyl band upon Cu^2+^ addition, which was not detected for compound **313** (Figure 7D). Moreover, steric effects may influence the complex formation, since this behavior was not observed for the quinoline regioisomers (**6**xx, Figure 7C). This is exemplified by the absorption spectra obtained for compound **613**, which showed no detectable changes in presence or absence of metal ions.

**Figure 7:**
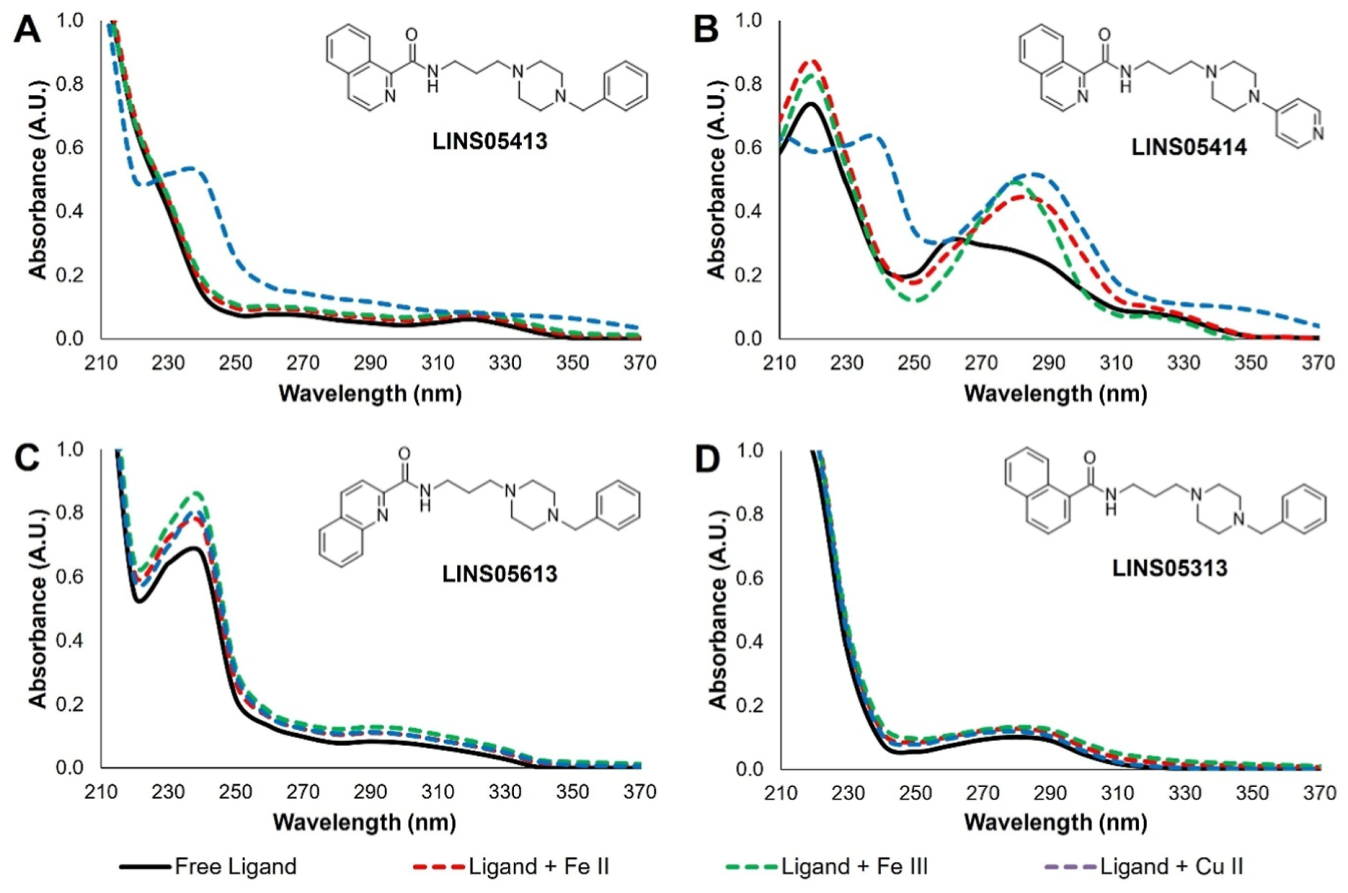
UV Absorption spectra of compounds **413** (A), **414** (B), **613** (C) and **313** (D) alone (50 μM, black lines) or in the presence of Fe^2+^ (red dashed lines), Fe^3+^ (green dashed lines) or Cu^2+^ (blue dashed lines) ions in equimolar concentrations.

Notably, complex formation with iron was observed exclusively for 4-pyridylpiperazine compounds, including the isoquinoline **414** (Figure 7B). Analysis of the absorption spectra indicates that this interaction specifically involves the 4-pyridyl moiety, as a bathochromic shift of the pyridine absorption band (from approximately 260 to 280 nm) was detected upon iron addition, whereas no such shift was observed for the benzylpiperazine analogues. In the case of compound **414**, the addition of copper ions induced bathochromic effects in both the carbonyl (∼240 nm) and pyridine (∼280 nm) regions, suggesting that complexation may occur at both coordination sites.

It is interesting to stress that metal dysregulation in the brain may lead to a cascade of events, pushing the brain to disease state and increasing the expression of several proteins that accelerate the cognitive decline, including ChEs.^22^ Therefore, the inhibitory activity toward these enzymes promoted by compounds **413** and **414** associated with their chelating activity may have a positive effect on neurodegenerative diseases, including AD and PD.

## Conclusion

In summary, the results provided valuable SAR insights for multitargeted agents toward H_3_R and ChEs bearing metal-chelating motifs. The benzylpiperazine motif enabled important interactions with both H_3_R and ChEs, providing a multitarget profile to the compounds due to specific π interactions with both targets. Moreover, the incorporation of chelating groups (especially the isoquinoline motif) provided chelating activity with improved drug-likeness and higher lipophilic efficiency, thus offering opportunities for further optimization on other structural motifs. Notably, compounds **LINS05413** and **LINS05414** exhibited a balanced multitarget profile, including metal-chelating activity with iron and copper, positioning them and the **LINS05** series scaffold as promising prototypes for the development of drug-like multitargeted ligands against neurodegenerative diseases.

## Methods

### Reagents and Equipment

Chemicals were obtained in adequate purity from Sigma-Aldrich Co. (Saint Louis, MO, USA) and LabSynth Co. (Diadema, Brazil) and used without any further purification. ^1^H and ^13^C NMR spectra were recorded on a Ultrashield 300 spectrometer (Bruker Daltonics, Bremen, Germany), operating at 300 and 75 MHz, respectively, using CDCl_3_ or DMSO-*d*_6_ as solvents and tetramethylsilane (TMS) as internal standard. Chemical shifts (δ) were determined from TMS and are reported in parts per million (ppm). Coupling constants (*J*) are reported in units of Hertz (Hz), if applicable. All NMR data were obtained using the compounds as free bases. HPLC-UV analysis was performed in a LC-20AT chromatograph with a RF20A UV–vis detector (Shimadzu Corp., Kyoto, Japan) for the hydrogenmaleate salts or for the free bases, when applicable, under the following conditions: stationary phase: C18; detection wavelength: 275 nm; flow rate: 1.0 mL/min; mobile phase: methanol/H_2_O. Data are reported as the percentage of each peak, compounds exhibiting chromatographic purity above 95% were considered suitable for the in vitro assays. HRMS analysis was performed for the hydrogenmaleate salts or for the free bases, when applicable, using a micrOTOF-Q ESI-Qq-TOF mass spectrometer (Bruker Daltonics, Bremen, Germany).

The Electrophorus electricus AChE (eeAChE) and BChE from equine serum (eqBChE) were obtained from Sigma-Aldrich Co. Stock solutions of the enzymes were prepared as indicated by the supplier. For cholinesterase assays, test compounds (as salts of maleic acid or free bases), donepezil hydrochloride and neostigmine mesylate were prepared as 10 mM stock solutions. The stock solutions were used in serial dilutions until reaching the desired concentrations for the assays. The assay was performed in a microplate spectrophotometer (PowerWave HT, BioTek).

The chelating activity assay was performed in a microplate spectrophotometer (PowerWave HT, Agilent BioTek, USA). Test compounds (as free bases) and the metals, FeSO_4_, FeCl_3_ and CuSO_4_, were evaluated for their ability to absorb UV–visible radiation at a concentration of 50 μM. The solutions were prepared in methanol (MeOH). The assays were carried out using UV-transparent 96-well microplates (UV-Star, Greiner Bio-One, Austria).

### Synthesis of the Compounds

#### Synthesis of Pyrazine-5-Carbonyl Chloride (Method A)^27,28^

In a flask 2-pyrazinoic acid (3 mmol) was added to 10 mL of DCM. The suspension was cooled in an ice bath under magnetic stirring and thionyl chloride (SOCl_2_—4.5 mmol) was slowly added to the flask. The reaction was heated at 50 °C for 3 h, when the solvent and excess of SOCl_2_ were removed under reduced pressure. The resulting solid was added directly to the following reaction for the preparation of amide intermediate **1b** and final compound **LINS05215**.

#### General Procedure for the Preparation of Amides **1a–1d** and **LINS05215** from Acyl Chlorides (Method B)^27^

In a flask, the adequate acyl chloride (benzoyl, pyrazine-2-carbonyl, naphthalene-1-carbonyl or naphthalene-2-carbonyl chloride, 3 mmol), the corresponding amine (3-chloropropylamine or 3-(1-piperidyl)­propylamine, 3.3 mmol) and TEA (3 mmol) were added to 15 mL of DCM. The mixture was stirred at room temperature for 5–10 h (TLC monitoring). The resulting solution was washed trice with 15 mL of saturated aqueous NaHCO_3_, the organic layer was dried over anhydrous Na_2_SO_4_ and evaporated. The crude product was purified through column chromatography using hexane/AcOEt (2:1) (**1a–1d**) or DCM/MeOH (9:1) (**LINS05215**) as eluent.

##### *N*-(3-Chloropropyl)­benzamide (**1a**)

Yellowish oil. Yield 90%. ^1^H NMR (300 MHz, CDCl_3_) δ: 7.84–7.71 (m, 2H), 7.53–7.43 (m, 1H), 7.43–7.32 (m, 2H), 6.99 (s, 1H), 3.66–3.50 (m, 4H), 2.08 (quint, *J* = 6.5 Hz, 2H). ^13^C NMR (75 MHz, CDCl_3_) δ: 167.9, 134.4, 131.5, 128.5, 127.0, 42.7, 37.6, 32.1.

##### *N*-(3-Chloropropyl)­pyrazine-2-carboxamide (**1b**)

White solid; mp 63–65 °C. Yield 48%. ^1^H NMR (300 MHz, CDCl_3_) δ: 9.41 (d, *J* = 1.4 Hz, 1H), 8.77 (d, *J* = 2.5 Hz, 1H), 8.54 (dd, *J* = 2.5, 1.4 Hz, 1H), 7.98 (s, 1H), 3.72–3.62 (m, 4H), 2.14 (quint, *J* = 6.5 Hz, 2H). ^13^C NMR (75 MHz, CDCl_3_) δ: 163.3, 147.4, 144.4, 144.3, 142.6, 42.3, 36.9, 32.1.

##### *N*-(3-Chloropropyl)­naphtalene-1-carboxamide (**1c**)

Yellowish solid; mp 54–56 °C. Yield 87%. ^1^H NMR (300 MHz, CDCl_3_) δ: 8.22–8.11 (m, 1H), 7.90–7.75 (m, 2H), 7.58–7.38 (m, 3H), 7.37–7.26 (m, 1H), 6.62 (s, 1H), 3.57–3.48 (m, 4H), 2.01 (quint, *J* = 6.5 Hz, 2H). ^13^C NMR (75 MHz, CDCl_3_) δ: 169.9, 134.2, 133.6, 130.6, 130.0, 128.3, 127.1, 126.4, 125.3, 124.9, 124.7, 42.6, 37.5, 32.1.

##### *N*-(3-Chloropropyl)­naphtalene-2-carboxamide (**1d**)

Yellowish solid; mp 63–64 °C. Yield 55%. ^1^H NMR (300 MHz, CDCl_3_) δ: 8.29 (s, 1H), 7.94–7.80 (m, 4H), 7.63–7.50 (m, 2H), 6.57 (s, 1H), 3.75–3.64 (m, 4H), 2.17 (quint, *J* = 6.5 Hz, 2H). ^13^C NMR (75 MHz, CDCl_3_) δ: 167.8, 134.7, 132.6, 131.5, 128.9, 128.5, 127.7, 127.7, 127.3, 126.8, 123.4, 42.7, 37.8, 32.1.

##### *N*-[3-(1-Piperidyl)­propyl]­pyrazine-2-carboxamide (**LINS05215**)

Yellowish solid; mp 50–52 °C. Yield 16%. ^1^H NMR (300 MHz, CDCl_3_) δ: 9.40 (d, *J* = 1.4 Hz, 1H), 9.23 (s, 1H), 8.73 (d, *J* = 2.4 Hz, 1H), 8.52 (dd, *J* = 2.4, 1.4 Hz, 1H), 3.58 (dd, *J* = 12.2, 5.5 Hz, 2H), 2.62–2.28 (m, 6H), 1.80 (quint, *J* = 5.5 Hz, 2H), 1.73–1.59 (m, 4H), 1.58–1.38 (m, 2H). ^13^C NMR (75 MHz, CDCl_3_) δ: 163.1, 146.9, 145.1, 144.4, 142.5, 58.4, 54.8, 39.8, 25.8, 25.2, 24.5. HRMS (ESI) *m*/*z*: [M + H]^+^ calcd.: 249.1709; [M + H]^+^ found: 249.1708. HPLC-UV: RT: 3.54 min (95%).^51^

#### General Procedure for the Preparation of Amides **1e–1h** and **LINS05415** from Carboxylic Acids (Method C)^27,30^

In a flask, equimolar amounts (3 mmol) of the adequate carboxylic acid (1-isoquinoline or 2-quinoline), *N*-ethyl-*N*′-(3-(dimethylamino)­propyl)­carbodiimide hydrochloride (EDC·HCl) and 1-hydroxybenzotriazole hydrate (HOBt·*x*H_2_O) were added to 15 mL of dichloromethane (DCM). The reaction mixture was stirred at room temperature for 1–2 h, then 3 mmol of appropriate amine (3-chloropropylamine, 5-amino-1-pentanol or 3-(1-piperidyl)­propylamine) were added. The reaction was kept under stirring for 18–24 h until completion (TLC monitoring). The resulting solution was washed with 3 × 15 mL of saturated aqueous NaHCO_3_ solution, the organic layer was dried over anhydrous Na_2_SO_4_ and evaporated. The crude product was purified through column chromatography using hexane/AcOEt (1:1) (**1e–1h**) or DCM/MeOH (20:1) (**LINS05415**) as eluent.

##### *N*-(3-Chloropropyl)­isoquinoline-1-carboxamide (**1e**)

Yellowish solid; mp 54–55 °C. Yield 38%. ^1^H NMR (300 MHz, CDCl_3_) δ: 9.59 (d, *J* = 7.9 Hz, 1H), 8.45 (d, *J* = 5.5 Hz, 1H), 8.40 (s, 1H), 7.93–7.76 (m, 2H), 7.76–7.56 (m, 2H), 3.72–3.66 (m, 2H), 3.70–3.69 (m, 2H), 2.18 (quint, *J* = 6.5 Hz, 2H). ^13^C NMR (75 MHz, CDCl_3_) δ: 166.4, 148.0, 140.2, 137.4, 130.5, 128.7, 127.8, 127.0, 126.8, 124.5, 42.6, 36.9, 32.3.

##### *N*-(3-Chloropropyl)­quinoline-2-carboxamide (**1f**)

Yellowish solid; mp 38–41 °C. Yield 46%. ^1^H NMR (300 MHz, CDCl_3_) δ: 8.42 (s, 1H), 8.35–8.26 (m, 2H), 8.11 (dd, *J* = 8.5, 1.1 Hz, 1H), 7.88 (dd, *J* = 8.2, 1.4 Hz, 1H), 7.77 (ddd, *J* = 8.5, 6.9, 1.4 Hz, 1H), 7.62 (ddd, *J* = 8.2, 6.9, 1.1 Hz, 1H), 3.76–3.64 (m, 2H), 3.73–3.68 (m, 2H), 2.19 (quint, *J* = 6.6 Hz, 2H). ^13^C NMR (75 MHz, CDCl_3_) δ: 164.8, 149.6, 146.5, 137.6, 130.2, 129.7, 129.4, 127.9, 127.8, 118.8, 42.5, 36.9, 32.4.

##### *N*-(5-Hydroxypentyl)­isoquinoline-1-carboxamide (**1g**)

Yellowish solid; mp 65–67 °C. Yield 37%. ^1^H NMR (300 MHz, CDCl_3_) δ: 9.53–9.48 (m, 1H), 8.43 (d, *J* = 5.5 Hz, 1H), 8.27 (s, 1H), 7.84–7.81 (m, 1H), 7.78 (d, *J* = 5.5 Hz, 1H), 7.74–7.61 (m, 2H), 3.65 (t, *J* = 6.3 Hz, 2H), 3.51 (dd, *J* = 13.2, 6.3 Hz, 2H), 2.63 (s, 1H), 1.76–1.56 (m, 4H), 1.56–1.41 (m, 2H). ^13^C NMR (75 MHz, CDCl_3_) δ: 166.3, 148.5, 140.2, 137.4, 130.5, 128.6, 127.7, 126.9, 126.8, 124.3, 62.5, 39.4, 32.3, 29.4, 23.2.

##### *N*-(5-Hydroxypentyl)­quinoline-2-carboxamide (**1h**)

Yellowish oil. Yield 41%. ^1^H NMR (300 MHz, CDCl_3_) δ: 8.36 (s, 1H), 8.29 (s, 2H), 8.10 (d, *J* = 8.2 Hz, 1H), 7.86 (d, *J* = 8.2 Hz, 1H), 7.79–7.71 (m, 1H), 7.65–7.56 (m, 1H), 3.67 (t, *J* = 6.5 Hz, 2H), 3.54 (dd, *J* = 13.5, 6.5 Hz, 2H), 2.34 (s, 1H), 1.79–1.58 (m, 4H), 1.57–1.43 (m, 2H). ^13^C NMR (75 MHz, CDCl_3_) δ: 164.6, 149.8, 146.4, 137.5, 130.1, 129.6, 129.3, 127.8, 127.6, 118.8, 62.5, 39.5, 32.3, 29.5, 23.2.

##### *N*-[3-(1-Piperidyl)­propyl]­isoquinoline-1-carboxamide (**LINS05415**)

Yellowish oil. Yield 19%. ^1^H NMR (300 MHz, CDCl_3_) δ: 9.54 (d, *J* = 7.5 Hz, 1H), 9.11 (s, 1H), 8.46 (d, *J* = 5.5 Hz, 1H), 7.83 (d, *J* = 7.5 Hz, 1H), 7.77 (d, *J* = 5.5 Hz, 1H), 7.74–7.58 (m, 2H), 3.61 (dd, *J* = 12.4, 6.6 Hz, 2H), 2.54 (t, *J* = 6.6 Hz, 2H), 2.52–2.33 (m, 4H), 1.88 (quint, *J* = 6.6 Hz, 2H), 1.72–1.55 (m, 4H), 1.53–1.35 (m, 2H). ^13^C NMR (75 MHz, CDCl_3_) δ: 166.3, 149.1, 140.3, 137.3, 130.4, 128.4, 127.9, 126.9, 126.7, 123.9, 57.9, 54.7, 39.3, 25.7, 25.6, 24.3. HRMS (ESI) *m*/*z*: [M + H]^+^ calcd.: 298.1913; [M + H]^+^ found: 298.1915. HPLC-UV: RT: 3.44 min (95%).

#### Synthesis of Tosylate Derivatives **1g′** and **1h′** (Method D)^31^

In a flask, amides **1g** or **1h** (1 mmol) and triethylamine (TEA, 1 mmol) were added to 10 mL of DCM. Then tosyl chloride (TsCl, 1 mmol) was added slowly to the reaction mixture. The reaction was kept under stirring at 50 °C for 12 h. The resulting solution was washed trice with 15 mL of saturated aqueous NaHCO_3_, the organic layer was dried over anhydrous Na_2_SO_4_ and evaporated. The crude product was purified through column chromatography using hexane/AcOEt (1:1) as eluent.

##### 5-(Isoquinoline-1-carbonylamino)­pentyl-4-methylbenzenesulfonate (**1g′**)

Yellowish oil. Yield 25%; ^1^H NMR (300 MHz, CDCl_3_) δ: 9.58 (d, *J* = 8.1 Hz, 1H), 8.44 (d, *J* = 5.5 Hz, 1H), 8.23 (s, 1H), 7.87–7.74 (m, 4H), 7.75–7.62 (m, 2H), 7.31 (d, *J* = 8.1 Hz, 2H), 4.04 (t, *J* = 6.6 Hz, 2H), 3.46 (dd, *J* = 13.3, 6.6 Hz, 2H), 2.40 (s, 3H), 1.79–1.66 (m, 2H), 1.65–1.58 (m, 2H), 1.53–1.36 (m, 2H). ^13^C NMR (75 MHz, CDCl_3_) δ: 164.5, 149.8, 146.5, 144.7, 137.5, 133.1, 130.1, 129.8, 129.7, 129.3, 128.0, 127.9, 127.8, 118.8, 70.3, 39.2, 29.1, 28.5, 22.9, 21.6.

##### 5-(Quinoline-2-carbonylamino)­pentyl-4-methylbenzenesulfonate (**1h′**)

Yellowish oil. Yield 45%; ^1^H NMR (300 MHz, CDCl_3_) δ: 8.36–8.23 (m, 3H), 8.11 (d, *J* = 8.3 Hz, 1H), 7.88 (dd, *J* = 8.3, 0.9 Hz, 1H), 7.83–7.72 (m, 3H), 7.67–7.57 (m, 1H), 7.32 (d, *J* = 8.0 Hz, 2H), 4.05 (t, *J* = 6.5 Hz, 2H), 3.49 (dd, *J* = 13.4, 6.5 Hz, 2H), 2.42 (s, 3H), 1.78–1.68 (m, 2H), 1.69–1.59 (m, 2H), 1.53–1.37 (m, 2H). ^13^C NMR (75 MHz, CDCl_3_) δ: 164.5, 149.8, 146.5, 144.7, 137.5, 133.1, 130.1, 129.8, 129.7, 129.3, 127.9, 127.8, 118.8, 70.3, 39.2, 29.1, 28.5, 22.9, 21.6.

#### General Procedure for the Alkylation of Amine Derivatives (Method E)^24^

The adequate amide intermediate (**1a–1f** or **1g′–1h′**, 1 mmol), KI (1 mmol), K_2_CO_3_ (1.5 mmol) and the amine (1-Boc-piperazine, 1-benzylpiperazine, 4-benzylpiperidine, homopiperazine, 1-propylpiperazine or 1-(4-pyridyl)­piperazine, 1.5 mmol) were added to 15 mL of AcN. The reaction was stirred at 80 °C up to 24 h (TLC monitoring) and afterward, the solvent was evaporated. The residue was taken up with 20 mL of AcOEt and washed trice with 15 mL of water. The organic layer was dried over anhydrous Na_2_SO_4_ and evaporated. The crude material was purified through column chromatography using DCM/MeOH (20:1) as eluent.

##### *tert*-Butyl-4-[3-(pyrazine-2-carbonylamino)­propyl]­piperazine-1-carboxylate (**2a**)

Reaction with **1b** gave a yellowish oil. Yield 48%. ^1^H NMR (300 MHz, CDCl_3_) δ: 9.40 (s, 1H), 9.03 (s, 1H), 8.74 (d, *J* = 2.1 Hz, 1H), 8.51 (s, 1H), 3.61 (dd, *J* = 11.9, 6.2 Hz, 2H), 3.55–3.46 (m, 4H), 2.55 (t, *J* = 6.2 Hz, 2H), 2.50–2.38 (m, 4H), 1.82 (quint, *J* = 6.2 Hz, 2H), 1.48 (s, 9H). ^13^C NMR (75 MHz, CDCl_3_) δ: 163.0, 154.7, 147.1, 144.8, 144.4, 142.5, 79.7, 57.7, 53.2, 43.5, 39.5, 28.4, 25.2.

##### *tert*-Butyl-4-[3-(naphthalene-1-carbonylamino)­propyl]­piperazine-1-carboxylate (**2b**)

Reaction with **1c** gave a White oil. Yield 63%. ^1^H NMR (300 MHz, CDCl_3_) δ: 8.35–8.26 (m, 1H), 7.91–7.80 (m, 2H), 7.64 (s, 1H), 7.57–7.45 (m, 3H), 7.45–7.37 (m, 1H), 3.58 (dd, *J* = 11.8, 6.3 Hz, 2H), 3.22–3.03 (m, 4H), 2.49 (t, *J* = 6.3 Hz, 2H), 2.38–2.26 (m, 4H), 1.78 (quint, *J* = 6.3 Hz, 2H), 1.42 (s, 9H). ^13^C NMR (75 MHz, CDCl_3_) δ: 169.5, 154.5, 134.9, 133.7, 130.4, 130.1, 128.3, 126.9, 126.3, 125.4, 124.7, 124.7, 79.7, 57.6, 52.9, 43.4, 39.9, 28.4, 25.1.

##### *tert*-Butyl-4-[3-(isoquinoline-1-carbonylamino)­propyl]­piperazine-1-carboxylate (**2c**)

Reaction with **1e** gave a yellowish oil. Yield 39%. ^1^H NMR (300 MHz, CDCl_3_) δ: 9.58 (d, *J* = 8.0 Hz, 1H), 9.04 (s, 1H), 8.44 (d, *J* = 5.5 Hz, 1H), 7.87–7.76 (m, 2H), 7.72–7.64 (m, 2H), 3.62 (dd, *J* = 12.3, 6.3 Hz, 2H), 3.55–3.41 (m, 4H), 2.56 (t, *J* = 6.3 Hz, 2H), 2.50–2.34 (m, 4H), 1.86 (quint, *J* = 6.3 Hz, 2H), 1.47 (s, 9H). ^13^C NMR (75 MHz, CDCl_3_) δ: 166.2, 154.8, 148.8, 140.2, 137.4, 130.4, 128.5, 127.9, 127.0, 126.8, 124.2, 79.6, 57.5, 53.2, 42.9, 39.2, 28.4, 25.7.

##### *tert*-Butyl-4-[3-(quinoline-2-carbonylamino)­propyl]­piperazine-1-carboxylate (**2d**)

Reaction with **1f** gave a yellowish oil. Yield 58%. ^1^H NMR (300 MHz, CDCl_3_) δ: 9.00 (s, 1H), 8.35–8.28 (m, 2H), 8.03 (d, *J* = 8.5 Hz, 1H), 7.94–7.84 (m, 1H), 7.80–7.69 (m, 1H), 7.65–7.55 (m, 1H), 3.65 (dd, *J* = 12.5, 6.3 Hz, 2H), 3.60–3.49 (m, 4H), 2.57 (t, *J* = 6.3 Hz, 2H), 2.51–2.40 (m, 4H), 1.87 (quint, *J* = 6.3 Hz, 2H), 1.47 (s, 9H). ^13^C NMR (75 MHz, CDCl_3_) δ: 164.6, 154.9, 150.2, 146.6, 137.4, 130.1, 129.4, 129.3, 127.8, 127.8, 119.1, 79.6, 57.4, 53.2, 43.2, 39.2, 28.4, 25.9.

##### *tert*-Butyl-4-[5-(quinoline-2-carbonylamino)­pentyl]­piperazine-1-carboxylate (**2e**)

Reaction with **1h′** gave a yellowish oil. Yield 86%. ^1^H NMR (300 MHz, CDCl_3_) δ: 8.39–8.26 (m, 3H), 8.10 (d, *J* = 8.4 Hz, 1H), 7.86 (d, *J* = 8.2 Hz, 1H), 7.75 (dt, *J* = 8.4, 1.2 Hz, 1H), 7.61 (dt, *J* = 8.2, 1.2 Hz, 1H), 3.54 (dd, *J* = 13.5, 6.6 Hz, 2H), 3.49–3.39 (m, 4H), 2.50–2.26 (m, 6H), 1.72 (quint, *J* = 6.6 Hz, 2H), 1.65–1.51 (m, 2H), 1.55–1.37 (m, 11H). ^13^C NMR (75 MHz, CDCl_3_) δ: 164.4, 154.7, 149.9, 146.4, 137.4, 130.0, 129.6, 129.2, 127.8, 127.7, 118.8, 79.5, 58.4, 53.0, 43.5, 39.4, 29.6, 28.4, 26.4, 24.9.

##### *N*-[3-(4-Benzylpiperazin-1-yl)­propyl]­benzamide (**LINS05113**)

Reaction with **1a** gave a yellowish oil. Yield 39%. ^1^H NMR (300 MHz, CDCl_3_) δ: 8.35 (s, 1H), 7.86–7.78 (m, 2H), 7.54–7.45 (m, 1H), 7.44–7.34 (m, 2H), 7.34–7.20 (m, 5H), 3.55 (dd, *J* = 11.8, 6.1 Hz, 2H), 3.50 (s, 2H), 2.64–2.29 (m, 10H), 1.76 (quint, *J* = 6.1 Hz, 2H). ^13^C NMR (75 MHz, CDCl_3_) δ: 167.4, 137.8, 134.8, 131.2, 129.2, 128.4, 128.4, 128.3, 127.2, 63.1, 58.3, 53.4, 52.9, 40.8, 24.3. HRMS (ESI) *m*/*z*: [M + H]^+^ calcd.: 338.2227; [M + H]^+^ found: 338.2215. HPLC-UV: RT: 5.07 min (95%).

##### *N*-[3-(4-Benzylpiperazin-1-yl)­propyl]­pyrazine-2-carboxamide (**LINS05213**)

Reaction with **1b** gave a yellowish oil. Yield 64%. ^1^H NMR (300 MHz, CDCl_3_) δ: 9.39 (d, *J* = 1.5 Hz, 1H), 9.09 (s, 1H), 8.72 (d, *J* = 2.4 Hz, 1H), 8.37 (dd, *J* = 2.4, 1.5 Hz, 1H), 7.38–7.23 (m, 5H), 3.64–3.51 (m, 4H), 2.69–2.40 (m, 10H), 1.79 (quint, *J* = 6.1 Hz, 2H). ^13^C NMR (75 MHz, CDCl_3_) δ: 163.1, 146.9, 144.9, 144.4, 142.5, 137.9, 129.3, 128.3, 127.1, 63.2, 57.8, 53.4, 52.8, 39.7, 25.1. HRMS (ESI) *m*/*z*: [M + H]^+^ calcd.: 340.2131; [M + H]^+^ found: 340.2123. HPLC-UV: RT: 3.66 min (95%).

##### *N*-[3-[4-(4-Pyridyl)­piperazin-1-yl]­propyl]­benzamide (**LINS05214**)

Reaction with **1b** gave a yellowish oil. Yield 19%. ^1^H NMR (300 MHz, CDCl_3_) δ: 9.40 (d, *J* = 1.4 Hz, 1H), 9.08 (s, 1H), 8.69 (d, *J* = 2.4 Hz, 1H), 8.33 (dd, *J* = 2.4, 1.4 Hz, 1H), 8.30 (dd, *J* = 5.0, 1.5 Hz, 2H), 6.68 (dd, *J* = 5.0, 1.5 Hz, 2H), 3.63 (dd, *J* = 11.8, 6.2 Hz, 2H), 3.50–3.34 (m, 4H), 2.71–2.55 (m, 6H), 1.85 (quint, *J* = 6.2 Hz, 2H). ^13^C NMR (75 MHz, CDCl_3_) δ: 163.0, 155.0, 150.4, 147.2, 144.8, 144.5, 142.4, 108.4, 57.6, 52.8, 45.9, 39.6, 25.2. HRMS (ESI) *m*/*z*: [M + H]^+^ calcd.: 327.1927; [M + H]^+^ found: 327.1932. HPLC-UV: RT: 2.51 min (95%).

##### *N*-[3-(4-Benzylpiperazin-1-yl)­propyl]­naphthalene-1-carboxamide (**LINS05313**)

Reaction with **1c** gave a yellowish oil. Yield 28%. ^1^H NMR (300 MHz, CDCl_3_) δ: 8.38–8.30 (m, 1H), 8.22 (s, 1H), 7.97–7.81 (m, 2H), 7.60–7.54 (m, 1H), 7.55–7.46 (m, 2H), 7.47–7.39 (m, 1H), 7.35–7.13 (m, 5H), 3.60 (dd, *J* = 11.4, 5.9 Hz, 2H), 3.20 (s, 2H), 2.72–1.85 (m, 10H), 1.75 (quint, *J* = 5.9 Hz, 2H). ^13^C NMR (75 MHz, CDCl_3_) δ: 169.4, 137.9, 135.2, 133.7, 130.3, 130.2, 129.2, 128.3, 128.2, 127.1, 126.9, 126.3, 125.7, 125.1, 124.7, 62.9, 57.9, 53.0, 52.8, 40.6, 24.6. HRMS (ESI) *m*/*z*: [M + H]^+^ calcd.: 388.2383; [M + H]^+^ found: 388.2382. HPLC-UV: RT: 3.81 min (95%).

##### *N*-[3-(4-Benzyl-1-piperidyl)­propyl]­naphthalene-1-carboxamide (**LINS05316**)

Reaction with **1c** gave a yellowish oil. Yield 36%. ^1^H NMR (300 MHz, CDCl_3_) δ: 8.48 (s, 1H), 8.40–8.28 (m, 1H), 7.96–7.80 (m, 2H), 7.60 (dd, *J* = 7.0, 0.9 Hz, 1H), 7.55–7.47 (m, 2H), 7.44 (dd, *J* = 8.0, 7.0 Hz, 1H), 7.28–7.11 (m, 3H), 7.03–6.91 (m, 2H), 3.57 (dd, *J* = 11.2, 5.7 Hz, 2H), 2.84 (d, *J* = 11.6 Hz, 2H), 2.50 (t, *J* = 5.7 Hz, 2H), 2.20 (d, *J* = 6.7 Hz, 2H), 1.89–1.69 (m, 4H), 1.44–1.25 (m, 3H), 0.83–0.63 (m, 2H). ^13^C NMR (75 MHz, CDCl_3_) δ: 169.5, 140.3, 135.2, 133.7, 130.3, 130.2, 129.0, 128.3, 128.2, 126.9, 126.3, 125.9, 125.7, 125.1, 124.8, 57.8, 53.5, 42.9, 40.4, 37.3, 31.6, 24.5. HRMS (ESI) *m*/*z*: [M + H]^+^ calcd.: 387.2430; [M + H]^+^ found: 387.2424. HPLC-UV: RT: 6.39 min (95%).

##### *N*-[3-(4-Benzylpiperazin-1-yl)­propyl]­isoquinoline-1-carboxamide (**LINS05413**)

Reaction with **1e** gave a yellowish oil. Yield 53%. ^1^H NMR (300 MHz, CDCl_3_) δ: 9.60–9.49 (m, 1H), 9.03 (s, 1H), 8.41 (d, *J* = 5.5 Hz, 1H), 7.88–7.81 (m, 1H), 7.78 (d, *J* = 5.5 Hz, 1H), 7.75–6.61 (m, 2H), 7.40–7.15 (m, 5H), 3.60 (dd, *J* = 12.4, 6.2 Hz, 2H), 3.52 (s, 2H), 2.81–1.99 (m, 10H), 1.83 (quint, *J* = 6.2 Hz, 2H). ^13^C NMR (75 MHz, CDCl_3_) δ: 166.3, 149.1, 140.3, 137.8, 137.3, 130.5, 129.4, 128.5, 128.3, 127.8, 127.2, 126.9, 126.8, 124.1, 63.2, 57.4, 53.2, 52.9, 39.3, 25.6. HRMS (ESI) *m*/*z*: [M + H]^+^ calcd.: 389.2335; [M + H]^+^ found: 389.2332. HPLC-UV: RT: 8.31 min (95%).

##### *N*-[3-[4-(4-Pyridyl)­piperazin-1-yl]­propyl]­isoquinoline-1-carboxamide (**LINS05414**)

Reaction with **1e** gave a yellowish oil. Yield 15%. ^1^H NMR (300 MHz, CDCl_3_) δ: 9.62–9.55 (m, 1H), 9.06 (s, 1H), 8.34 (d, *J* = 5.4 Hz, 1H), 8.27 (dd, *J* = 5.5, 1.2 Hz, 2H), 7.86–7.81 (m, 1H), 7.75 (d, *J* = 5.4 Hz, 1H), 7.73–7.63 (m, 2H), 6.72 (dd, *J* = 5.5, 1.2 Hz, 2H), 3.65 (dd, *J* = 11.8, 6.1 Hz, 2H), 3.51–3.39 (m, 4H), 2.70–2.55 (m, 6H), 1.90 (quint, *J* = 6.1 Hz, 2H). ^13^C NMR (75 MHz, CDCl_3_) δ: 166.2, 156.3, 148.6, 143.3, 140.00, 137.4, 130.6, 128.7, 127.8, 127.0, 126.8, 124.4, 107.5, 56.9, 52.4, 46.1, 38.8, 25.9. HRMS (ESI) *m*/*z*: [M + H]^+^ calcd.: 376.2131; [M + H]^+^ found: 376.2129. HPLC-UV: RT: 4.35 min (95%).

##### *N*-[3-(4-Benzyl-1-piperidyl)­propyl]­isoquinoline-1-carboxamide (**LINS05416**)

Reaction with **1e** gave a yellowish oil. Yield 58%. ^1^H NMR (300 MHz, CDCl_3_) δ: 9.58–9.48 (m, 1H), 9.09 (s, 1H), 8.46 (d, *J* = 5.5 Hz, 1H), 7.88–7.82 (m, 1H), 7.79 (d, *J* = 5.5 Hz, 1H), 7.76–7.62 (m, 2H), 7.32–7.23 (m, 2H), 7.23–7.09 (m, 3H), 3.60 (dd, *J* = 12.3, 5.9 Hz, 2H), 2.99 (d, *J* = 11.6 Hz, 2H), 2.58–2.47 (m, 4H), 1.99–1.77 (m, 4H), 1.67–1.45 (m, 3H), 1.46–1.28 (m, 2H). ^13^C NMR (75 MHz, CDCl_3_) δ: 166.3, 149.3, 140.6, 140.3, 137.33, 130.4, 129.1, 128.4, 128.2, 127.9, 126.9, 126.7, 125.8, 124.0, 57.7, 54.0, 43.3, 39.3, 37.9, 32.0, 25.8. HRMS (ESI) *m*/*z*: [M + H]^+^ calcd.: 388.2383; [M + H]^+^ found: 388.2366. HPLC-UV: RT: 6.72 min (95%).

##### *N*-[3-(Azepan-1-yl)­propyl]­isoquinoline-1-carboxamide (**LINS05417**)

Reaction with **1e** gave a yellowish oil. Yield 54%. ^1^H NMR (300 MHz, CDCl_3_) δ: 9.56–9.45 (m, 1H), 8.95 (s, 1H), 8.46 (d, *J* = 5.5 Hz, 1H), 7.84 (d, *J* = 7.9 Hz, 1H), 7.77 (d, *J* = 5.6 Hz, 1H), 7.74–7.61 (m, 2H), 3.62 (dd, *J* = 12.3, 6.3 Hz, 2H), 2.77–2.59 (m, 6H), 1.86 (quint, *J* = 6.3 Hz, 2H), 1.76–1.51 (m, 8H). ^13^C NMR (75 MHz, CDCl_3_) δ: 166.3, 149.2, 140.3, 137.3, 130.4, 128.4, 127.9, 126.9, 126.7, 123.9, 57.2, 55.8, 39.2, 27.6, 26.9, 26.5. HRMS (ESI) *m*/*z*: [M + H]^+^ calcd.: 312.2070; [M + H]^+^ found: 312.2068. HPLC-UV: RT: 6.35 min (95%).

##### *N*-[5-(4-Benzylpiperazin-1-yl)­pentyl]­isoquinoline-1-carboxamide (**LINS05433**)

Reaction with **1g′** gave a yellowish oil. Yield 65%. ^1^H NMR (300 MHz, CDCl_3_) δ: 9.64–9.54 (m, 1H), 8.45 (d, *J* = 5.5 Hz, 1H), 8.24 (t, *J* = 4.9 Hz, 1H), 7.88–7.82 (m, 1H), 7.79 (d, *J* = 5.5 Hz, 1H), 7.76–7.62 (m, 2H), 7.37–7.19 (m, 5H), 3.57–3.45 (m, 4H), 2.79–2.10 (m, 10H), 1.77–1.64 (m, 2H), 1.64–1.51 (m, 2H), 1.51–1.35 (m, 2H). ^13^C NMR (75 MHz, CDCl_3_) δ: 166.1, 148.4, 140.2, 137.9, 137.4, 130.5, 129.3, 128.6, 128.2, 127.9, 127.1, 127.0, 126.8, 124.3, 63.1, 58.5, 53.2, 52.9, 39.4, 29.6, 26.6, 25.1. HRMS (ESI) *m*/*z*: [M + H]^+^ calcd.: 417.2648; [M + H]^+^ found: 417.2645. HPLC-UV: RT: 3.37 min (95%).

##### *N*-[3-(4-Benzylpiperazin-1-yl)­propyl]­naphthalene-2-carboxamide (**LINS05513**)

Reaction with **1d** gave a yellowish oil. Yield 46%. ^1^H NMR (300 MHz, CDCl_3_) δ: 8.55 (sl, 1H), 8.35 (s, 1H), 8.12–7.79 (m, 4H), 7.75–7.50 (m, 2H), 7.45–7.19 (m, 5H), 3.61 (dd, *J* = 10.9, 5.4 Hz, 2H), 3.44 (s, 2H), 2.93–2.24 (m, 10H), 1.80 (quint, *J* = 5.4 Hz, 2H). ^13^C NMR (75 MHz, CDCl_3_) δ: 167.5, 137.8, 134.7, 132.7, 132.3, 129.2, 128.9, 128.3, 128.2, 127.8, 127.4, 127.4, 127.2, 126.6, 124.1, 63.1, 58.5, 53.4, 53.0, 41.1, 24.2. HRMS (ESI) *m*/*z*: [M + H]^+^ calcd.: 388.2383; [M + H]^+^ found: 388.2365. HPLC-UV: RT: 13.16 min (95%).

##### *N*-[3-(4-Benzyl-1-piperidyl)­propyl]­naphthalene-2-carboxamide (**LINS05516**)

Reaction with **1d** gave a yellowish oil. Yield 49%. ^1^H NMR (300 MHz, CDCl_3_) δ: 8.79 (sl, 1H), 8.36 (s, 1H), 8.02–7.84 (m, 4H), 7.62–7.50 (m, 2H), 7.31–7.11 (m, 3H), 7.08–6.98 (m, 2H), 3.63 (dd, *J* = 11.0, 5.6 Hz, 2H), 3.05 (d, *J* = 11.7 Hz, 2H), 2.67–2.54 (m, 2H), 2.45 (d, *J* = 7.0 Hz, 2H), 1.93 (t, *J* = 11.0 Hz, 2H), 1.83 (quint, *J* = 5.6 Hz, 2H), 1.71–1.45 (m, 3H), 1.38–1.17 (m, 2H). ^13^C NMR (75 MHz, CDCl_3_) δ: 167.6, 140.3, 134.7, 132.7, 132.4, 129.0, 128.9, 128.2, 128.2, 127.8, 127.4, 127.4, 126.6, 125.9, 124.2, 58.6, 54.0, 43.0, 41.0, 37.7, 32.0, 24.2. HRMS (ESI) *m*/*z*: [M + H]^+^ calcd.: 387.2430; [M + H]^+^ found: 387.2412. HPLC-UV: RT: 8.66 min (95%).

##### *N*-[3-(4-Propylpiperazin-1-yl)­propyl]­quinoline-2-carboxamide (**LINS05611**)

Reaction with **1f** gave a colorless oil. Yield 31%. ^1^H NMR (300 MHz, CDCl_3_) δ: 8.72 (s, 1H), 8.31 (s, 2H), 8.15 (d, *J* = 8.3 Hz, 1H), 7.89 (dd, *J* = 8.5, 1.1 Hz, 1H), 7.76 (ddd, *J* = 8.3, 7.0, 1.1 Hz, 1H), 7.62 (ddd, *J* = 8.5, 7.0, 0.8 Hz, 1H), 3.63 (dd, *J* = 11.8, 6.5 Hz, 2H), 3.02–2.52 (m, 10H), 2.45–2.36 (m, 2H), 1.88 (quint, *J* = 6.5 Hz, 2H), 1.62–1.46 (m, 2H), 0.90 (t, *J* = 7.4 Hz, 3H). ^13^C NMR (75 MHz, CDCl_3_) δ: 164.6, 150.1, 146.5, 137.4, 129.9, 129.7, 129.3, 127.8, 127.8, 119.0, 60.3, 56.8, 52.9, 52.8, 38.7, 26.2, 19.6, 11.8. HRMS (ESI) *m*/*z*: [M + H]^+^ calcd.: 341.2335; [M + H]^+^ found: 341.2333. HPLC-UV: RT: 7.00 min (95%).

##### *N*-[3-(4-Benzylpiperazin-1-yl)­propyl]­quinoline-2-carboxamide (**LINS05613**)

Reaction with **1f** gave a white solid; mp 64–66 °C. Yield 51%. ^1^H NMR (300 MHz, CDCl_3_) δ: 8.67 (s, 1H), 8.23 (s, 2H), 8.11 (d, *J* = 8.3 Hz, 1H), 7.82 (d, *J* = 8.3 Hz, 1H), 7.76–7.67 (m, 1H), 7.61–7.51 (m, 1H), 7.37–7.10 (m, 5H), 3.55 (dd, *J* = 11.8, 6.1 Hz, 2H), 3.45 (s, 2H), 2.69–2.35 (m, 10H), 1.89–1.74 (m, 2H). ^13^C NMR (75 MHz, CDCl_3_) δ: 164.6, 150.2, 146.5, 137.8, 137.4, 129.9, 129.8, 129.3, 129.3, 128.2, 127.8, 127.8, 127.1, 119.0, 62.9, 56.8, 53.1, 52.8, 38.8, 26.2. HRMS (ESI) *m*/*z*: [M + H]^+^ calcd.: 389.2335; [M + H]^+^ found: 389.2327. HPLC-UV: RT: 4.02 min (95%).

##### *N*-[3-(4-Benzyl-1-piperidyl)­propyl]­quinoline-2-carboxamide (**LINS05616**)

Reaction with **1f** gave a white solid; mp 62–63 °C. Yield 48%. ^1^H NMR (300 MHz, CDCl_3_) δ: 8.87 (s, 1H), 8.30 (sl, 2H), 8.18 (d, *J* = 8.4 Hz, 1H), 7.89 (d, *J* = 8.1 Hz, 1H), 7.78 (dt, *J* = 8.1, 1.5 Hz, 1H), 7.71–7.58 (m, 1H), 7.33–7.06 (m, 5H), 3.72–3.55 (m, 2H), 2.99 (d, *J* = 10.6 Hz, 2H), 2.63–2.44 (m, 4H), 2.00–1.79 (m, 4H), 1.64 (d, *J* = 12.2 Hz, 2H), 1.59–1.31 (m, 3H). ^13^C NMR (75 MHz, CDCl_3_) δ: 164.7, 150.3, 146.6, 140.7, 137.3, 129.9, 129.8, 129.3, 129.1, 128.2, 127.8, 127.8, 125.8, 119.1, 57.5, 54.1, 43.1, 39.1, 38.0, 32.0, 26.3. HRMS (ESI) *m*/*z*: [M + H]^+^ calcd.: 388.2383; [M + H]^+^ found: 388.2372. HPLC-UV: RT: 9.23 min (95%).

##### *N*-[5-(4-Propylpiperazin-1-yl)­pentyl]­quinoline-2-carboxamide (**LINS05631**)

Reaction with **1f** gave a white solid; mp 86–87 °C. Yield 60%. ^1^H NMR (300 MHz, CDCl_3_) δ: 8.40–8.21 (m, 3H), 8.11 (d, *J* = 8.5 Hz, 1H), 7.88 (dd, *J* = 8.2, 0.8 Hz, 1H), 7.80–7.72 (m, 1H), 7.65–7.58 (m, 1H), 3.54 (dd, *J* = 12.4, 6.8 Hz, 2H), 2.58–2.17 (m, 12H), 1.78–1.67 (m, 2H), 1.64–1.40 (m, 6H), 0.89 (t, *J* = 7.4 Hz, 3H). ^13^C NMR (75 MHz, CDCl_3_) δ: 164.4, 149.9, 146.5, 137.4, 130.0, 129.6, 129.3, 127.8, 127.8, 118.9, 60.7, 58.5, 53.2, 53.2, 39.5, 29.7, 26.6, 25.0, 19.9, 11.9. HRMS (ESI) *m*/*z*: [M + H]^+^ calcd.: 369.2648; [M + H]^+^ found: 369.2642. HPLC-UV: RT: 3.28 min (95%).

#### General Procedure for the Preparation of Intermediates **3a** and **3b** and Final Compounds **LINS05210**, **LINS05310** and **LINS05410** (Method F)

In a flask, the adequate 1-Boc-piperazine derivative, **2a** to **2e** (1 mmol) and trifluoroacetic acid (TFA, 3 mmol), dissolved in 0.5 mL of water, were added to 6 mL of DCM. The reaction was stirred at room temperature for 12–18 h (TLC monitoring) and afterward, the solvent was evaporated. The residue was taken up with 20 mL of water and washed with 15 mL of AcOEt. The aqueous layer was alkalinized (pH ∼11) and extracted with 3 × 15 mL of DCM. The organic layer was dried over anhydrous Na_2_SO_4_ and evaporated. The crude material was purified through column chromatography using DCM/MeOH (10:1) as eluent.

##### *N*-(3-Piperazin-1-ylpropyl)­pyrazine-2-carboxamide (**LINS05210**)

Reaction with **2a** gave a yellowish oil. Yield 58%. ^1^H NMR (300 MHz, CDCl_3_) δ: 9.40 (s, 1H), 9.09 (s, 1H), 8.74 (d, *J* = 1.5 Hz, 1H), 8.58 (s, 1H), 3.60 (dd, *J* = 11.4, 5.6 Hz, 2H), 3.18–3.05 (m, 1H), 3.05–2.95 (m, 4H), 2.66–2.30 (m, 6H), 1.81 (quint, *J* = 5.6, 2H). ^13^C NMR (75 MHz, CDCl_3_) δ: 163.1, 147.1, 144.9, 144.4, 142.6, 58.2, 54.0, 45.4, 39.6, 24.9. HRMS (ESI) *m*/*z*: [M + H]^+^ calcd.: 250.1662; [M + H]^+^ found: 250.1664. HPLC-UV: RT: 2.78 min (95%).^51^,

##### *N*-(3-Piperazin-1-ylpropyl)­naphthalene-1-carboxamide (**LINS05310**)

Reaction with **2b** gave a colorless oil. Yield 40%. ^1^H NMR (300 MHz, CDCl_3_) δ: 8.37–8.30 (m, 1H), 7.92–7.83 (m, 3H), 7.59 (dd, *J* = 7.0, 1.1 Hz, 1H), 7.57–7.47 (m, 2H), 7.44 (dd, *J* = 8.1, 1.1 Hz, 1H), 3.63 (dd, *J* = 11.7, 6.2 Hz, 2H), 2.76–2.57 (m, 5H), 2.52 (t, *J* = 6.2 Hz, 2H), 2.47–2.32 (m, 4H), 1.81 (quint, *J* = 6.2 Hz, 2H). ^13^C NMR (75 MHz, CDCl_3_) δ: 169.5, 135.0, 133.7, 130.4, 130.2, 128.3, 126.9, 126.3, 125.5, 124.8, 124.7, 58.2, 53.8, 45.5, 40.3, 24.7. HRMS (ESI) *m*/*z*: [M + H]^+^ calcd.: 298.1913; [M + H]^+^ found: 298.1917. HPLC-UV: RT: 4.29 min (95%).

##### *N*-(3-Piperazin-1-ylpropyl)­isoquinoline-1-carboxamide (**LINS05410**)

Reaction with **2c** gave a yellowish oil. Yield 80%. ^1^H NMR (300 MHz, CDCl_3_) δ: 9.56 (d, *J* = 9.0 Hz, 1H), 9.13 (s, 1H), 8.50 (d, *J* = 5.5 Hz, 1H), 7.88–7.75 (m, 2H), 7.76–7.56 (m, 2H), 5.01 (s, 1H), 3.62 (dd, *J* = 12.0, 6.1 Hz, 2H), 3.14–2.95 (m, 4H), 2.74–2.35 (m, 6H), 1.84 (quint, *J* = 6.1 Hz, 2H). ^13^C NMR (75 MHz, CDCl_3_) δ: 166.2, 148.8, 140.3, 137.3, 130.4, 128.5, 127.8, 126.9, 126.8, 124.2, 57.8, 52.9, 44.9, 39.3, 25.3. HRMS (ESI) *m*/*z*: [M + H]^+^ calcd.: 299.1866; [M + H]^+^ found: 299.1869. HPLC-UV: RT: 5.77 min (95%).

##### *N*-(3-Piperazin-1-ylpropyl)­quinoline-2-carboxamide (**3a**)

Reaction with **2d** gave a white solid; mp 85–86 °C. Yield 59%. ^1^H NMR (300 MHz, CDCl_3_) δ: 8.95 (s, 1H), 8.36–8.24 (m, 2H), 8.12 (d, *J* = 8.3 Hz, 1H), 7.87 (d, *J* = 8.3 Hz, 1H), 7.83–7.71 (m, 1H), 7.66–7.54 (m, 1H), 3.63 (dd, *J* = 12.4, 6.4 Hz, 2H), 3.08–2.90 (m, 4H), 2.60–2.30 (m, 7H), 1.86 (quint, *J* = 6.4 Hz, 2H). ^13^C NMR (75 MHz, CDCl_3_) δ: 164.6, 150.2, 146.6, 137.4, 130.1, 129.5, 129.2, 127.8, 127.7, 119.0, 57.9, 54.8, 46.0, 39.2, 25.8.

##### *N*-(5-Piperazin-1-ylpentyl)­quinoline-2-carboxamide (**3b**)

Reaction with **2e** gave a colorless oil. Yield 78%. ^1^H NMR (300 MHz, CDCl_3_) δ: 8.41–8.26 (m, 3H), 8.10 (d, *J* = 8.1 Hz, 1H), 7.87 (d, *J* = 8.1 Hz, 1H), 7.80–7.71 (m, 1H), 7.65–7.55 (m, 1H), 3.54 (dd, *J* = 12.6, 6.8 Hz, 2H), 2.97–2.82 (m, 4H), 2.57–2.40 (m, 5H), 2.33 (t, *J* = 6.8 Hz, 2H), 1.80–1.64 (m, 2H), 1.64–1.50 (m, 2H), 1.50–1.36 (m, 2H). ^13^C NMR (75 MHz, CDCl_3_) δ: 164.4, 149.9, 146.4, 137.4, 130.0, 129.6, 129.2, 127.8, 127.7, 118.8, 59.1, 54.4, 45.9, 39.5, 29.6, 26.3, 24.9.

#### General Procedure for the Preparation of Allyl Compounds **LINS05412**, **LINS05612** and **LINS05632** (Method G)^26^

In a flask, the piperazine derivatives LINS05410, **3a** or **3b** (0.3 mmol), K_2_CO_3_ and allyl bromide (1 equiv. to the respective piperazine derivative) were added to 15 mL of THF. The reaction was stirred under room temperature up to 24 h (TLC monitoring) and afterward, the solvent was evaporated. The residue was taken up with 20 mL of AcOEt and washed with 3 × 15 mL of water. The organic layer was dried over anhydrous Na_2_SO_4_ and evaporated. The crude material was purified through column chromatography using DCM/MeOH (9:1) as eluent.

##### *N*-[3-(4-Allylpiperazin-1-yl)­propyl]­isoquinoline-1-carboxamide (**LINS05412**)

Reaction with **LINS05410** gave a yellowish oil. Yield: 24%. ^1^H NMR (300 MHz, CDCl_3_) δ: 9.59–9.48 (m, 1H), 9.00 (s, 1H), 8.47 (d, *J* = 5.5 Hz, 1H), 7.84 (dd, *J* = 7.2, 2.1 Hz, 1H), 7.78 (d, *J* = 5.5 Hz, 1H), 7.74–7.60 (m, 2H), 5.87 (ddt, *J* = 16.8, 10.1, 6.6 Hz, 1H), 5.26–5.09 (m, 2H), 3.61 (dd, *J* = 12.5, 6.3 Hz, 2H), 3.01 (d, *J* = 6.6 Hz, 2H), 2.78–2.34 (m, 10H), 1.85 (quint, *J* = 6.3 Hz, 2H). ^13^C NMR (75 MHz, CDCl_3_) δ: 166.2, 149.1, 140.2, 137.3, 134.8, 130.4, 128.4, 127.9, 126.9, 126.7, 124.0, 118.1, 61.8, 57.3, 53.2, 52.9, 39.2, 25.7. HRMS (ESI) *m*/*z*: [M + H]^+^ calcd.: 339.2179; [M + H]^+^ found: 339.2177. HPLC-UV: RT: 5.69 min (95%).

##### *N*-[3-(4-Allylpiperazin-1-yl)­propyl]­quinoline-2-carboxamide (**LINS05612**)

Reaction with **3a** furnished a yellowish oil. Yield: 70%. ^1^H NMR (300 MHz, CDCl_3_) δ: 8.77 (s, 1H), 8.36–8.26 (m, 2H), 8.17 (d, *J* = 8.4 Hz, 1H), 7.89 (dd, *J* = 8.2, 1.1 Hz, 1H), 7.77 (ddd, *J* = 8.4, 6.9, 1.1 Hz, 1H), 7.66–7.58 (m, 1H), 5.86 (ddt, *J* = 16.8, 10.1, 6.6 Hz, 1H), 5.25–5.06 (m, 2H), 3.63 (dd, *J* = 12.7, 6.5 Hz, 2H), 3.02 (d, *J* = 6.6 Hz, 2H), 2.69–1.94 (m, 10H), 1.87 (quint, *J* = 6.5 Hz, 2H). ^13^C NMR (75 MHz, CDCl_3_) δ: 164.6, 150.2, 146.5, 137.4, 135.1, 129.9, 129.8, 129.3, 127.8, 127.8, 119.1, 118.0, 61.8, 57.2, 53.3, 53.0, 38.9, 26.3. HRMS (ESI) *m*/*z*: [M + H]^+^ calcd.: 339.2179; [M + H]^+^ found: 339.2177. HPLC-UV: RT: 3.88 min (95%).

##### *N*-[5-(4-Allylpiperazin-1-yl)­pentyl]­quinoline-2-carboxamide (**LINS05632**)

Reaction with **3b** furnished a yellowish oil. Yield: 72%. ^1^H NMR (300 MHz, CDCl_3_) δ: 8.42–8.21 (m, 3H), 8.11 (d, *J* = 8.4 Hz, 1H), 7.88 (dd, *J* = 8.2, 0.8 Hz, 1H), 7.77 (ddd, *J* = 8.4, 6.9, 0.8 Hz, 1H), 7.67–7.56 (m, 1H), 5.86 (ddt, *J* = 16.8, 10.1, 6.6 Hz, 1H), 5.24–5.08 (m, 2H), 3.54 (dd, *J* = 13.5, 6.9 Hz, 2H), 3.00 (d, *J* = 6.6 Hz, 2H), 2.64–2.15 (m, 10H), 1.82–1.66 (m, 2H), 1.65–1.51 (m, 2H), 1.51–1.38 (m, 2H). ^13^C NMR (75 MHz, CDCl_3_) δ: 164.4, 149.9, 146.5, 137.5, 134.9, 130.1, 129.7, 129.3, 127.8, 127.8, 118.9, 118.1, 61.7, 58.5, 53.1, 52.9, 39.5, 29.7, 26.5, 25.0. HRMS (ESI) *m*/*z*: [M + H]^+^ calcd.: 367.2492; [M + H]^+^ found: 367.2499. HPLC-UV: RT: 9.19 min (95%).

### Radioligand Displacement Assay

Binding affinities toward H_3_R were determined via radioligand displacement assay on membrane fractions of HEK-293-hH_3_R cells, as previously reported.^26,51^ In brief, test compounds, [^3^H]­Nα-methylHA and membrane preparations were incubated for 90 min at 25 °C under constant shaking. Receptor bound ligands were collected on GF/B filters pretreated with 0.3% polytheylenimine and radioactivity was measured by liquid scintillation counting. Nonspecific binding was determined with pitolisant (10 μM). p*K*_i_ values were calculated from IC_50_ values using the Cheng–Prusoff equation, based on data from at least three independent experiments in duplicates and reported as means with SEM.

### Inhibition of Cholinesterases

The compounds (as hydrogenmaleate salts or free bases) were evaluated for their inhibitory activity on eeAChE and eqBChE in a 96-well microplate following the Ellman’s method as described by our group.^24,25^ The assay is based on the generation of thiocholine from the enzyme-catalyzed hydrolysis of acetylthiocholine iodide (ATCI), which reacts with 5,5′-dithiobis-2-nitrobenzoic acid (DTNB) to form the yellowish products detected in the spectrophotometer.^25,52^ The compounds were initially screened at a single concentration of 100 μM. The enzyme (eeAChE or eqBChE) was used at a concentration of 0.025 U/mL and DTNB was used at a concentration of 1.5 mM. Donepezil hydrochloride (2 μM) and neostigmine mesylate (100 μM) were used as pharmacological standards. The solutions were prepared in phosphate buffer (0.1 M—pH 7.5). The plates were incubated at 37 °C for 10 min and then an ATCI solution (at 1.5 mM) was added. The readings were monitored every 4 min over 30 min at 415 nm wavelength. For the most interesting compounds, full concentration response curves were performed. This data was used to estimate the concentration to reduce the activity by 50% (IC_50_) through nonlinear regression, and expressed as –logIC_50_ (pIC_50_ ± SEM). These data are presented in Table 1.

### Enzymatic Kinetic Studies

The protocol^25^ was performed with the same substrate from inhibition assays (ATCI), with concentrations varying from 0.25 to 4 mM for eeAChE and from 0.125 to 2 mM for eqBChE. The same concentrations to DTNB and enzymes were used. The study was performed for compounds **LINS05413** and **LINS05613** as eeAChE inhibitors, in concentrations varying from 6.75 to 100 μM and from 2.34 to 35.5 μM, respectively. The kinetic study at eqBChE was performed using the compounds **LINS05516** and **LINS05613** as inhibitors, in concentrations varying from 0.94 to 15 μM and from 4.69 to 75 μM, respectively. The experiments were performed in triplicate. The *V*_maxapp_ and *K*_Mapp_ values of the Michaelis–Menten kinetics were estimated through nonlinear regression from substrate/velocity curves. Obtained data was used to build the Lineweaver–Burk plot by using linear regression.

### Metal-Chelating Activity

The metal-chelating activity was evaluated in a UV-transparent 96-well microplate assay, adapted from literature.^32,33^ The compounds (as free bases) were evaluated for their ability to absorb UV–visible radiation in the presence and absence of solutions of the metals in the form of soluble sulfates or chlorides (FeSO_4_, FeCl_3_ and CuSO_4_). The assay is based on the occurrence of changes in absorbance patterns from the ligand alone or in the presence of the metal, which indicate interactions occurring between ligand and metal.^53,54^ The assay was performed as follows: In each well, 100 μL of the compound solution at 100 μM were added, followed by the addition of another 100 μL of each metal ion studied (prepared at 100 μM). For the evaluation of the compounds or metals alone, 100 μL of MeOH were used to complete the volume of 200 μL in each well. The absorbance readings were performed in a wavelength range of 200 to 700 nm. The spectral changes obtained in the absorbance readings of the compound plus metal were evaluated through the difference of the spectra of the ligand and the metal alone.

### Efficiency Analysis

The LE and LLE values were calculated using the previously reported formulas from literature^35^

$$LE=\left(1.37\times pK_{i}\;\text{or}\;{pIC}_{50}\right)/nHA$$

$$LLE=pK_{i}\;\text{or}\;{pIC}_{50}-logP$$

where *n*HA is the number of heavy (non-hydrogen) atoms in the molecule. The logP values were calculated using the ChemAxon’s Marvin software (version 23.17) as the consensus value, which is an average of three different methods. The TPSA values were calculated in the SwissADME online platform,^55^ and the BBB permeation were estimated using the BOILEgg model implemented in the Web site (www.swissadme.ch).

### Molecular Docking

To investigate the relationships between affinity data and potential binding modes, molecular docking experiments were carried out. The three-dimensional experimental structures of the human targets H_3_R (PDB 7F61), AChE (PDB 6O4W) and BChE (PDB 9R3C) were downloaded from Protein Data Bank (PDB) in a.pdb file format. The complexed ligands and water molecules were removed. The clean protein structure was used in the molecular docking experiments using the DockThor Web server (https://dockthor.lncc.br) and GOLD (CCDC, version 2020.1) software.^56,57^ DockThor is a freely available Web server and well-stablished in literature. Dockthor uses genetic algorithm to predict multiple solutions and the scoring function of the Merck Molecular Force Field 94S (MMFF94S) to predict the binding poses.^57^ GOLD is a standalone software that also employs the genetic algorithm to predict the binding poses.^56^ The size of docking search’s grid boxes (*X*, *Y*, *Z* and respective center coordinates) were defined as follows: H_3_R—19 × 22 × 19 (−22, 50, 0); AChE—18 × 23 × 18 (88, 84, −5); BChE—20 × 19 × 20 (18, 42, 39). These search boxes included the key amino acid residues from each target. The default setup conditions were maintained for both softwares.

For methodology validation, redocking procedures were executed by extracting the ligands within the PDB crystal structure from each target. The experimental ligands were redocked on each respective target using the same molecular docking methods, which returned low RMSD values (<1.0 Å) for all cases. The analysis of the results was conducted using the Discovery Studio Visualizer (Dassault Systemes Biovia Corp, version 24.1.0.23298).

The three-dimensional structures of the tested ligands were build using the Marvin Sketch software (Chemaxon Inc., version 23.17.0) on their main ionized forms as predicted by the p*K*_a_ add-on implemented in the software and saved as.mol2 files. The top ten docking poses were ranked by the implemented scores in each software (DockTScore and GoldScore scoring functions) and visually assessed on Discovery Studio Visualizer for reasonable binding modes. The ligand–target interactions were predicted using the same software. Since both softwares returned similar binding poses, a consensus binding mode with highest score was used in the analysis.

## Supplementary Material



## Notes

The copies of ^1^H and ^13^C NMR spectra, HRMS and additional UV–vis absorption spectra (PDF)

## Abbreviations

ACh: acetylcholine
AChE: acetylcholinesterase
AD: Alzheimer disease
ADMET: absorption, distribution, metabolism, excretion and toxicity
ALS: amyotrophic lateral sclerosis
ATCI: acetylthiocholine iodide
BBB: blood–brain barrier
BChE: butyrylcholinesterase
CAS: catalytic anionic site
ChEs: cholinesterases
CNS: central nervous system
DCM: dichloromethane
DTNB: 5,5′-dithiobis-2-nitrobenzoic acid
EDC: *N*-ethyl-*N*′-(3-(dimethylamino)­propyl)­carbodiimide
ESI: electron-spray ionization
H_3_R: histamine H_3_ receptor
HA: histamine
HOBt: 1-hydroxybenzotriazole
HPLC: high performance liquid chromatography
HRMS: high resolution mass spectra
LE: ligand efficiency
LLE: lipophilic ligand efficiency
mAChR: muscarinic cholinergic receptor
MC: metal chelating
MMFF94S: Merck Molecular Force Field 94S
nAChR: nicotinic cholinergic receptor
NMR: nuclear magnetic resonance
PAS: peripheral anionic site
PD: Parkinson disease
PDB: Protein Data Bank
PWS: Prader–Willi syndrome
RMSD: root-mean-square deviation
ROS: reactive oxygen species
SARM: structure activity relationship matrix
SEM: standard error mean
TEA: triethylamine
TFA: trifluoroacetic acid
THF: tetrahydrofuran
TLC: thin layer chromatography
TMS: tetramethylsilane
TPSA: topological polar surface area
