# Supplementary material for: Multitargeted Aza-Arylcarboxamides for Neurodegenerative Diseases: Potent Histamine H3 Receptor Ligands with Anticholinesterase and Metal-Chelating Activities
Source: ACS Chem Neurosci. 2026 Jan 29;17(5):998–1014. doi: 10.1021/acschemneuro.5c00803 (PMC12964414; doi:10.1021/acschemneuro.5c00803)
Supplement: Supplementary file 1 [file cn5c00803_si_001.pdf]

Multitargeted Agents for Neurodegenerative Diseases: Potent Histamine H_3 Receptor Ligands with Anticholinesterase and Metal-Chelating Activities

Flavia B. Lopes[1,2], Tobias Werner[3], Izilda A. Bagatin[4], Holger Stark[3] and João Paulo S. Fernandes[1]**

[1]Department of Pharmaceutical Sciences, Federal University of São Paulo, 09913-030 Diadema-SP, Brazil

[2]Department of Medicine, Federal University of São Paulo, 04023-062 São Paulo-SP, Brazil

[3] Institute of Pharmaceutical and Medicinal Chemistry, Heinrich Heine University Düsseldorf, 40225 Duesseldorf, Germany

[4]Department of Chemistry, Federal University of São Paulo, 04023-062 São Paulo-SP, Brazil

SUPPORTING INFORMATION

Copies of the NMR spectra and HRMS of the final compounds S2-S49

UV-Vis absorption spectra of selected compounds S50

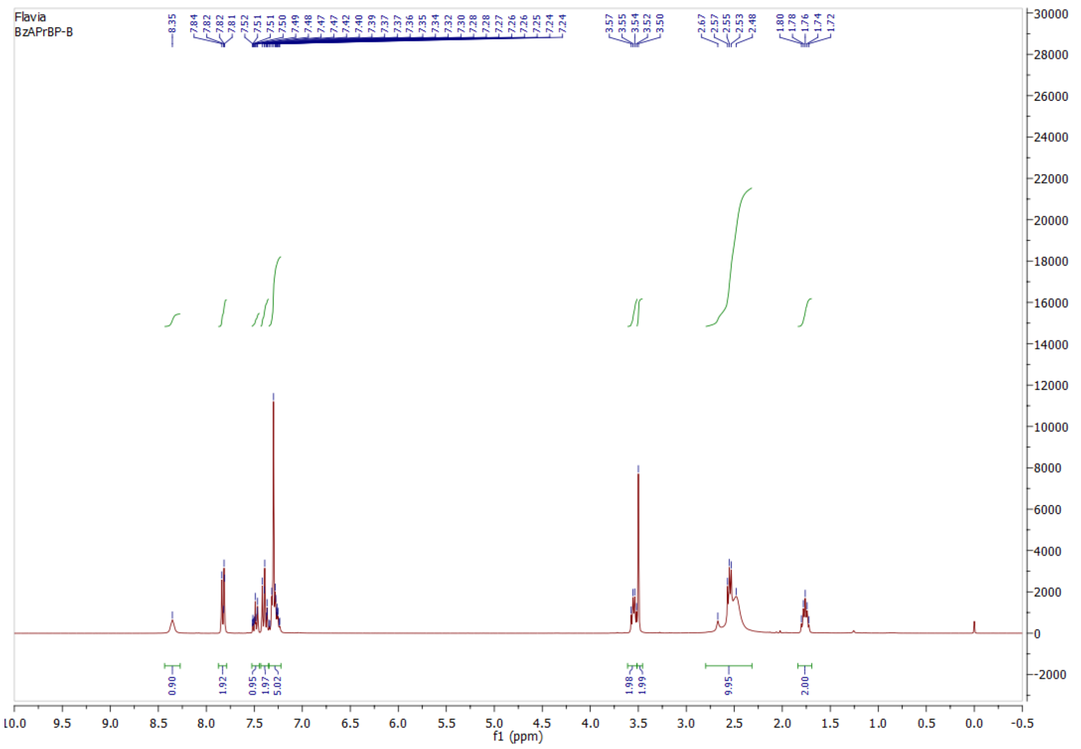

Figure S1. *N*-[3-(4-Benzylpiperazin-1-yl)propyl]benzamide (**LINS05113**). ^{1}H NMR (300 MHz, $CDCl_3$) δ 8.35 (s, 1H), 7.86 – 7.78 (m, 2H), 7.54 – 7.45 (m, 1H), 7.44 – 7.34 (m, 2H), 7.34 – 7.20 (m, 5H), 3.55 (dd, J = 11.8, 6.1 Hz, 2H), 3.50 (s, 2H), 2.64 – 2.29 (m, 10H), 1.76 (quint, J = 6.1 Hz, 2H).

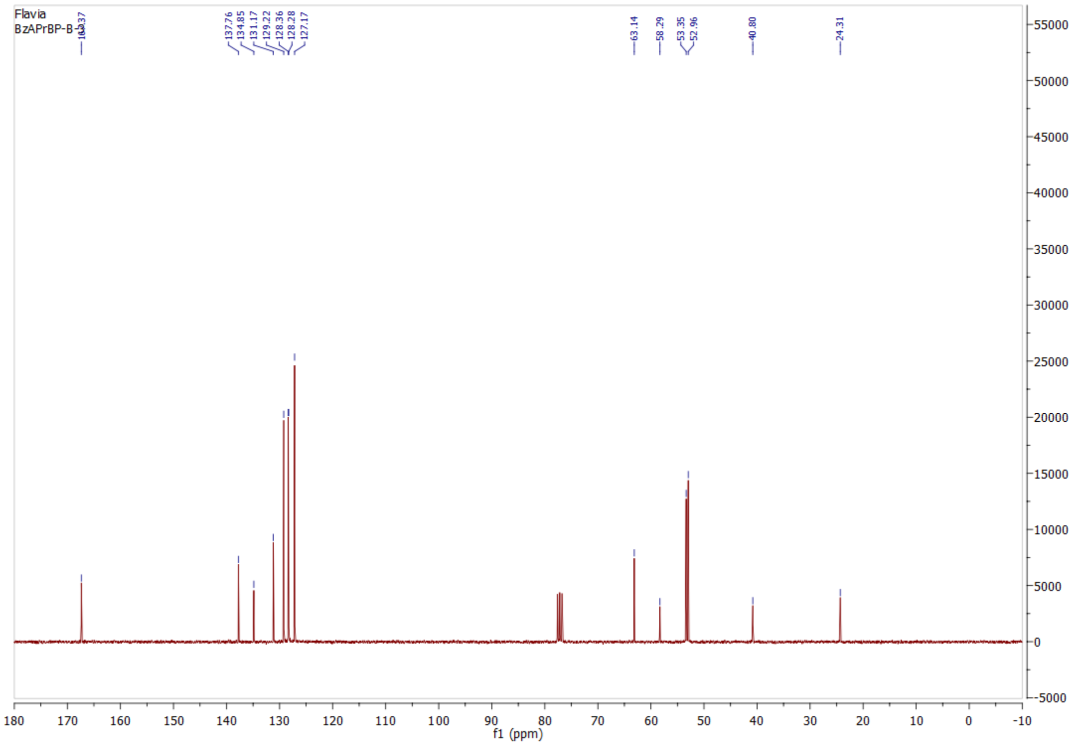

Figure S2. *N*-[3-(4-Benzylpiperazin-1-yl)propyl]benzamide (**LINS05113**). ^{13}C NMR (75 MHz, $CDCl_3$) δ 167.4, 137.8, 134.8, 131.2, 129.2, 128.4, 128.4, 128.3, 127.2, 63.1, 58.3, 53.4, 52.9, 40.8, 24.3.

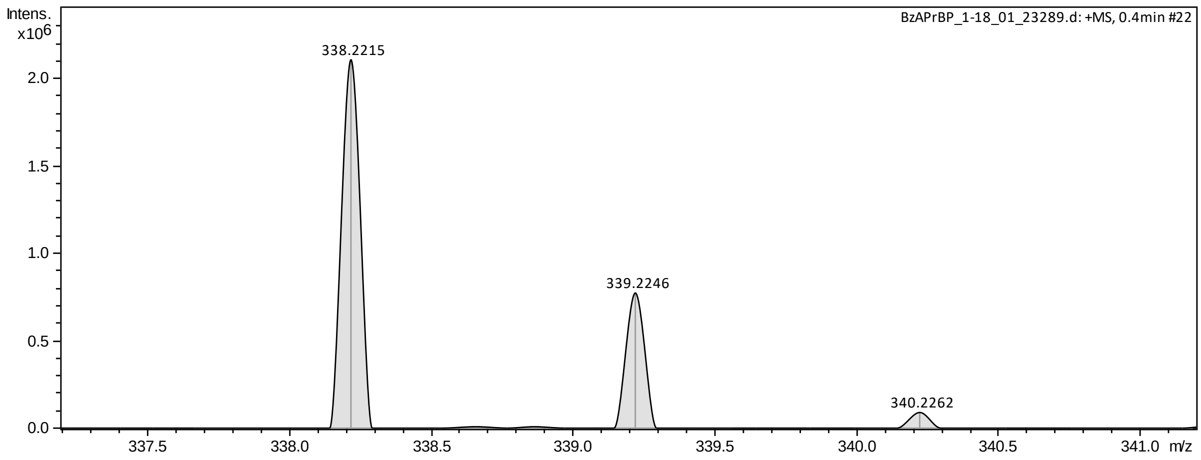

Figure S3. *N*-[3-(4-Benzylpiperazin-1-yl)propyl]benzamide (**LINS05113**). HRMS (ESI) *m/z*: $[M+H]^+$ calcd.: 338.2227; $[M+H]^+$ found: 338.2215.

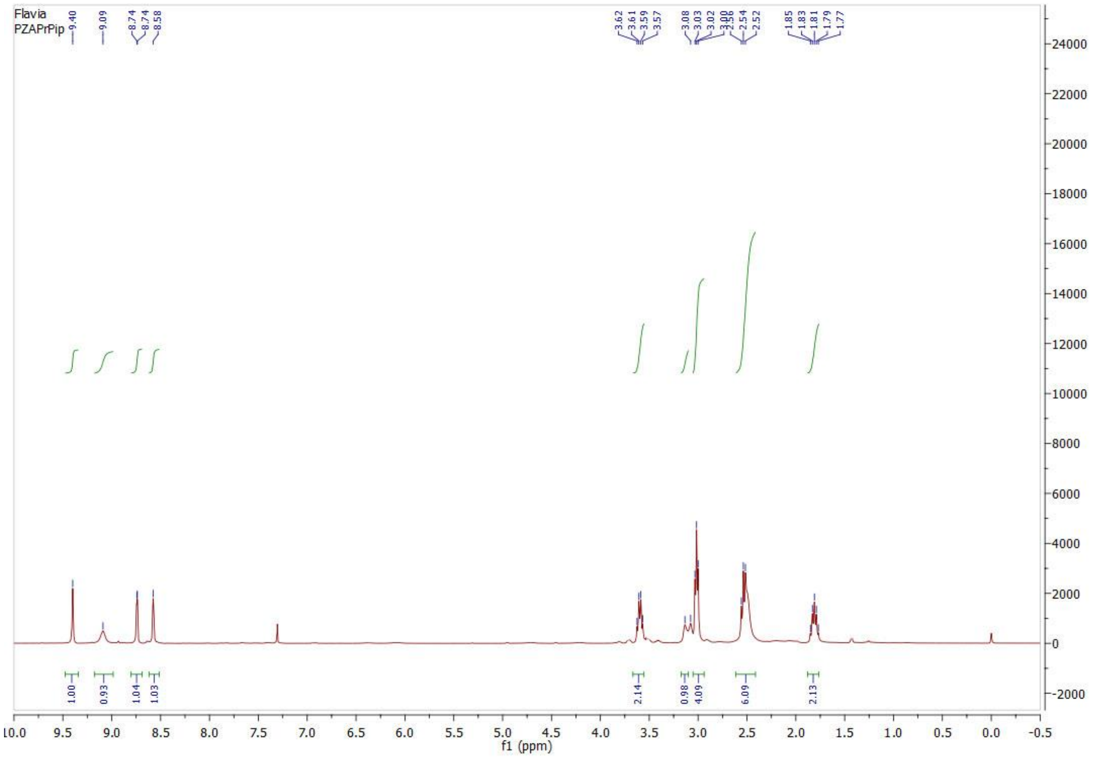

Figure S4. *N*-(3-Piperazin-1-ylpropyl)pyrazine-2-carboxamide (**LINS05210**). ^{1}H NMR (300 MHz, $CDCl_3$) δ 9.40 (s, 1H), 9.09 (s, 1H), 8.74 (d, J = 1.5 Hz, 1H), 8.58 (s, 1H), 3.60 (dd, J = 11.4, 5.6 Hz, 2H), 3.18 – 3.05 (m, 1H), 3.05 – 2.95 (m, 4H), 2.66 – 2.30 (m, 6H), 1.81 (quint, J = 5.6, 2H).

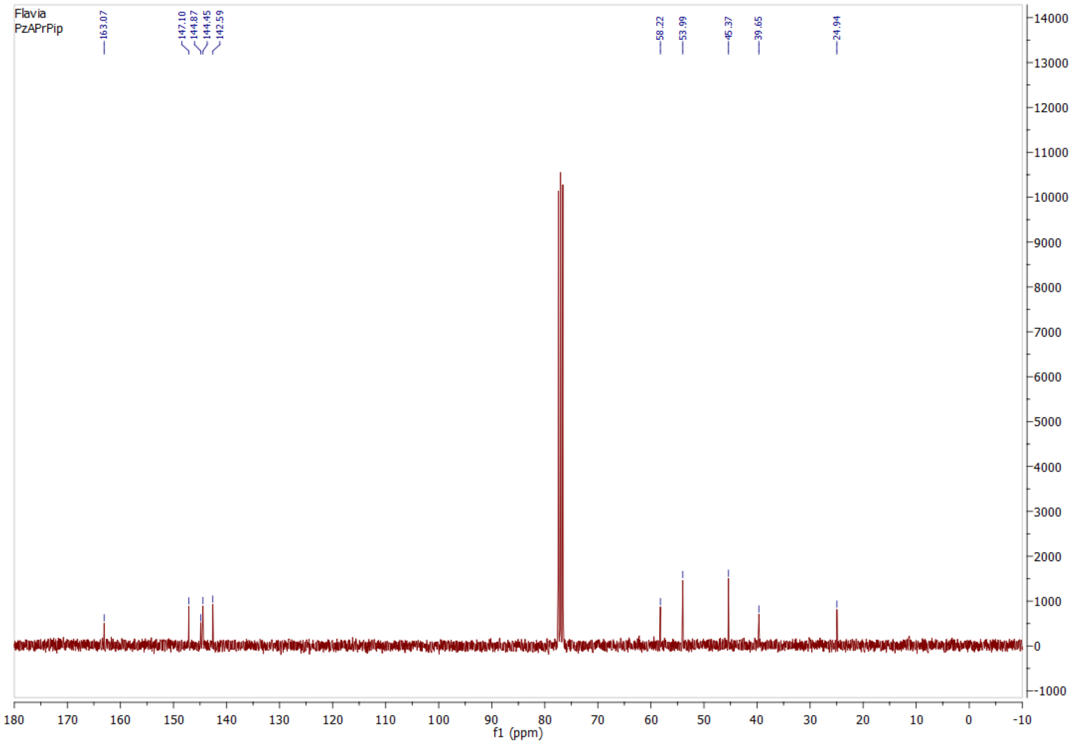

Figure S5. *N*-(3-Piperazin-1-ylpropyl)pyrazine-2-carboxamide (**LINS05210**). ^{13}C NMR (75 MHz, $CDCl_3$) δ 163.1, 147.1, 144.9, 144.4, 142.6, 58.2, 54.0, 45.4, 39.6, 24.9.

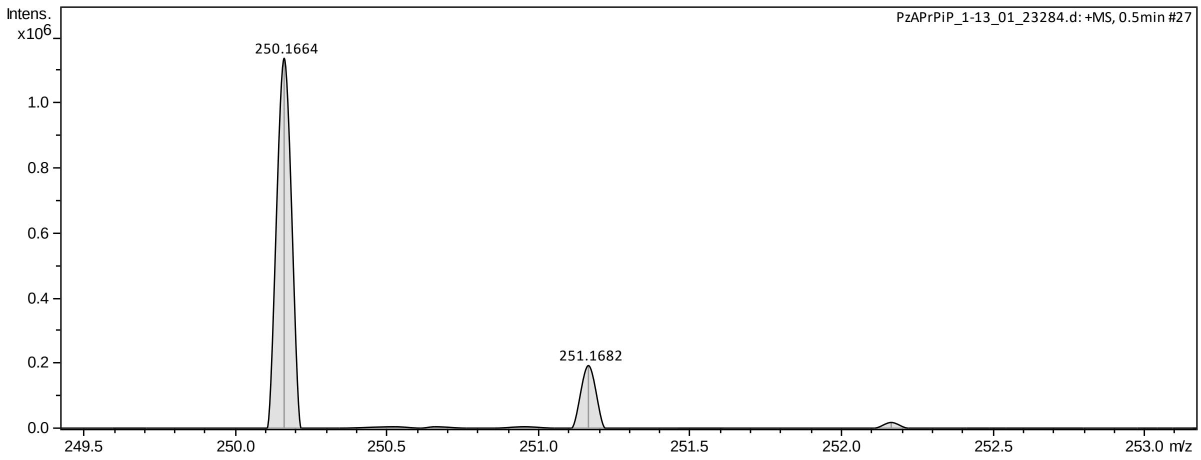

Figure S6. *N*-(3-Piperazin-1-ylpropyl)pyrazine-2-carboxamide (**LINS05210**). HRMS (ESI) *m/z*: $[M+H]^+$ calcd.: 250.1662; $[M+H]^+$ found: 250.1664.

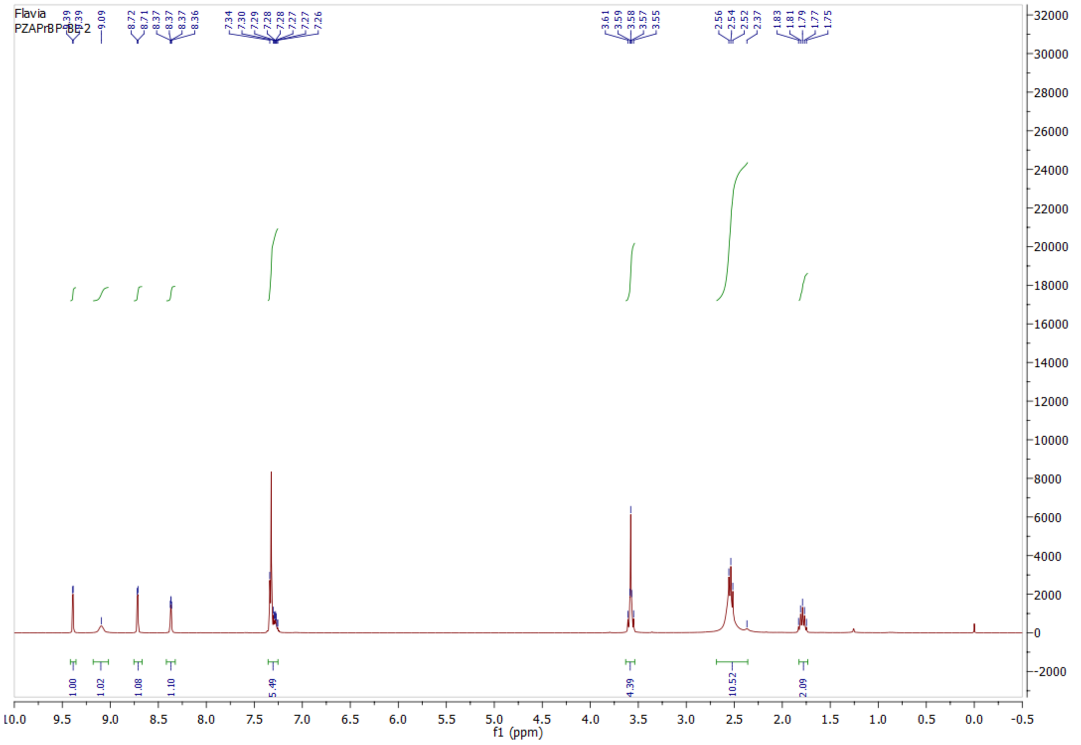

Figure S7. *N*-[3-(4-Benzylpiperazin-1-yl)propyl]pyrazine-2-carboxamide (**LINS05213**). ^{1}H NMR (300 MHz, $CDCl_3$) δ 9.39 (d, J = 1.5 Hz, 1H), 9.09 (s, 1H), 8.72 (d, J = 2.4 Hz, 1H), 8.37 (dd, J = 2.4, 1.5 Hz, 1H), 7.38 – 7.23 (m, 5H), 3.64 – 3.51 (m, 4H), 2.69 – 2.40 (m, 10H), 1.79 (quint, J = 6.1 Hz, 2H).

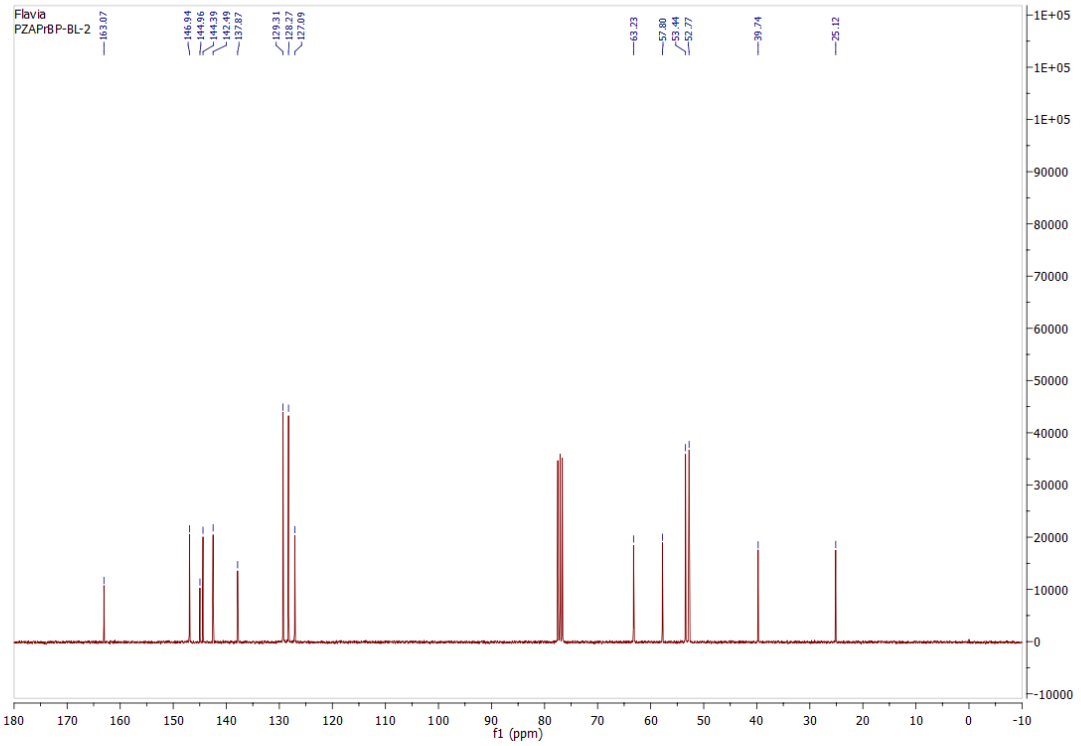

Figure S8. *N*-[3-(4-Benzylpiperazin-1-yl)propyl]pyrazine-2-carboxamide (**LINS05213**). ^{13}C NMR (75 MHz, $CDCl_3$) δ 163.1, 146.9, 144.9, 144.4, 142.5, 137.9, 129.3, 128.3, 127.1, 63.2, 57.8, 53.4, 52.8, 39.7, 25.1.

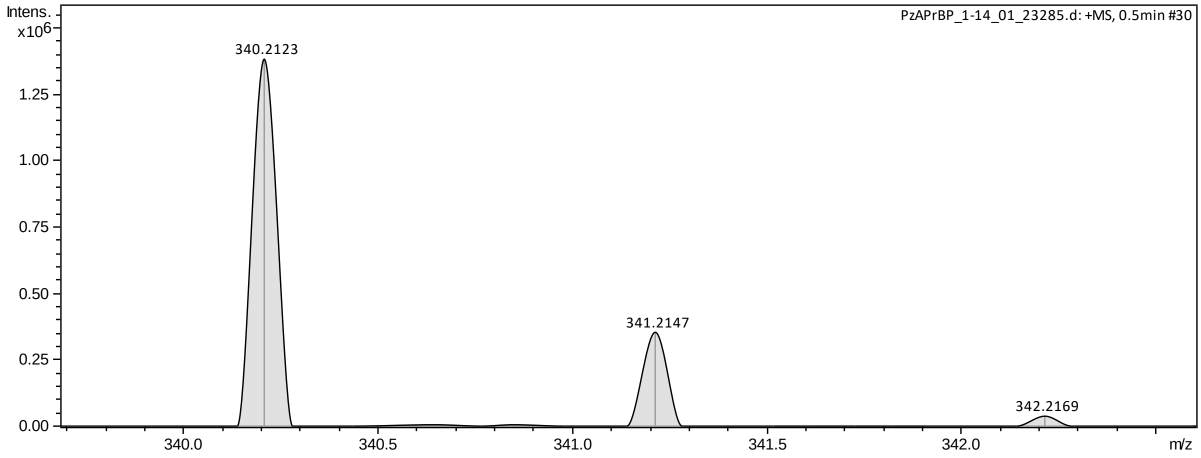

Figure S9. *N*-[3-(4-Benzylpiperazin-1-yl)propyl]pyrazine-2-carboxamide (**LINS05213**). HRMS (ESI) *m/z*: $[M+H]^+$ calcd.: 340.2131; $[M+H]^+$ found: 340.2123.

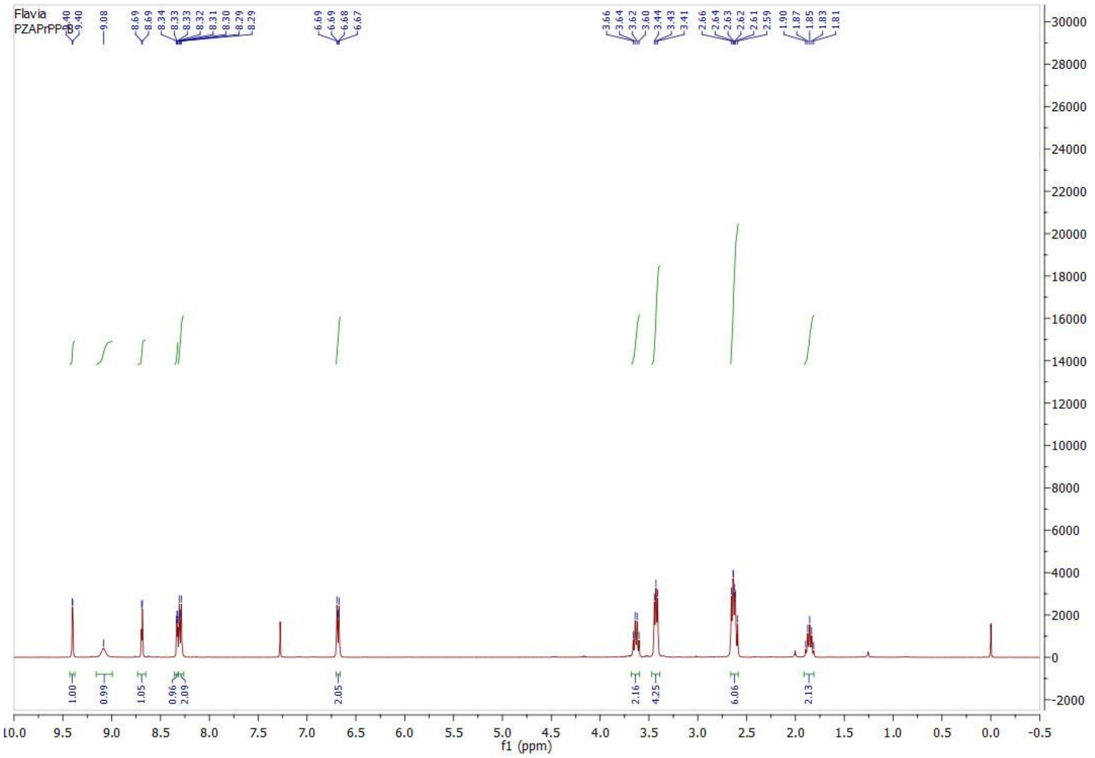

Figure S10. *N*-[3-[4-(4-Pyridyl)piperazin-1-yl]propyl]benzamide (**LINS05214**). ^{1}H NMR (300 MHz, $CDCl_3$) δ 9.40 (d, J = 1.4 Hz, 1H), 9.08 (s, 1H), 8.69 (d, J = 2.4 Hz, 1H), 8.33 (dd, J = 2.4, 1.4 Hz, 1H), 8.30 (dd, J = 5.0, 1.5 Hz, 2H), 6.68 (dd, J = 5.0, 1.5 Hz, 2H), 3.63 (dd, J = 11.8, 6.2 Hz, 2H), 3.50 – 3.34 (m, 4H), 2.71 – 2.55 (m, 6H), 1.85 (quint, J = 6.2 Hz, 2H).

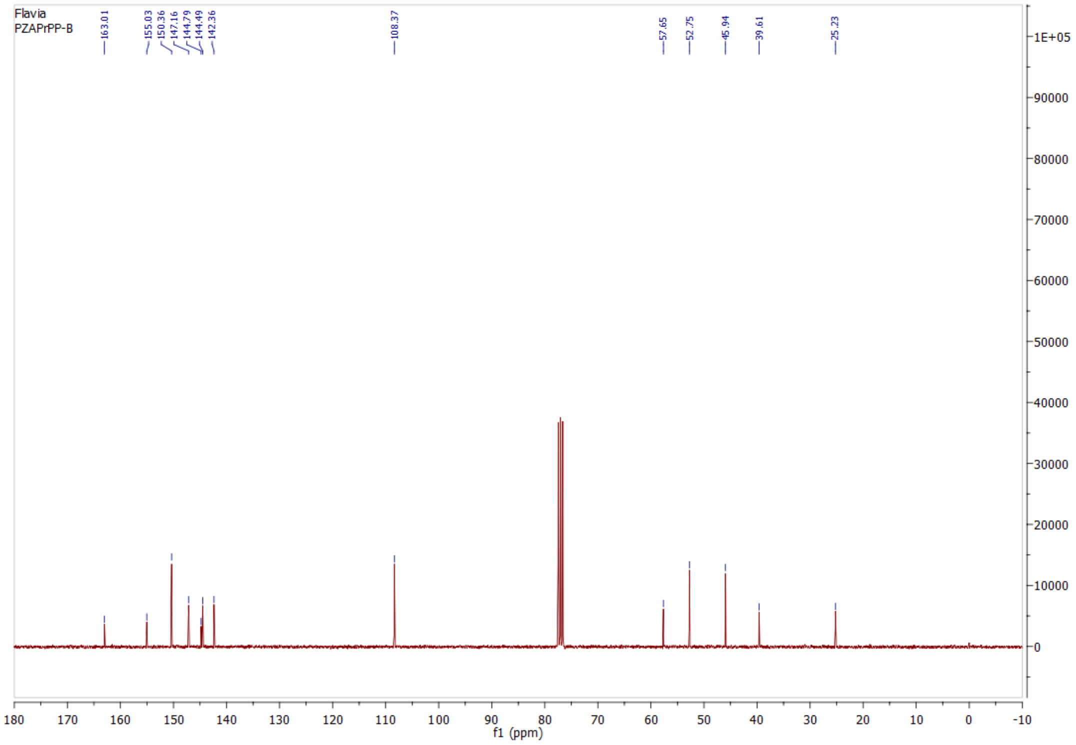

Figure S11. *N*-[3-[4-(4-Pyridyl)piperazin-1-yl]propyl]benzamide (**LINS05214**). ^{13}C NMR (75 MHz, $CDCl_3$) δ 163.0, 155.0, 150.4, 147.2, 144.8, 144.5, 142.4, 108.4, 57.6, 52.8, 45.9, 39.6, 25.2.

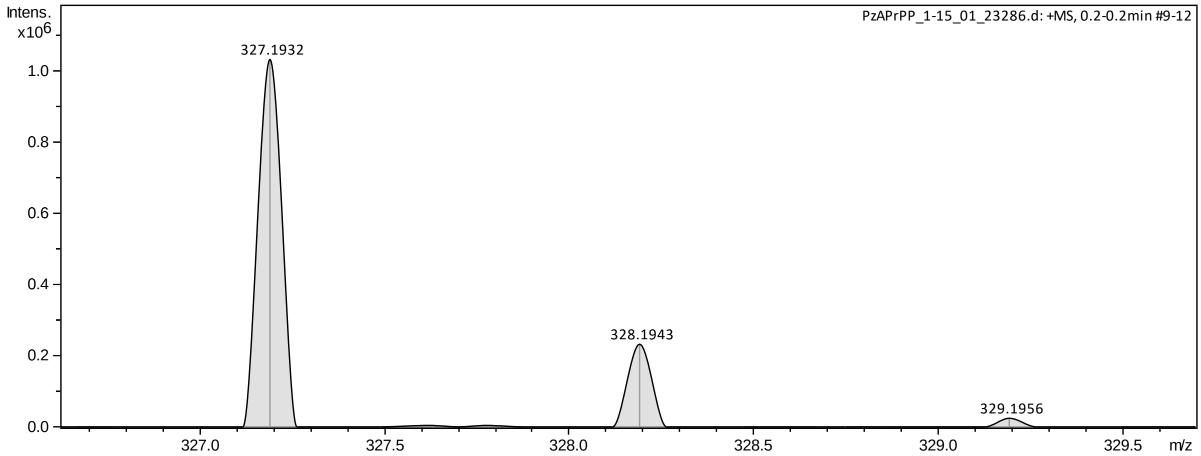

Figure S12. *N*-[3-[4-(4-Pyridyl)piperazin-1-yl]propyl]benzamide (**LINS05214**). HRMS (ESI) *m/z*: $[M+H]^+$ calcd.: 327.1927; $[M+H]^+$ found: 327.1932.

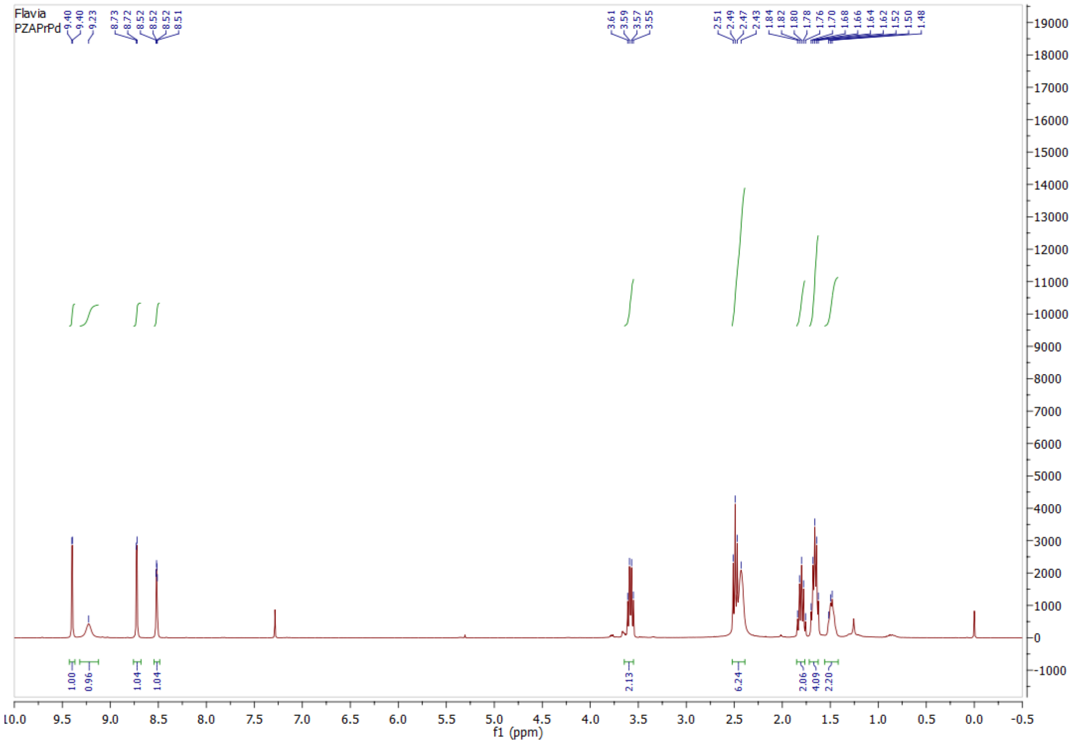

Figure S13. *N*-[3-(1-Piperidyl)propyl]pyrazine-2-carboxamide (**LINS05215**). ^{1}H NMR (300 MHz, $CDCl_3$) δ 9.40 (d, J = 1.4 Hz, 1H), 9.23 (s, 1H), 8.73 (d, J = 2.4 Hz, 1H), 8.52 (dd, J = 2.4, 1.4 Hz, 1H), 3.58 (dd, J = 12.2, 5.5 Hz, 2H), 2.62 – 2.28 (m, 6H), 1.80 (quint, J = 5.5 Hz, 2H), 1.73 – 1.59 (m, 4H), 1.58 – 1.38 (m, 2H).

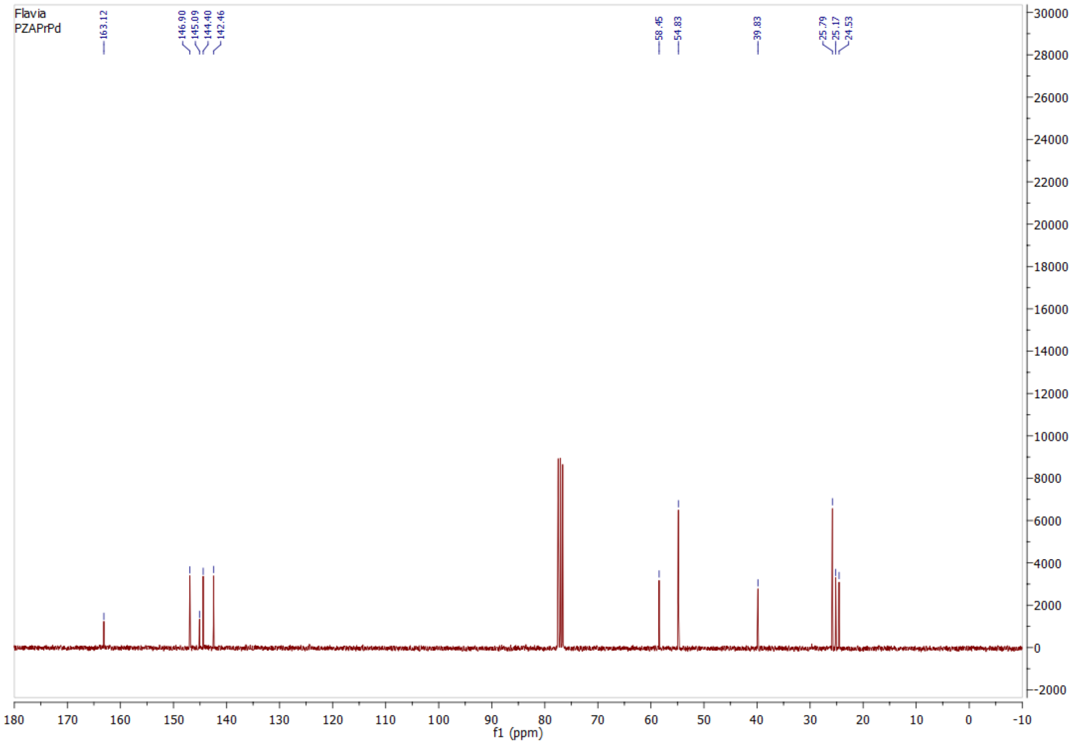

Figure S14. *N*-[3-(1-Piperidyl)propyl]pyrazine-2-carboxamide (**LINS05215**). ^{13}C NMR (75 MHz, $CDCl_3$) δ 163.1, 146.9, 145.1, 144.4, 142.5, 58.4, 54.8, 39.8, 25.8, 25.2, 24.5.

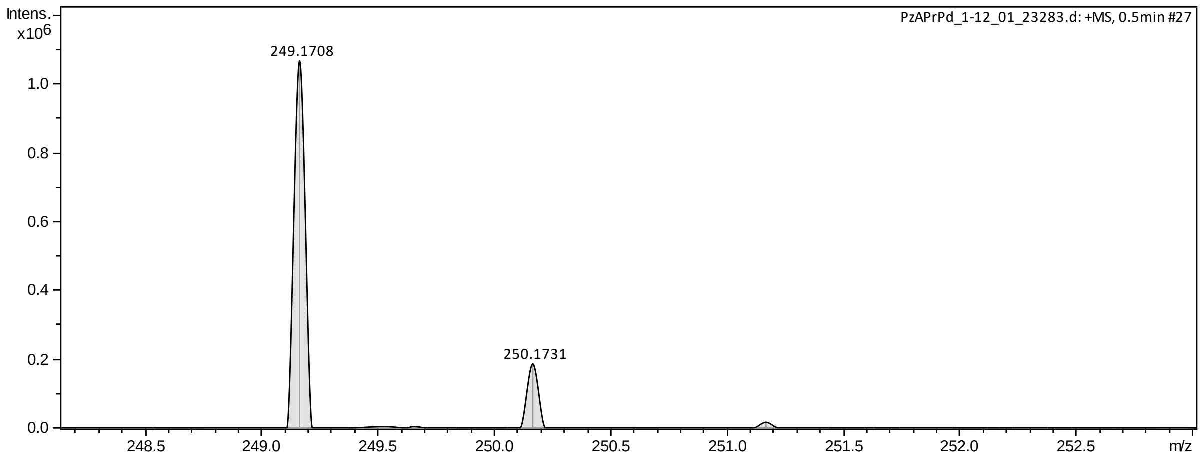

Figure S15. *N*-[3-(1-Piperidyl)propyl]pyrazine-2-carboxamide (**LINS05215**). HRMS (ESI) *m/z*: $[M+H]^+$ calcd.: 249.1709; $[M+H]^+$ found: 249.1708.

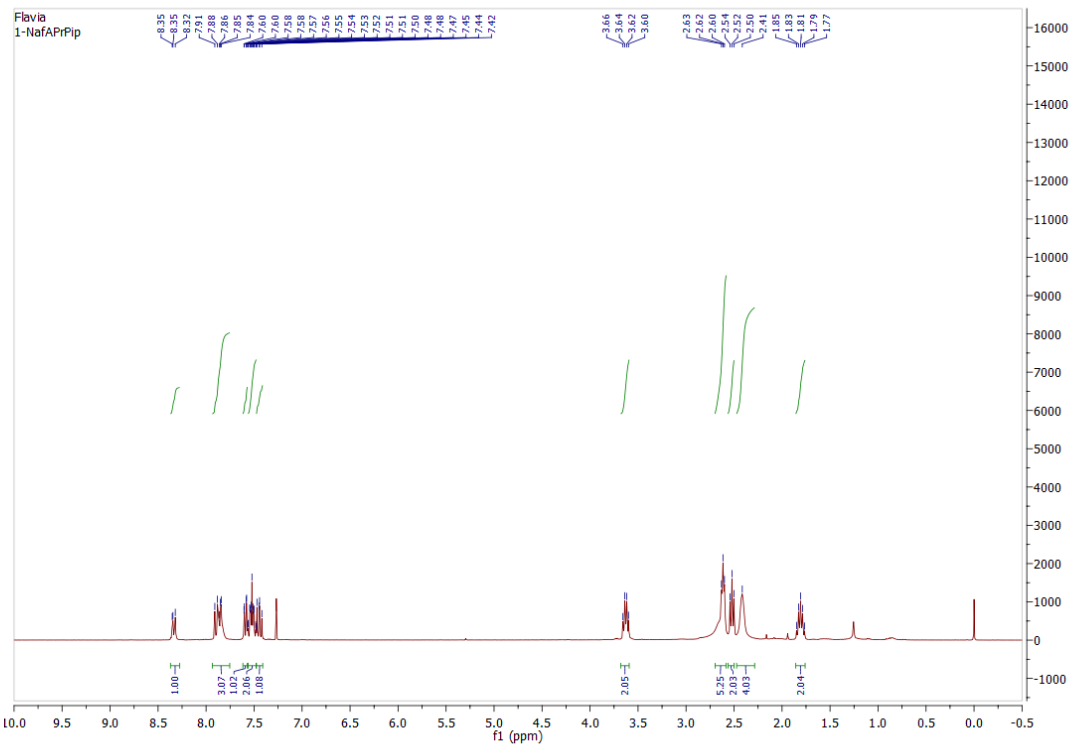

Figure S16. *N*-(3-Piperazin-1-ylpropyl)naphthalene-1-carboxamide (**LINS05310**). ^{1}H NMR (300 MHz, $CDCl_3$) δ 8.37 – 8.30 (m, 1H), 7.92 – 7.83 (m, 3H), 7.59 (dd, J = 7.0, 1.1 Hz, 1H), 7.57 – 7.47 (m, 2H), 7.44 (dd, J = 8.1, 1.1 Hz, 1H), 3.63 (dd, J = 11.7, 6.2 Hz, 2H), 2.76 – 2.57 (m, 5H), 2.52 (t, J = 6.2 Hz, 2H), 2.47 – 2.32 (m, 4H), 1.81 (quint, J = 6.2 Hz, 2H).

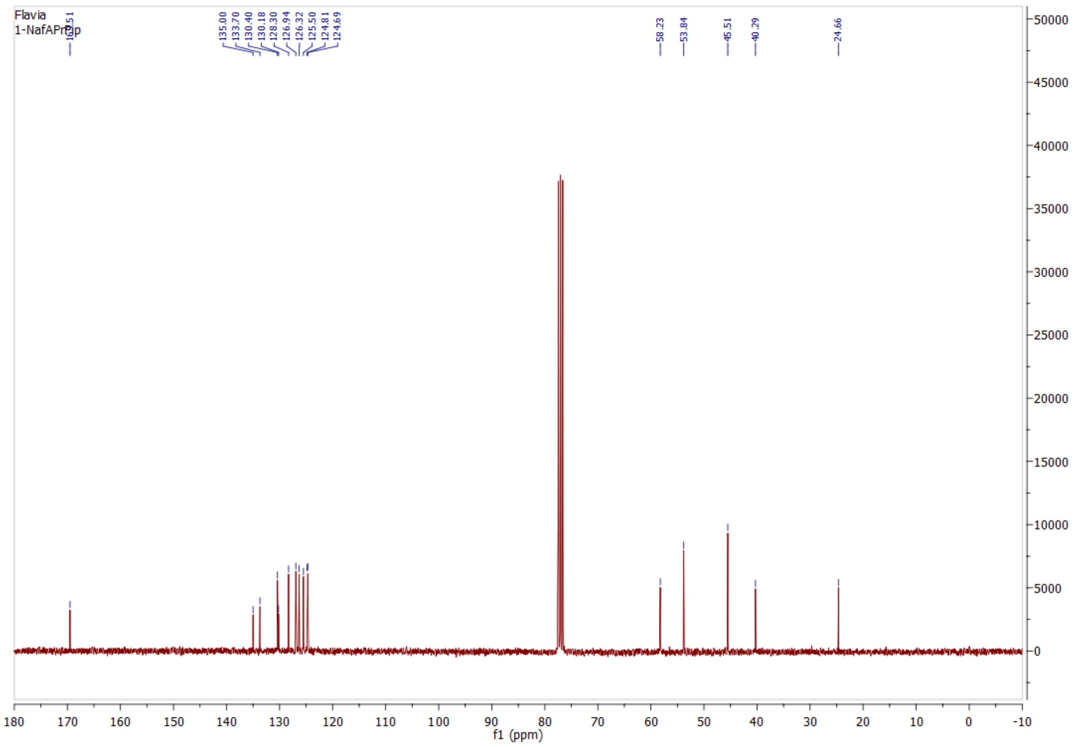

Figure S17. *N*-(3-Piperazin-1-ylpropyl)naphthalene-1-carboxamide (**LINS05310**). ^{13}C NMR (75 MHz, $CDCl_3$) δ 169.5, 135.0, 133.7, 130.4, 130.2, 128.3, 126.9, 126.3, 125.5, 124.8, 124.7, 58.2, 53.8, 45.5, 40.3, 24.7.

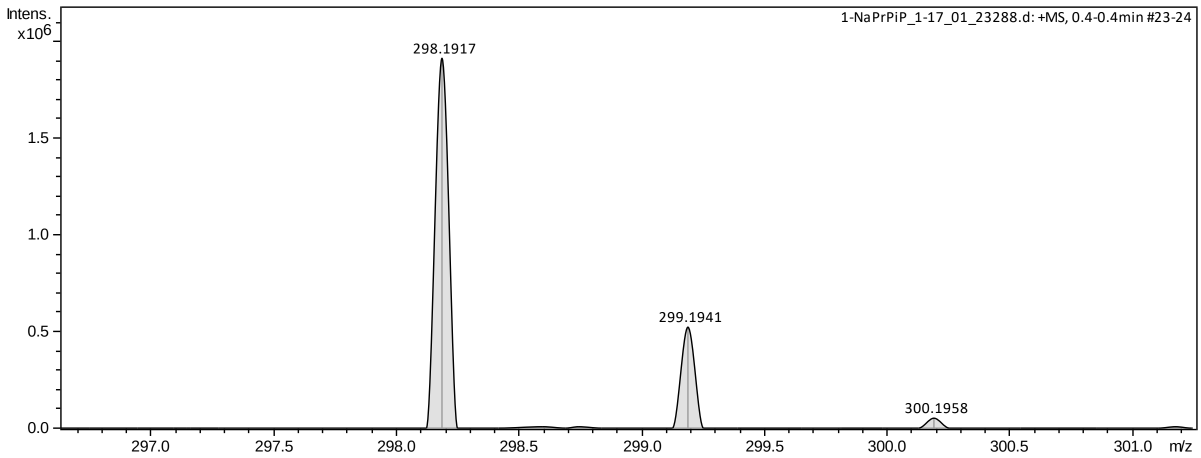

Figure S18. *N*-(3-Piperazin-1-ylpropyl)naphthalene-1-carboxamide (**LINS05310**). HRMS (ESI) *m/z*: $[M+H]^+$ calcd.: 298.1913; $[M+H]^+$ found: 298.1917.

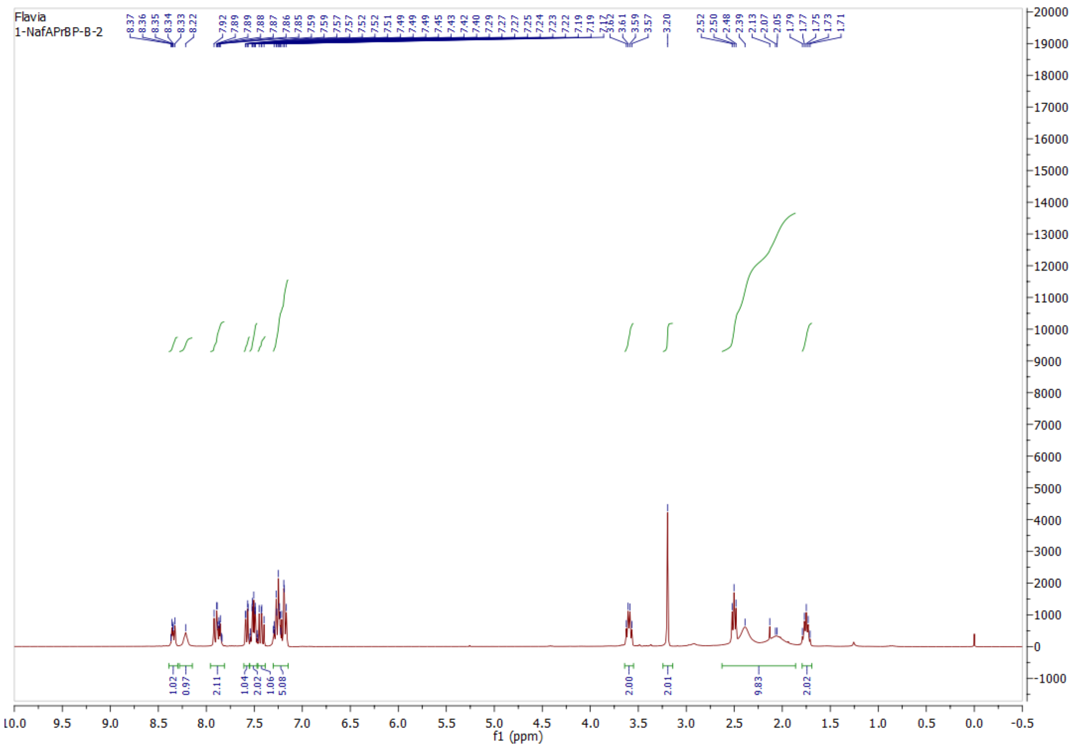

Figure S19. *N*-[3-(4-Benzylpiperazin-1-yl)propyl]naphthalene-1-carboxamide (**LINS05313**). ^{1}H NMR (300 MHz, $CDCl_3$) δ 8.38 – 8.30 (m, 1H), 8.22 (s, 1H), 7.97 – 7.81 (m, 2H), 7.60 – 7.54 (m, 1H), 7.55 – 7.46 (m, 2H), 7.47 – 7.39 (m, 1H), 7.35 – 7.13 (m, 5H), 3.60 (dd, J = 11.4, 5.9 Hz, 2H), 3.20 (s, 2H), 2.72 – 1.85 (m, 10H), 1.75 (quint, J = 5.9 Hz, 2H).

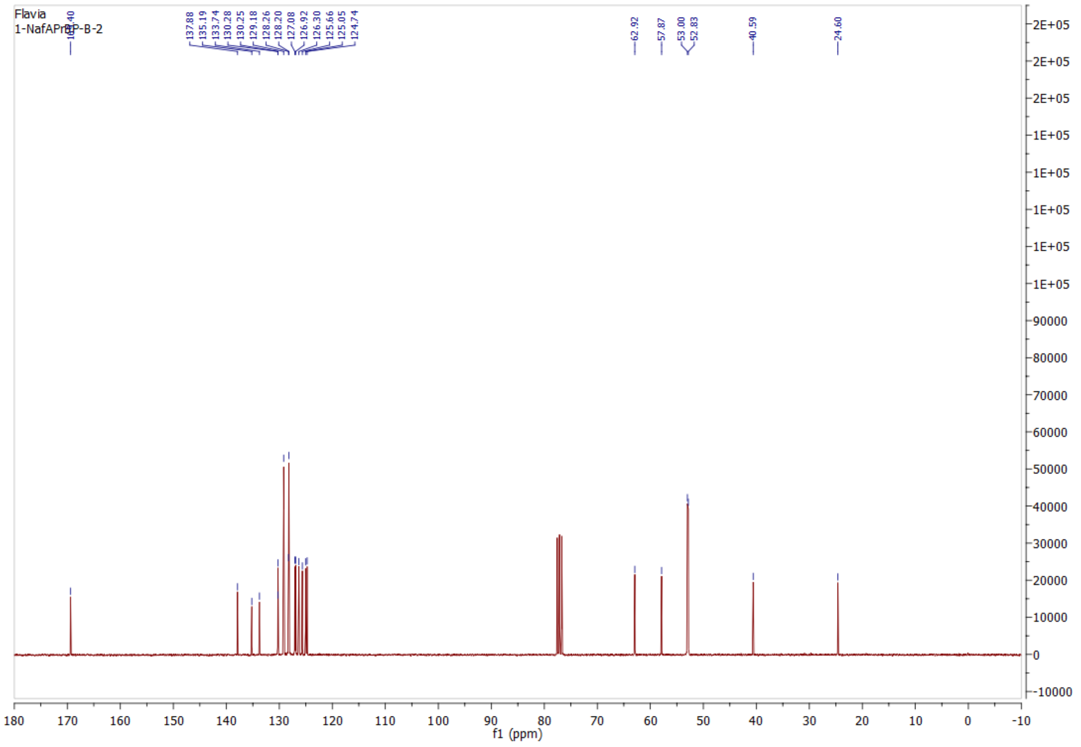

Figure S20. *N*-[3-(4-Benzylpiperazin-1-yl)propyl]naphthalene-1-carboxamide (**LINS05313**). ^{13}C NMR (75 MHz, $CDCl_3$) δ 169.4, 137.9, 135.2, 133.7, 130.3, 130.2, 129.2, 128.3, 128.2, 127.1, 126.9, 126.3, 125.7, 125.1, 124.7, 62.9, 57.9, 53.0, 52.8, 40.6, 24.6.

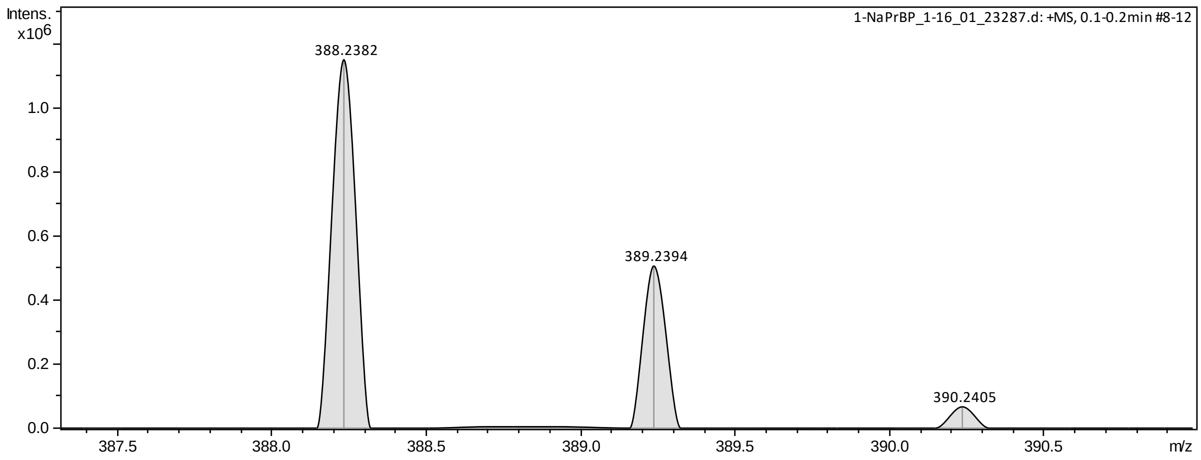

Figure S21. *N*-[3-(4-Benzylpiperazin-1-yl)propyl]naphthalene-1-carboxamide (**LINS05313**). HRMS (ESI) *m/z*: $[M+H]^+$ calcd.: 388.2383; $[M+H]^+$ found: 388.2382.

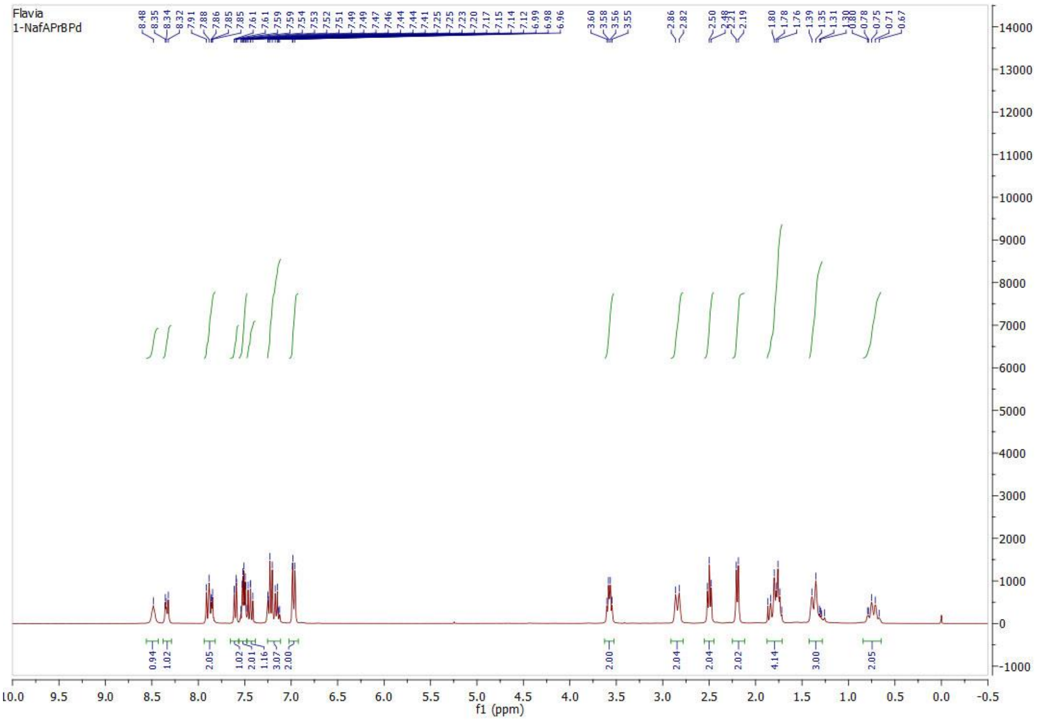

Figure S22. *N*-[3-(4-Benzyl-1-piperidyl)propyl]naphthalene-1-carboxamide (**LINS05316**). ^{1}H NMR (300 MHz, $CDCl_3$) δ 8.48 (s, 1H), 8.40 – 8.28 (m, 1H), 7.96 – 7.80 (m, 2H), 7.60 (dd, J = 7.0, 0.9 Hz, 1H), 7.55 – 7.47 (m, 2H), 7.44 (dd, J = 8.0, 7.0 Hz, 1H), 7.28 – 7.11 (m, 3H), 7.03 – 6.91 (m, 2H), 3.57 (dd, J = 11.2, 5.7 Hz, 2H), 2.84 (d, J = 11.6 Hz, 2H), 2.50 (t, J = 5.7 Hz, 2H), 2.20 (d, J = 6.7 Hz, 2H), 1.89 – 1.69 (m, 4H), 1.44 – 1.25 (m, 3H), 0.83 – 0.63 (m, 2H).

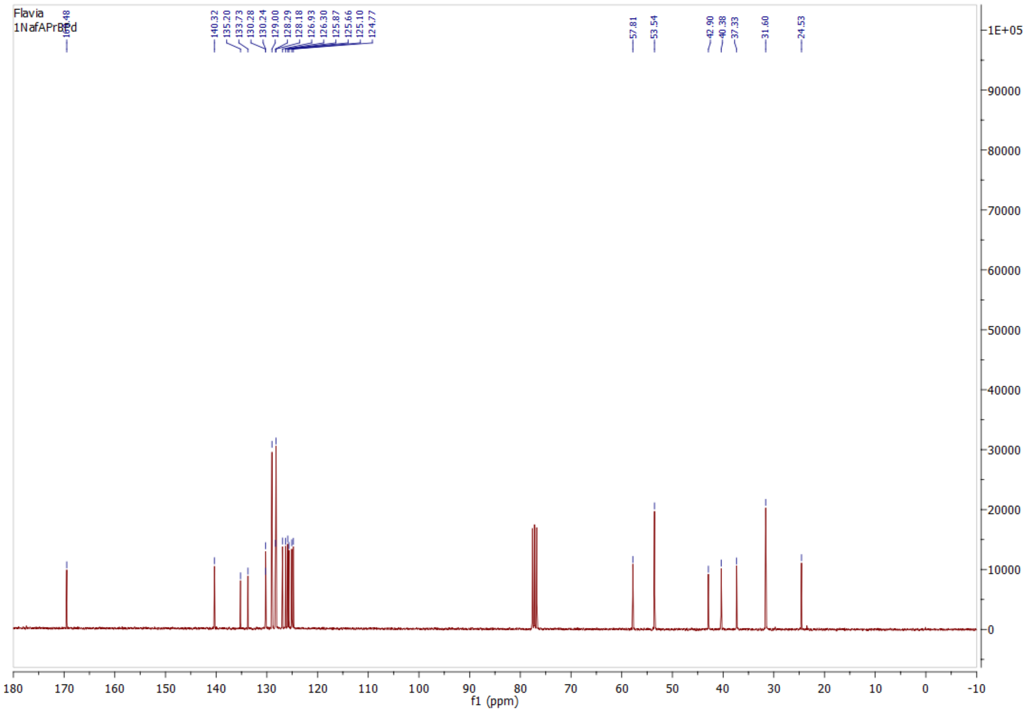

Figure S23. *N*-[3-(4-Benzyl-1-piperidyl)propyl]naphthalene-1-carboxamide (**LINS05316**). ^{13}C NMR (75 MHz, $CDCl_3$) δ 169.5, 140.3, 135.2, 133.7, 130.3, 130.2, 129.0, 128.3, 128.2, 126.9, 126.3, 125.9, 125.7, 125.1, 124.8, 57.8, 53.5, 42.9, 40.4, 37.3, 31.6, 24.5.

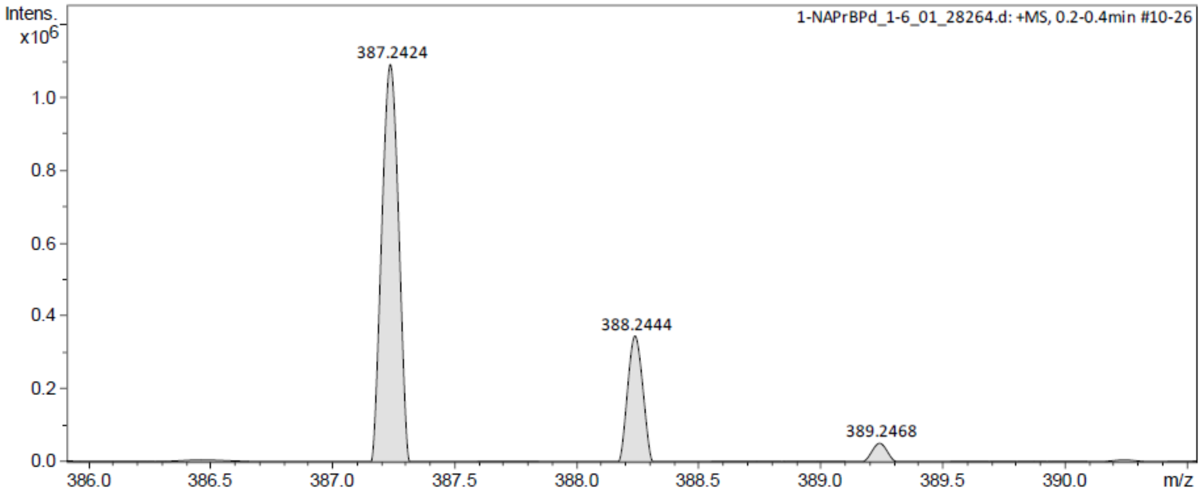

Figure S24. *N*-[3-(4-Benzyl-1-piperidyl)propyl]naphthalene-1-carboxamide (**LINS05316**). HRMS (ESI) *m/z*: $[M+H]^+$ calcd.: 387.2430; $[M+H]^+$ found: 387.2424.

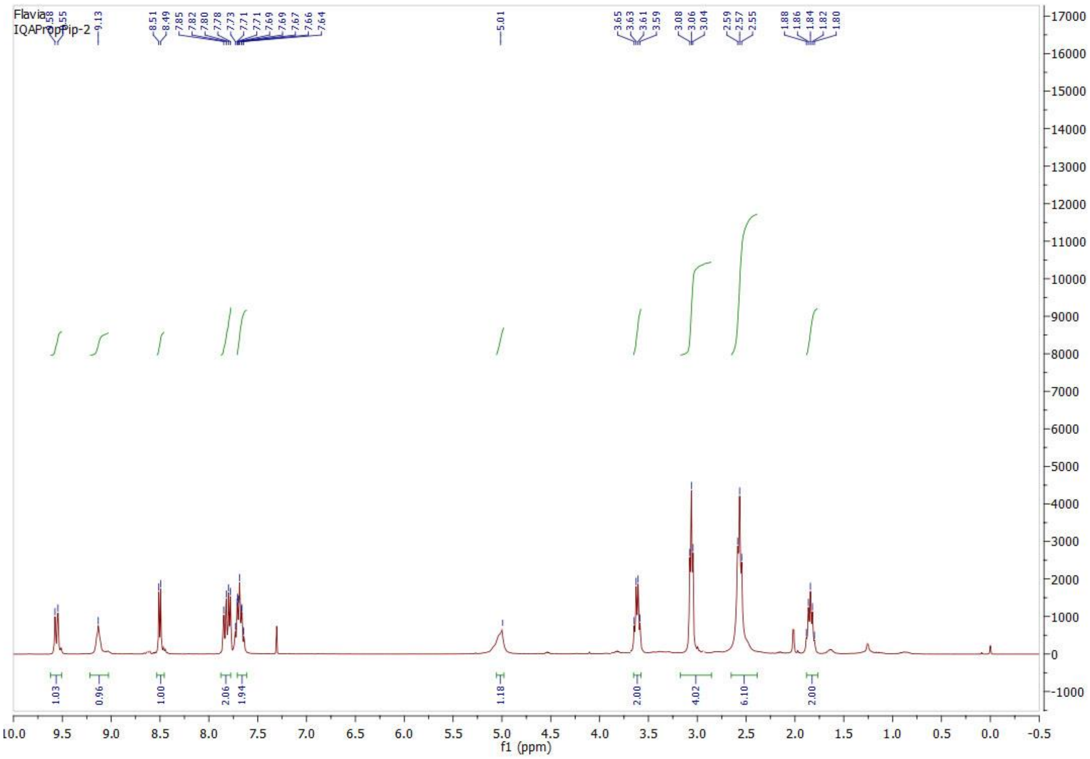

Figure S25. *N*-(3-Piperazin-1-ylpropyl)isoquinoline-1-carboxamide (**LINS05410**). ^{1}H NMR (300 MHz, $CDCl_3$) δ 9.56 (d, J = 9.0 Hz, 1H), 9.13 (s, 1H), 8.50 (d, J = 5.5 Hz, 1H), 7.88 – 7.75 (m, 2H), 7.76 – 7.56 (m, 2H), 5.01 (s, 1H), 3.62 (dd, J = 12.0, 6.1 Hz, 2H), 3.14 – 2.95 (m, 4H), 2.74 – 2.35 (m, 6H), 1.84 (quint, J = 6.1 Hz, 2H).

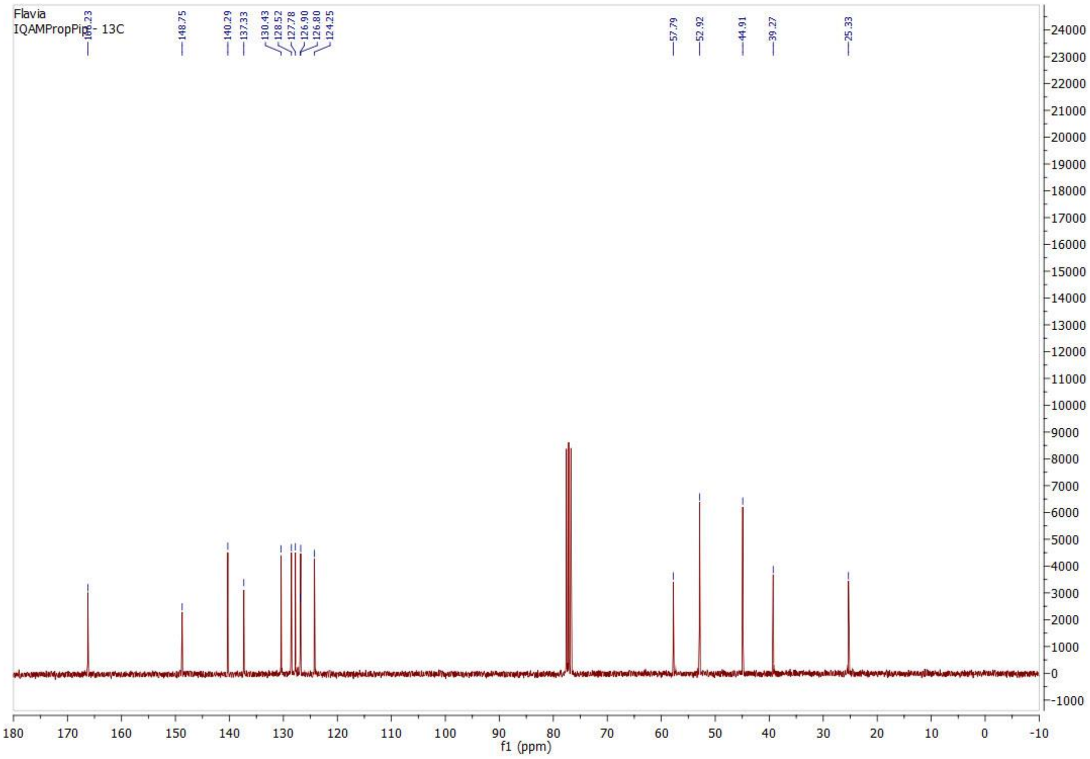

Figure S26. *N*-(3-Piperazin-1-ylpropyl)isoquinoline-1-carboxamide (**LINS05410**). ^{13}C NMR (75 MHz, $CDCl_3$) δ 166.2, 148.8, 140.3, 137.3, 130.4, 128.5, 127.8, 126.9, 126.8, 124.2, 57.8, 52.9, 44.9, 39.3, 25.3.

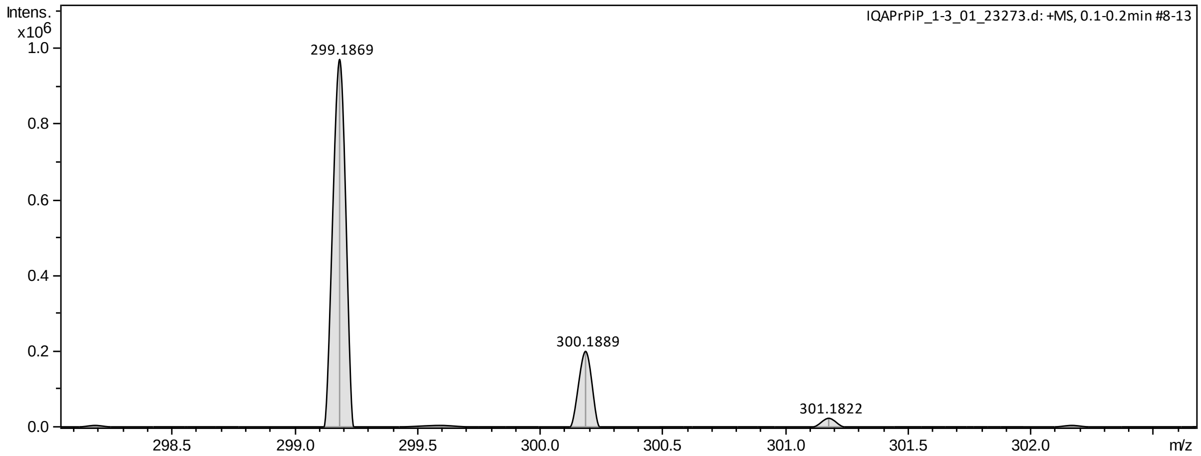

Figure S27. *N*-(3-Piperazin-1-ylpropyl)isoquinoline-1-carboxamide (**LINS05410**). HRMS (ESI) *m/z*: $[M+H]^+$ calcd.: 299.1866; $[M+H]^+$ found: 299.1869.

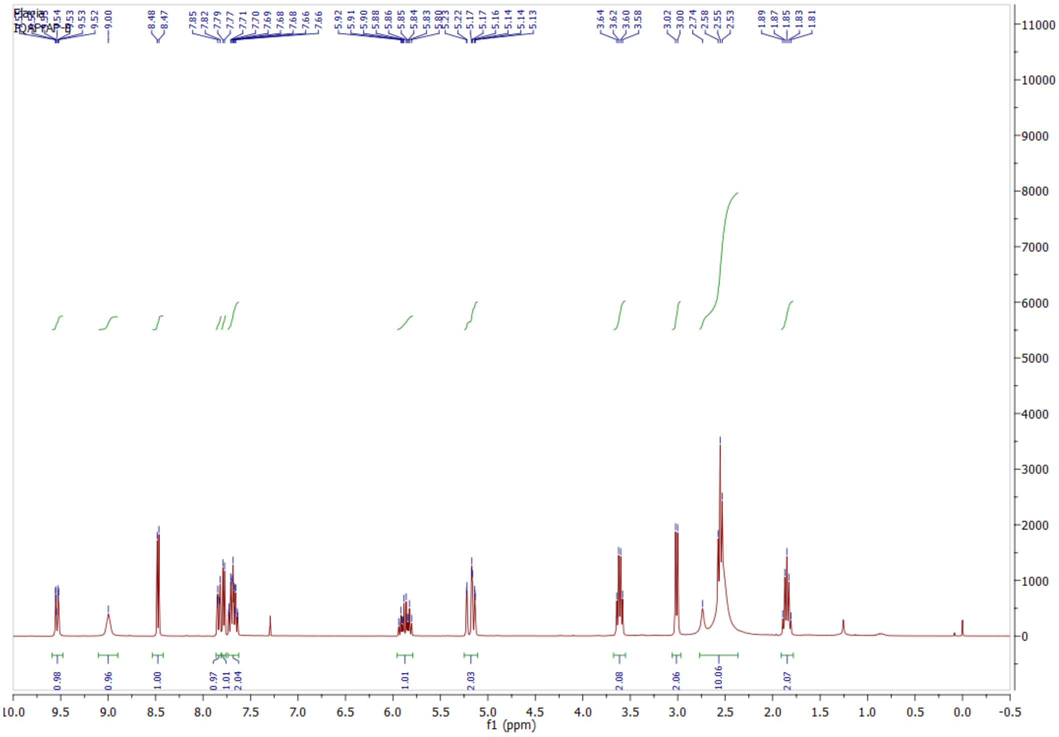

Figure S28. *N*-[3-(4-Allylpiperazin-1-yl)propyl]isoquinoline-1-carboxamide (**LINS05412**). ^{1}H NMR (300 MHz, $CDCl_3$) δ 9.59 – 9.48 (m, 1H), 9.00 (s, 1H), 8.47 (d, *J* = 5.5 Hz, 1H), 7.84 (dd, *J* = 7.2, 2.1 Hz, 1H), 7.78 (d, *J* = 5.5 Hz, 1H), 7.74 – 7.60 (m, 2H), 5.87 (ddt, *J* = 16.8, 10.1, 6.6 Hz, 1H), 5.26 – 5.09 (m, 2H), 3.61 (dd, *J* = 12.5, 6.3 Hz, 2H), 3.01 (d, *J* = 6.6 Hz, 2H), 2.78 – 2.34 (m, 10H), 1.85 (quint, *J* = 6.3 Hz, 2H).

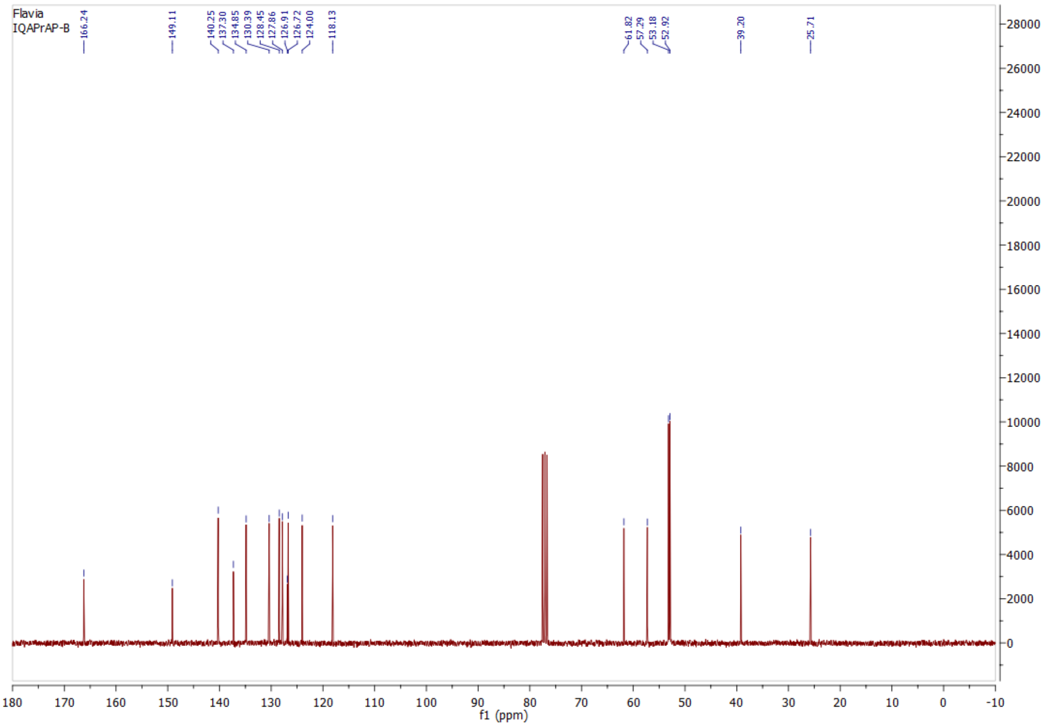

Figure S29. *N*-[3-(4-Allylpiperazin-1-yl)propyl]isoquinoline-1-carboxamide (**LINS05412**). ^{13}C NMR (75 MHz, $CDCl_3$) δ 166.2, 149.1, 140.2, 137.3, 134.8, 130.4, 128.4, 127.9, 126.9, 126.7, 124.0, 118.1, 61.8, 57.3, 53.2, 52.9, 39.2, 25.7.

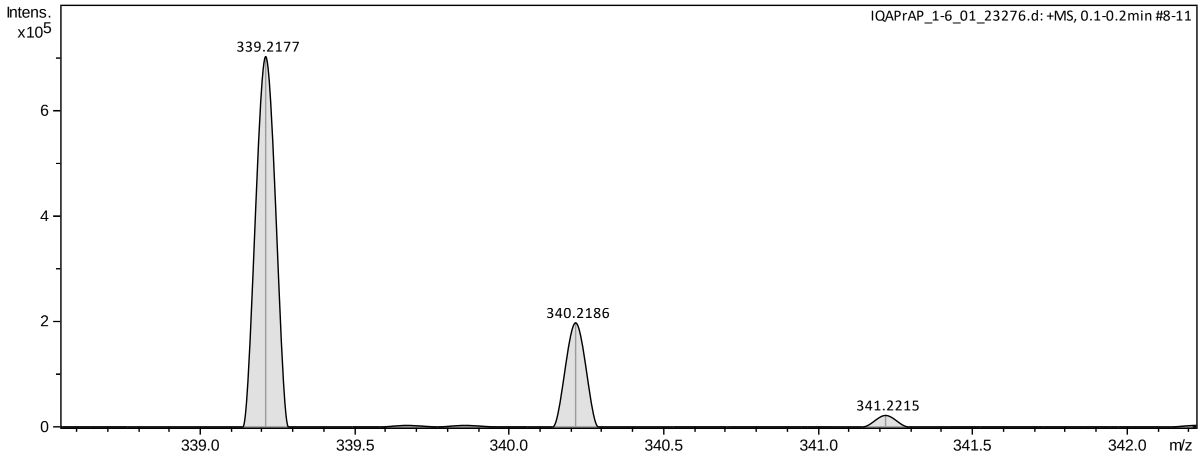

Figure S30. *N*-[3-(4-Allylpiperazin-1-yl)propyl]isoquinoline-1-carboxamide (**LINS05412**). HRMS (ESI) *m/z*: $[M+H]^+$ calcd.: 339.2179; $[M+H]^+$ found: 339.2177.

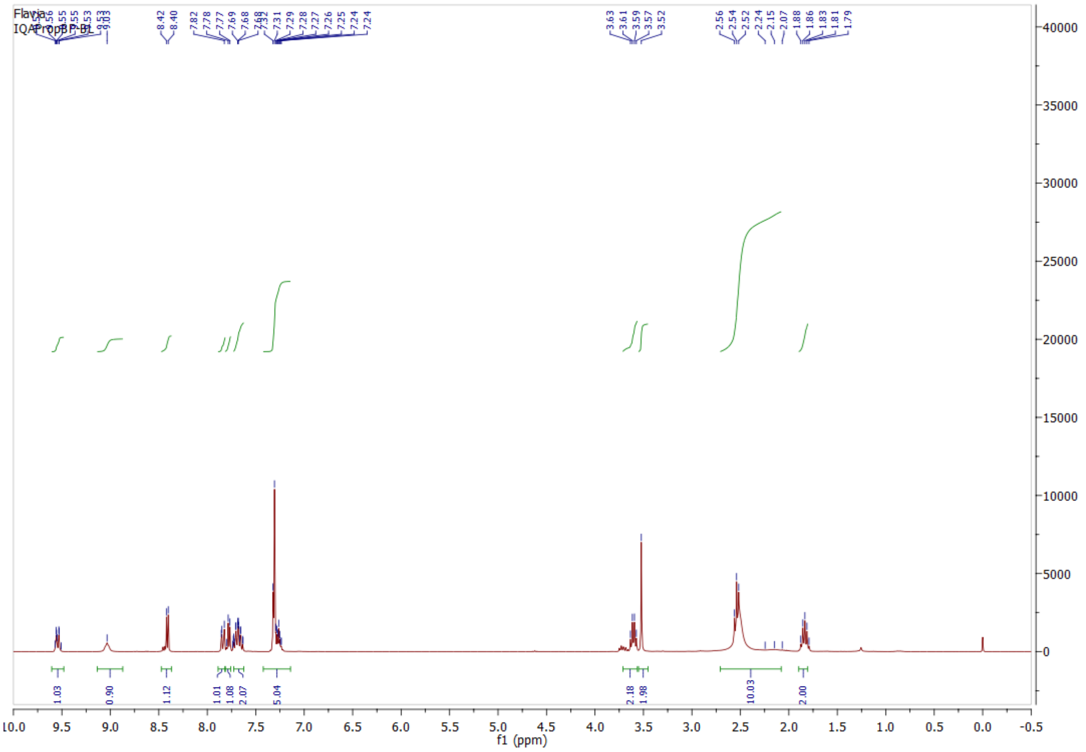

Figure S31. *N*-[3-(4-Benzylpiperazin-1-yl)propyl]isoquinoline-1-carboxamide (**LINS05413**). ^{1}H NMR (300 MHz, $CDCl_3$) δ 9.60 – 9.49 (m, 1H), 9.03 (s, 1H), 8.41 (d, J = 5.5 Hz, 1H), 7.88 – 7.81 (m, 1H), 7.78 (d, J = 5.5 Hz, 1H), 7.75 – 6.61 (m, 2H), 7.40 – 7.15 (m, 5H), 3.60 (dd, J = 12.4, 6.2 Hz, 2H), 3.52 (s, 2H), 2.81 – 1.99 (m, 10H), 1.83 (quint, J = 6.2 Hz, 2H).

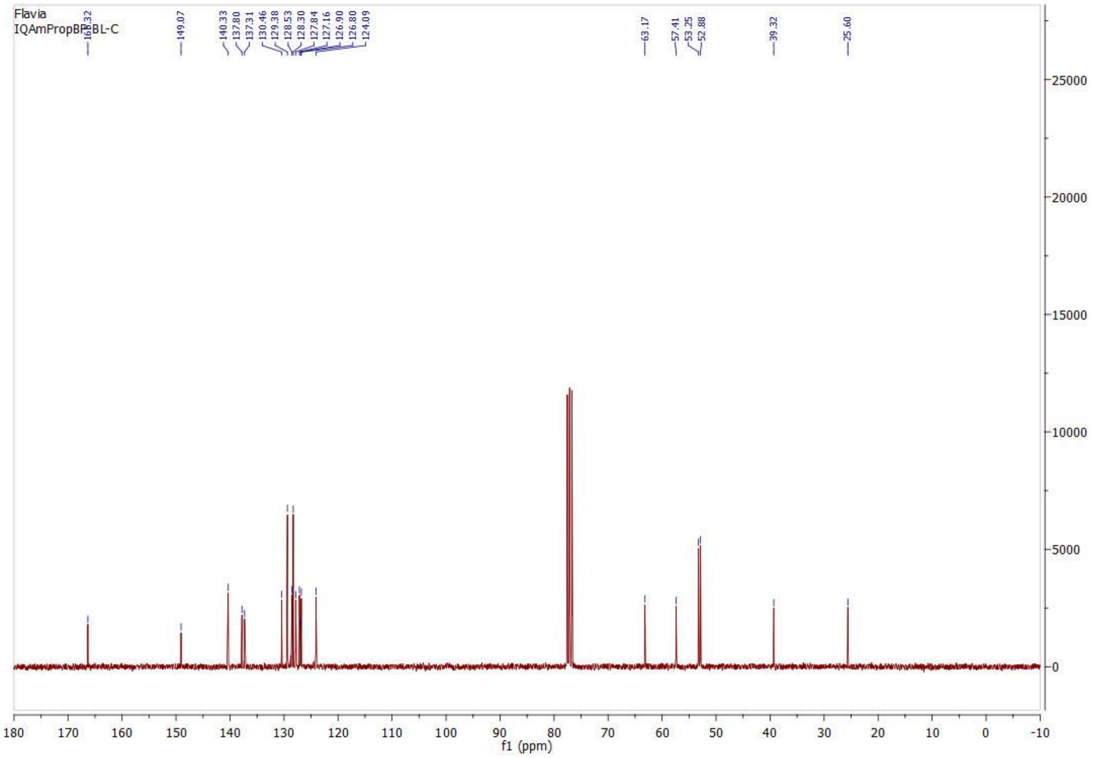

Figure S32. *N*-[3-(4-Benzylpiperazin-1-yl)propyl]isoquinoline-1-carboxamide (**LINS05413**). ^{13}C NMR (75 MHz, $CDCl_3$) δ 166.3, 149.1, 140.3, 137.8, 137.3, 130.5, 129.4, 128.5, 128.3, 127.8, 127.2, 126.9, 126.8, 124.1, 63.2, 57.4, 53.2, 52.9, 39.3, 25.6.

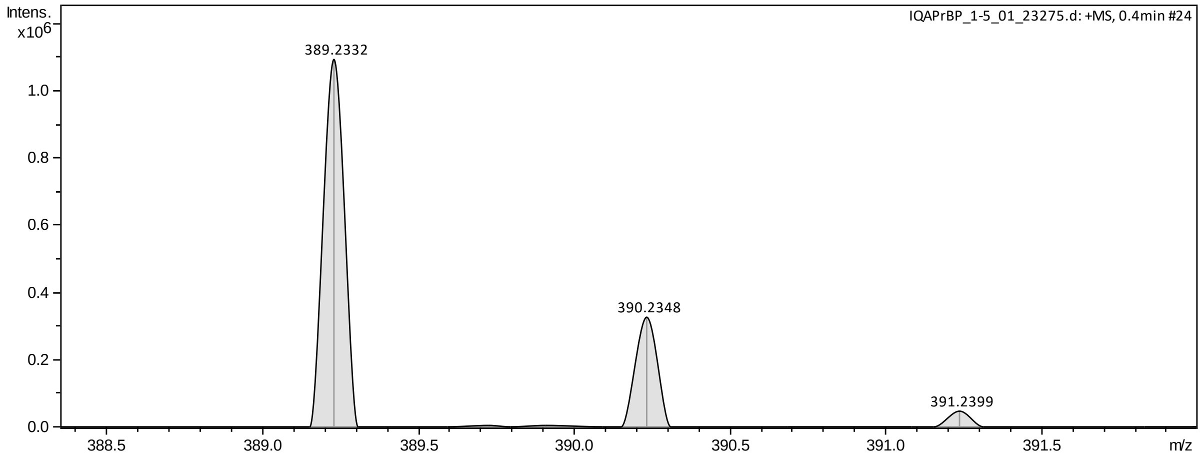

Figure S33. *N*-[3-(4-Benzylpiperazin-1-yl)propyl]isoquinoline-1-carboxamide (**LINS05413**). HRMS (ESI) *m/z*: $[M+H]^+$ calcd.: 389.2335; $[M+H]^+$ found: 389.2332.

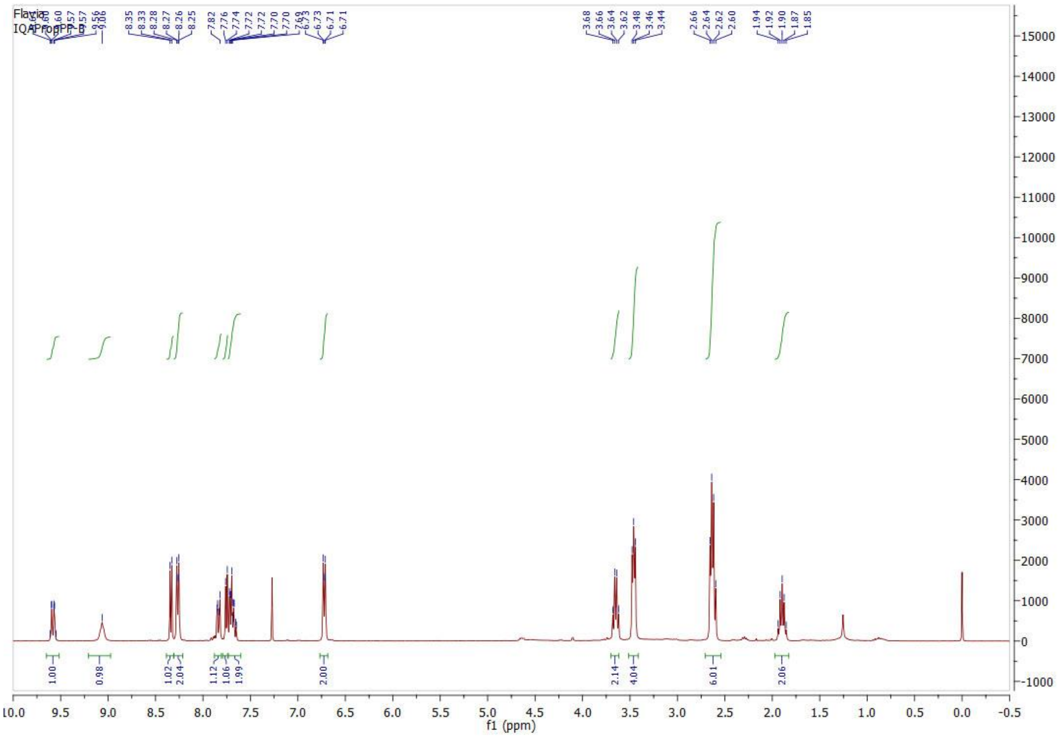

Figure S34. *N*-[3-[4-(4-Pyridyl)piperazin-1-yl]propyl]isoquinoline-1-carboxamide (**LINS05414**). ^{1}H NMR (300 MHz, $CDCl_3$) δ 9.62 – 9.55 (m, 1H), 9.06 (s, 1H), 8.34 (d, J = 5.4 Hz, 1H), 8.27 (dd, J = 5.5, 1.2 Hz, 2H), 7.86 – 7.81 (m, 1H), 7.75 (d, J = 5.4 Hz, 1H), 7.73 – 7.63 (m, 2H), 6.72 (dd, J = 5.5, 1.2 Hz, 2H), 3.65 (dd, J = 11.8, 6.1 Hz, 2H), 3.51 – 3.39 (m, 4H), 2.70 – 2.55 (m, 6H), 1.90 (quint, J = 6.1 Hz, 2H).

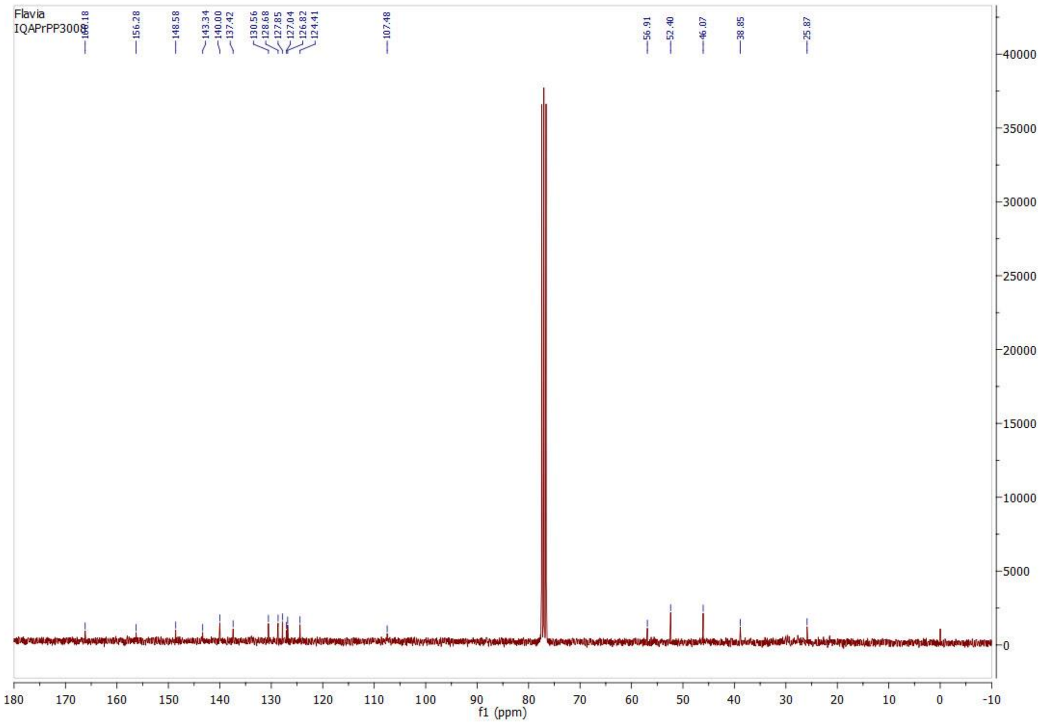

Figure S35. *N*-[3-[4-(4-Pyridyl)piperazin-1-yl]propyl]isoquinoline-1-carboxamide (**LINS05414**). ^{13}C NMR (75 MHz, $CDCl_3$) δ 166.2, 156.3, 148.6, 143.3, 140.0, 137.4, 130.6, 128.7, 127.8, 127.0, 126.8, 124.4, 107.5, 56.9, 52.4, 46.1, 38.8, 25.9.

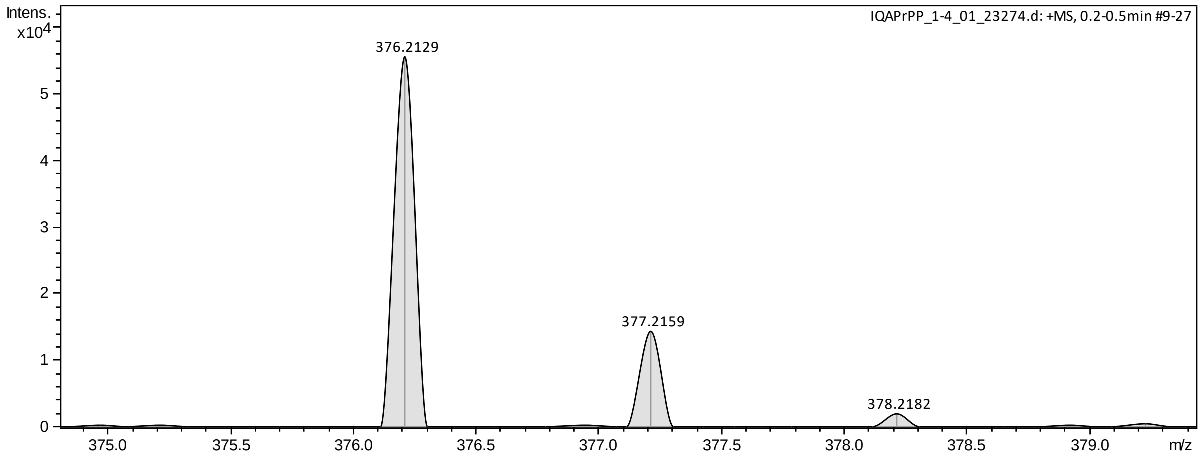

Figure S36. *N*-[3-[4-(4-Pyridyl)piperazin-1-yl]propyl]isoquinoline-1-carboxamide (**LINS05414**). HRMS (ESI) *m/z*: $[M+H]^+$ calcd.: 376.2131; $[M+H]^+$ found: 376.2129.

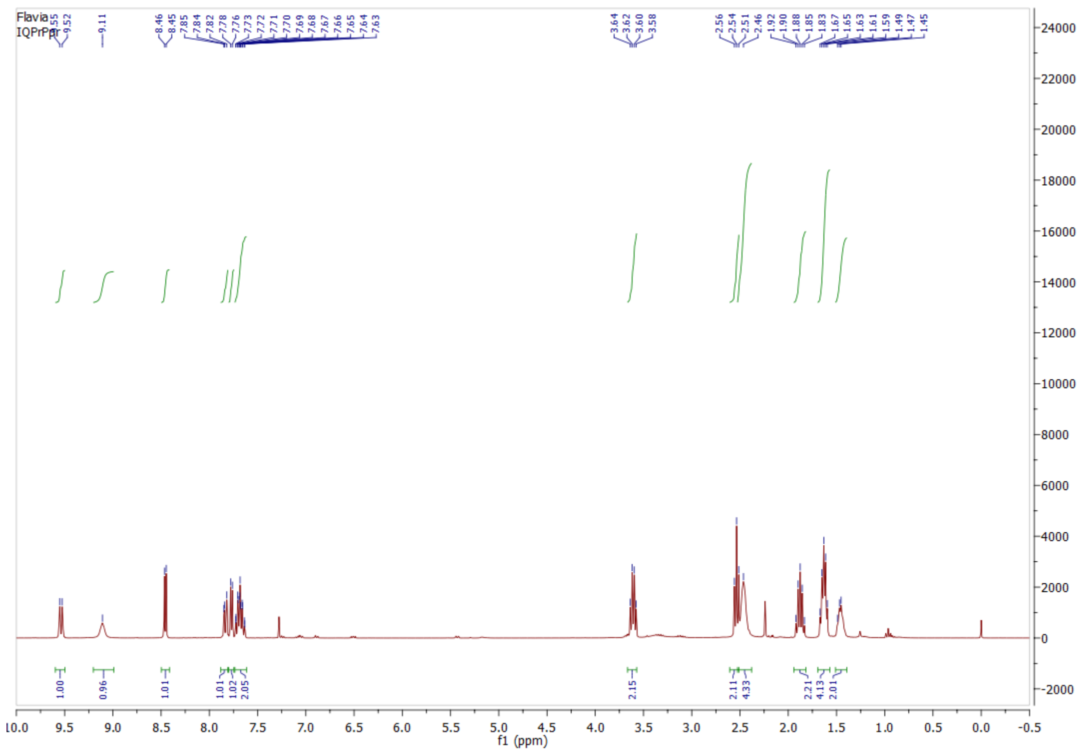

Figure S37. *N*-[3-(1-Piperidyl)propyl]isoquinoline-1-carboxamide (**LINS05415**). ^{1}H NMR (300 MHz, $CDCl_3$) δ 9.54 (d, J = 7.5 Hz, 1H), 9.11 (s, 1H), 8.46 (d, J = 5.5 Hz, 1H), 7.83 (d, J = 7,5 Hz, 1H), 7.77 (d, J = 5.5 Hz, 1H), 7.74 – 7.58 (m, 2H), 3.61 (dd, J = 12.4, 6.6 Hz, 2H), 2.54 (t, J = 6.6 Hz, 2H), 2.52 – 2.33 (m, 4H), 1.88 (quint, J = 6.6 Hz, 2H), 1.72 – 1.55 (m, 4H), 1.53 – 1.35 (m, 2H).

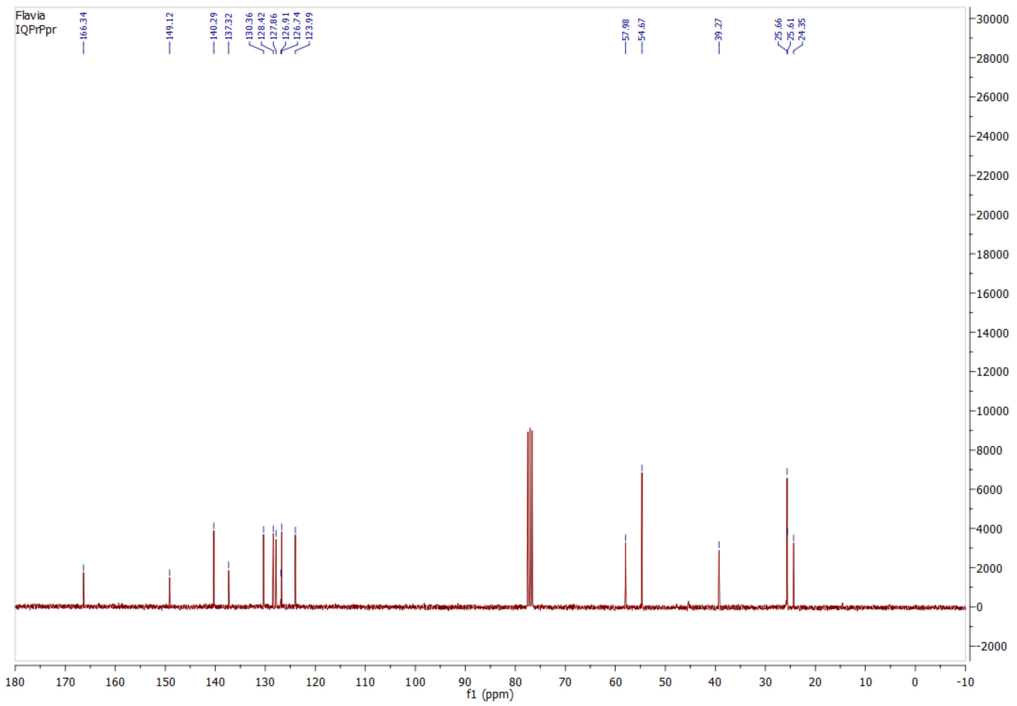

Figure S38. *N*-[3-(1-Piperidyl)propyl]isoquinoline-1-carboxamide (**LINS05415**). ^{13}C NMR (75 MHz, $CDCl_3$) δ 166.3, 149.1, 140.3, 137.3, 130.4, 128.4, 127.9, 126.9, 126.7, 123.9, 57.9, 54.7, 39.3, 25.7, 25.6, 24.3.

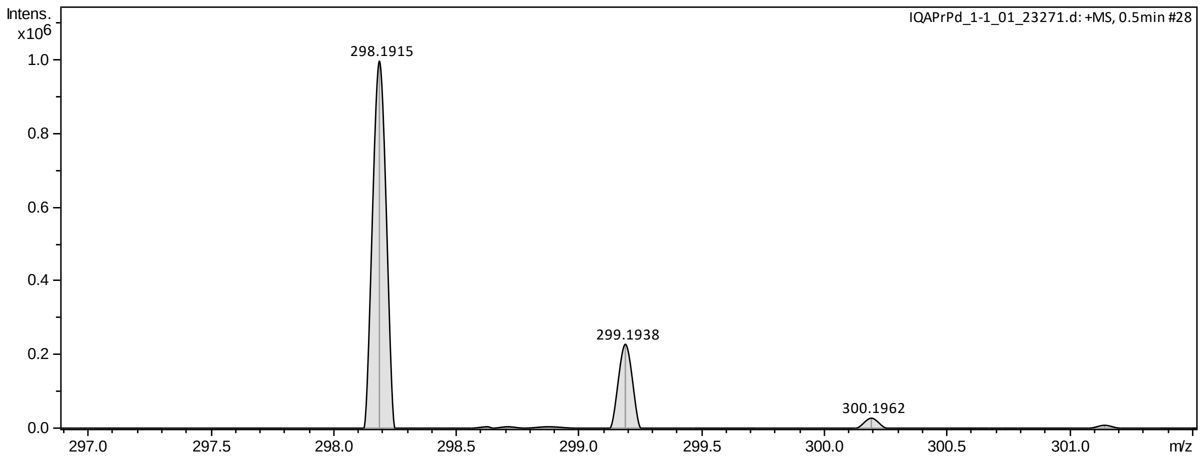

Figure S39. *N*-[3-(1-Piperidyl)propyl]isoquinoline-1-carboxamide (**LINS05415**). HRMS (ESI) *m/z*: $[M+H]^+$ calcd.: 298.1913; $[M+H]^+$ found: 298.1915.

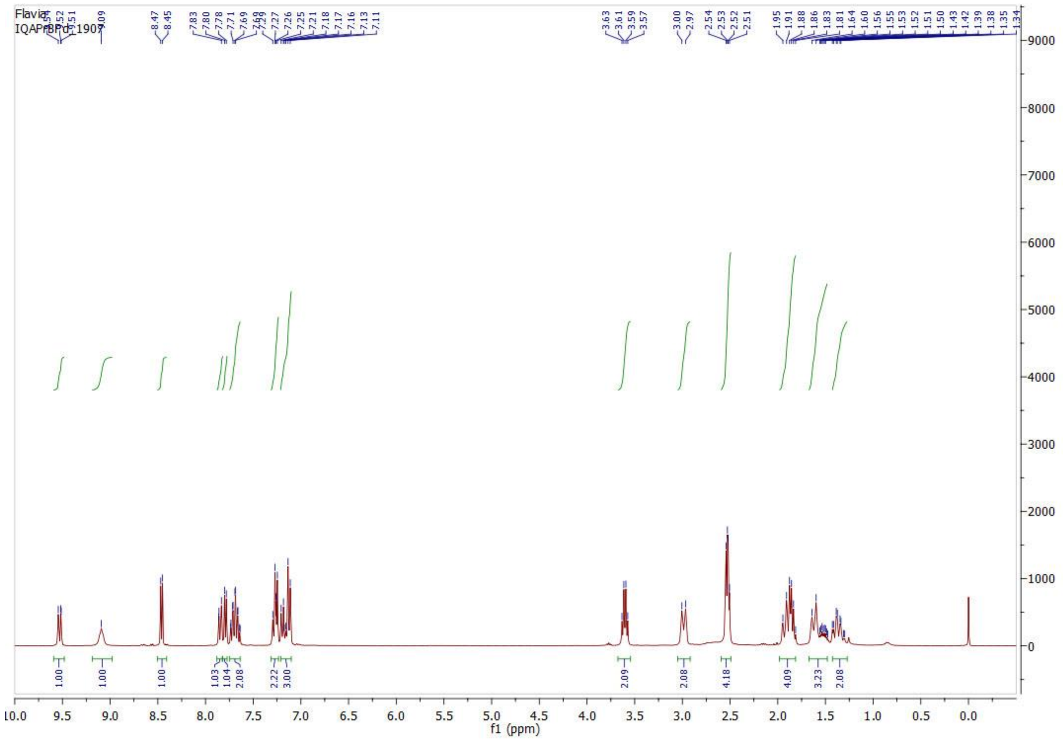

Figure S40. *N*-[3-(4-Benzyl-1-piperidyl)propyl]isoquinoline-1-carboxamide (**LINS05416**). ^{1}H NMR (300 MHz, $CDCl_3$) δ 9.58 – 9.48 (m, 1H), 9.09 (s, 1H), 8.46 (d, J = 5.5 Hz, 1H), 7.88 – 7.82 (m, 1H), 7.79 (d, J = 5.5 Hz, 1H), 7.76 – 7.62 (m, 2H), 7.32 – 7.23 (m, 2H), 7.23 – 7.09 (m, 3H), 3.60 (dd, J = 12.3, 5.9 Hz, 2H), 2.99 (d, J = 11.6 Hz, 2H), 2.58 – 2.47 (m, 4H), 1.99 – 1.77 (m, 4H), 1.67 – 1.45 (m, 3H), 1.46 – 1.28 (m, 2H).

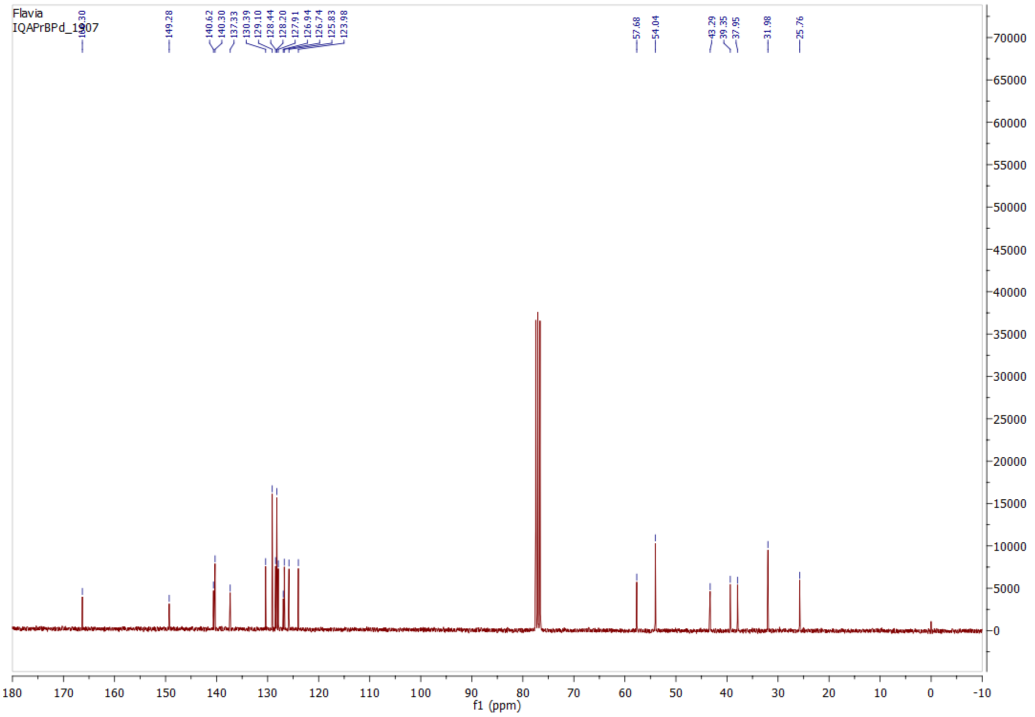

Figure S41. *N*-[3-(4-Benzyl-1-piperidyl)propyl]isoquinoline-1-carboxamide (**LINS05416**). ^{13}C NMR (75 MHz, $CDCl_3$) δ 166.3, 149.3, 140.6, 140.3, 137.33, 130.4, 129.1, 128.4, 128.2, 127.9, 126.9, 126.7, 125.8, 124.0, 57.7, 54.0, 43.3, 39.3, 37.9, 32.0, 25.8.

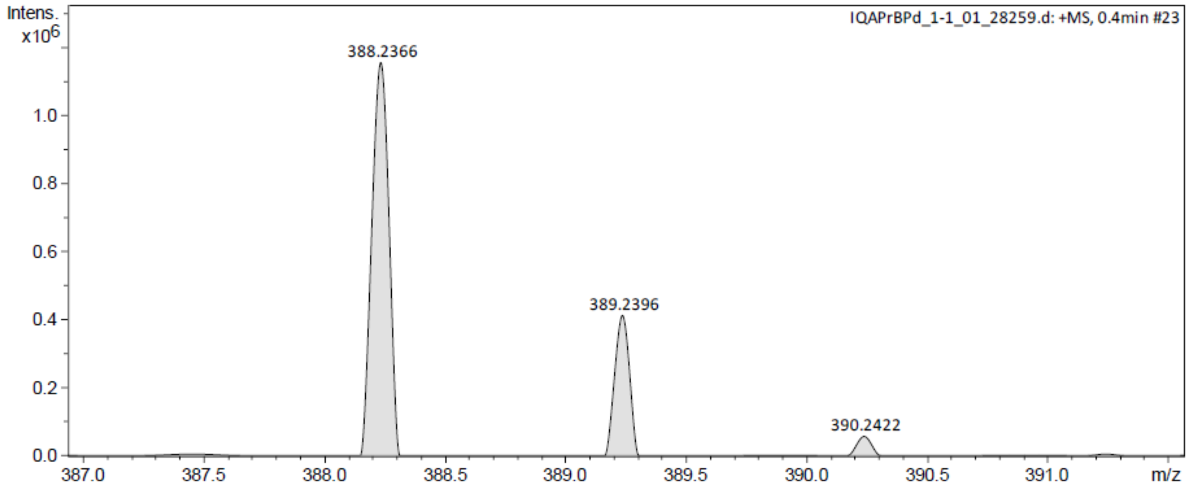

Figure S42. *N*-[3-(4-Benzyl-1-piperidyl)propyl]isoquinoline-1-carboxamide (**LINS05416**). HRMS (ESI) *m/z*: $[M+H]^+$ calcd.: 388.2383; $[M+H]^+$ found: 388.2366.

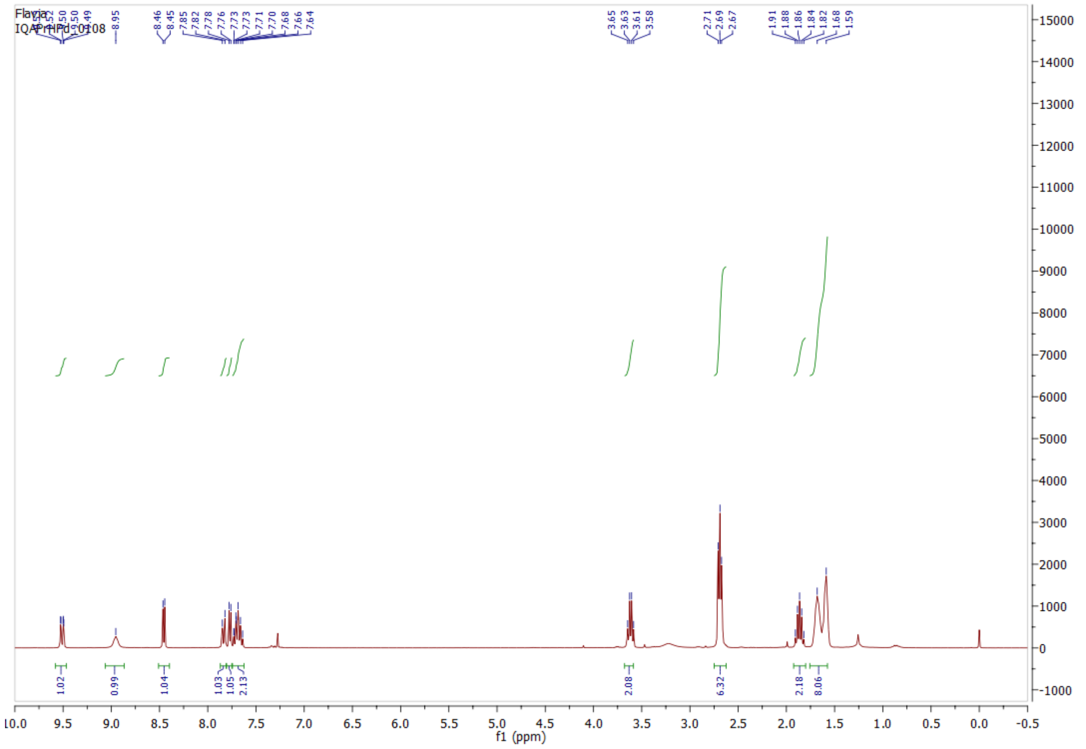

Figure S43. *N*-[3-(Azepan-1-yl)propyl]isoquinoline-1-carboxamide (**LINS05417**). ^{1}H NMR (300 MHz, $CDCl_3$) δ 9.56 – 9.45 (m, 1H), 8.95 (s, 1H), 8.46 (d, J = 5.5 Hz, 1H), 7.84 (d, J = 7.9 Hz, 1H), 7.77 (d, J = 5.6 Hz, 1H), 7.74 – 7.61 (m, 2H), 3.62 (dd, J = 12.3, 6.3 Hz, 2H), 2.77 – 2.59 (m, 6H), 1.86 (quint, J = 6.3 Hz, 2H), 1.76 – 1.51 (m, 8H).

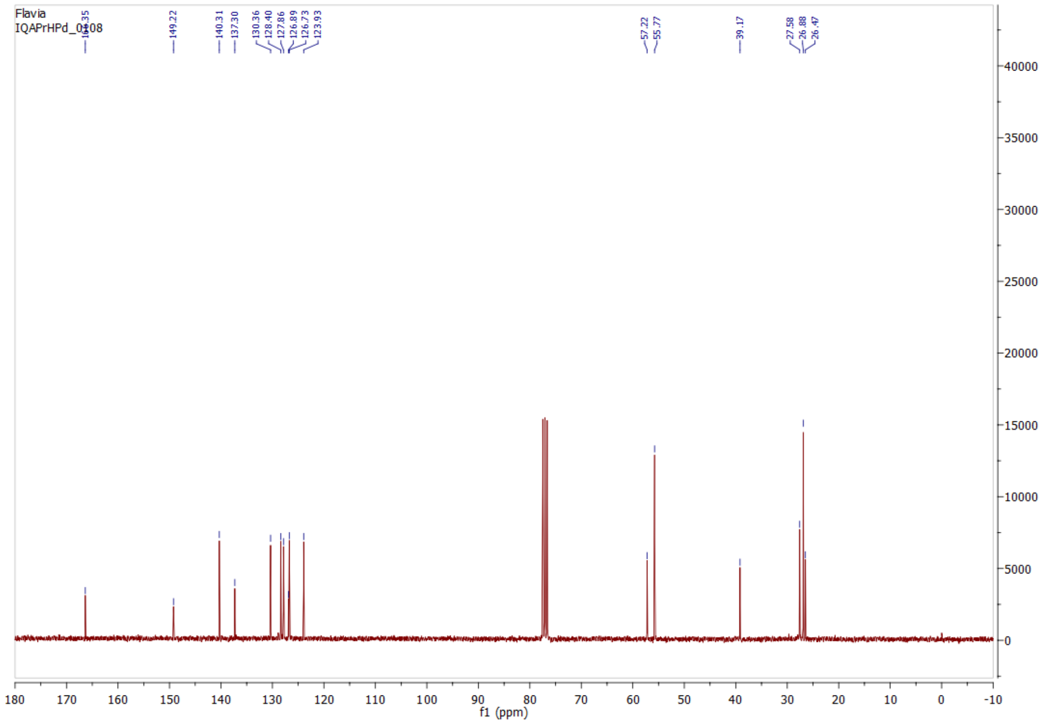

Figure S44. *N*-[3-(Azepan-1-yl)propyl]isoquinoline-1-carboxamide (**LINS05417**). ^{13}C NMR (75 MHz, $CDCl_3$) δ 166.3, 149.2, 140.3, 137.3, 130.4, 128.4, 127.9, 126.9, 126.7, 123.9, 57.2, 55.8, 39.2, 27.6, 26.9, 26.5.

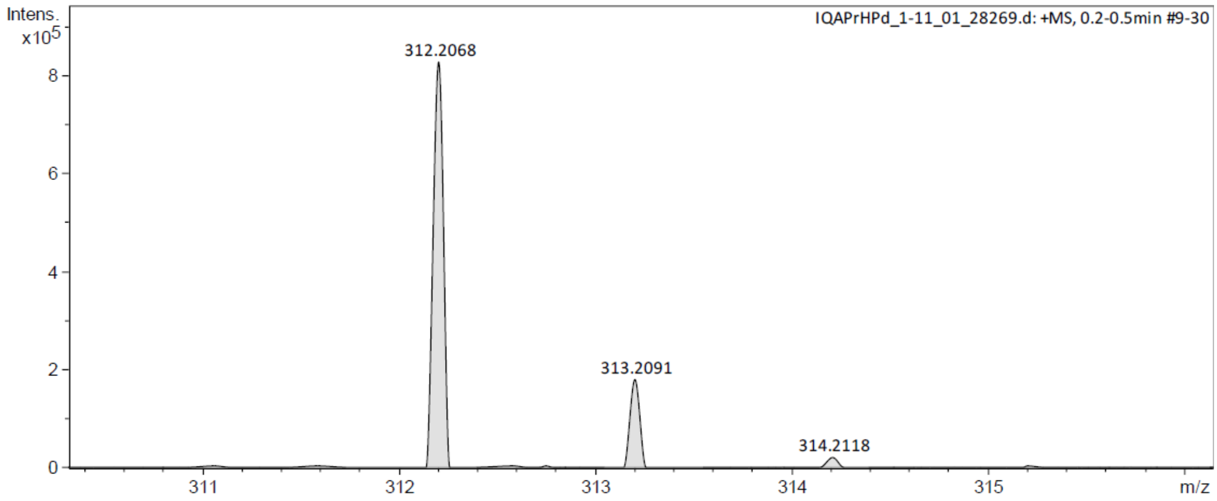

Figure S45. *N*-[3-(Azepan-1-yl)propyl]isoquinoline-1-carboxamide (**LINS05417**). HRMS (ESI) *m/z*: $[M+H]^+$ calcd.: 312.2070; $[M+H]^+$ found: 312.2068.

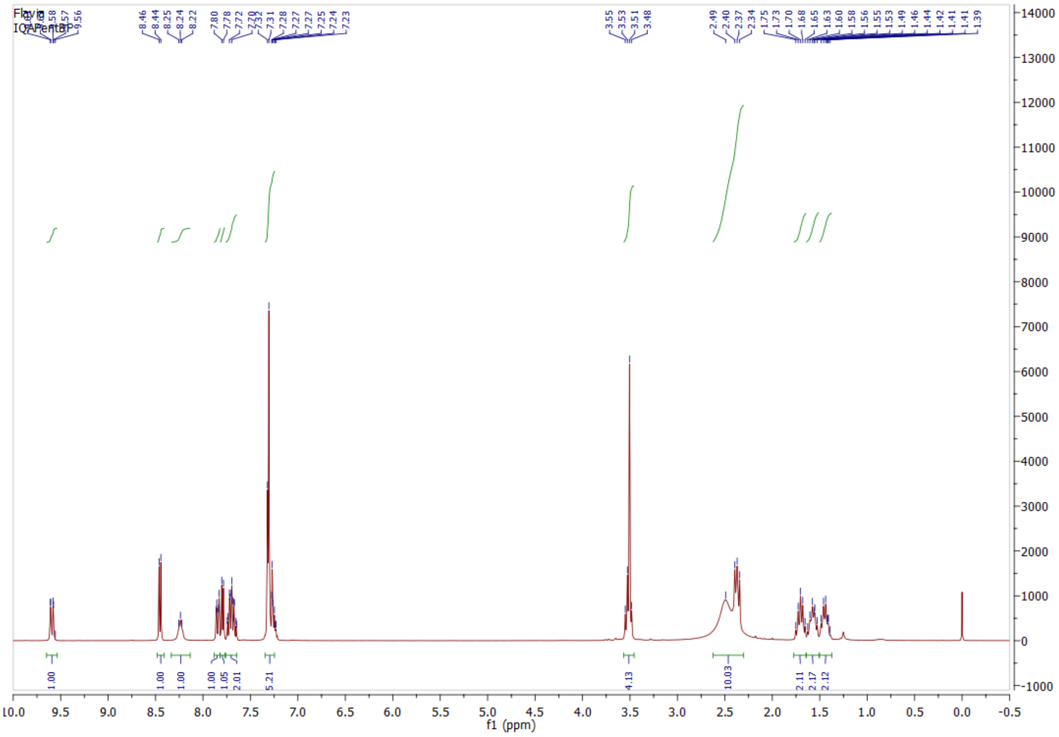

Figure S46. *N*-[5-(4-Benzylpiperazin-1-yl)pentyl)isoquinoline-1-carboxamide (**LINS05433**). ^{1}H NMR (300 MHz, $CDCl_3$) δ 9.64 – 9.54 (m, 1H), 8.45 (d, J = 5.5 Hz, 1H), 8.24 (t, J = 4.9 Hz, 1H), 7.88 – 7.82 (m, 1H), 7.79 (d, J = 5.5 Hz, 1H), 7.76 – 7.62 (m, 2H), 7.37 – 7.19 (m, 5H), 3.57 – 3.45 (m, 4H), 2.79 – 2.10 (m, 10H), 1.77 – 1.64 (m, 2H), 1.64 – 1.51 (m, 2H), 1.51 – 1.35 (m, 2H).

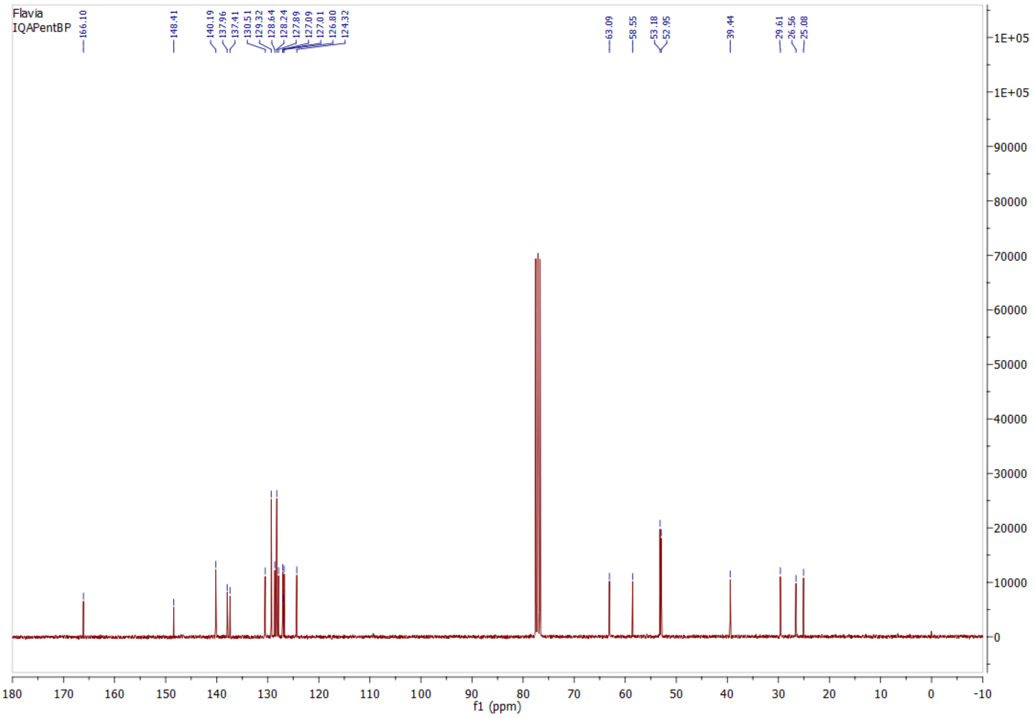

Figure S47. *N*-[5-(4-Benzylpiperazin-1-yl)pentyl)isoquinoline-1-carboxamide (**LINS05433**). ^{13}C NMR (75 MHz, $CDCl_3$) δ 166.1, 148.4, 140.2, 137.9, 137.4, 130.5, 129.3, 128.6, 128.2, 127.9, 127.1, 127.0, 126.8, 124.3, 63.1, 58.5, 53.2, 52.9, 39.4, 29.6, 26.6, 25.1.

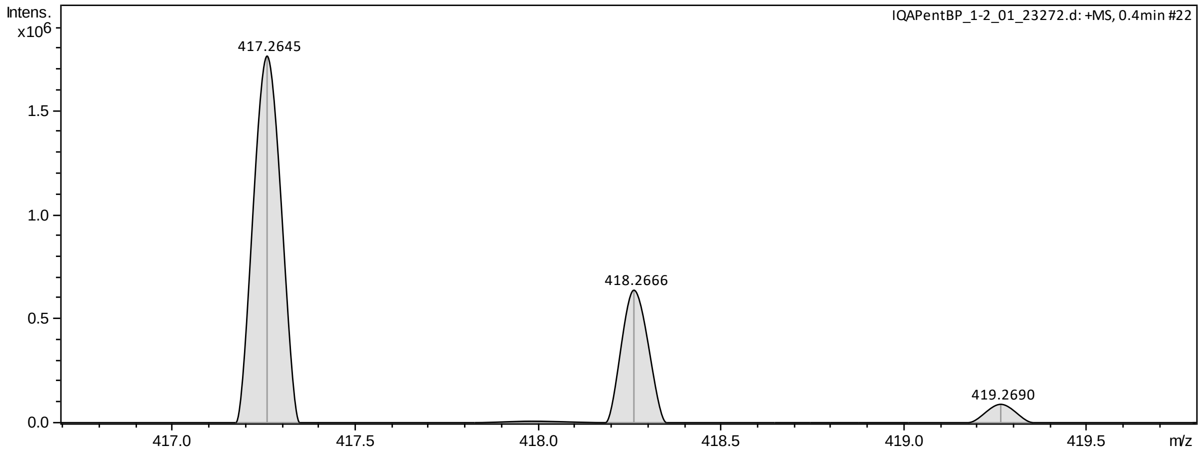

Figure S48. *N*-[5-(4-Benzylpiperazin-1-yl)pentyl)isoquinoline-1-carboxamide (**LINS05433**). HRMS (ESI) *m/z*: $[M+H]^+$ calcd.: 417.2648; $[M+H]^+$ found: 417.2645.

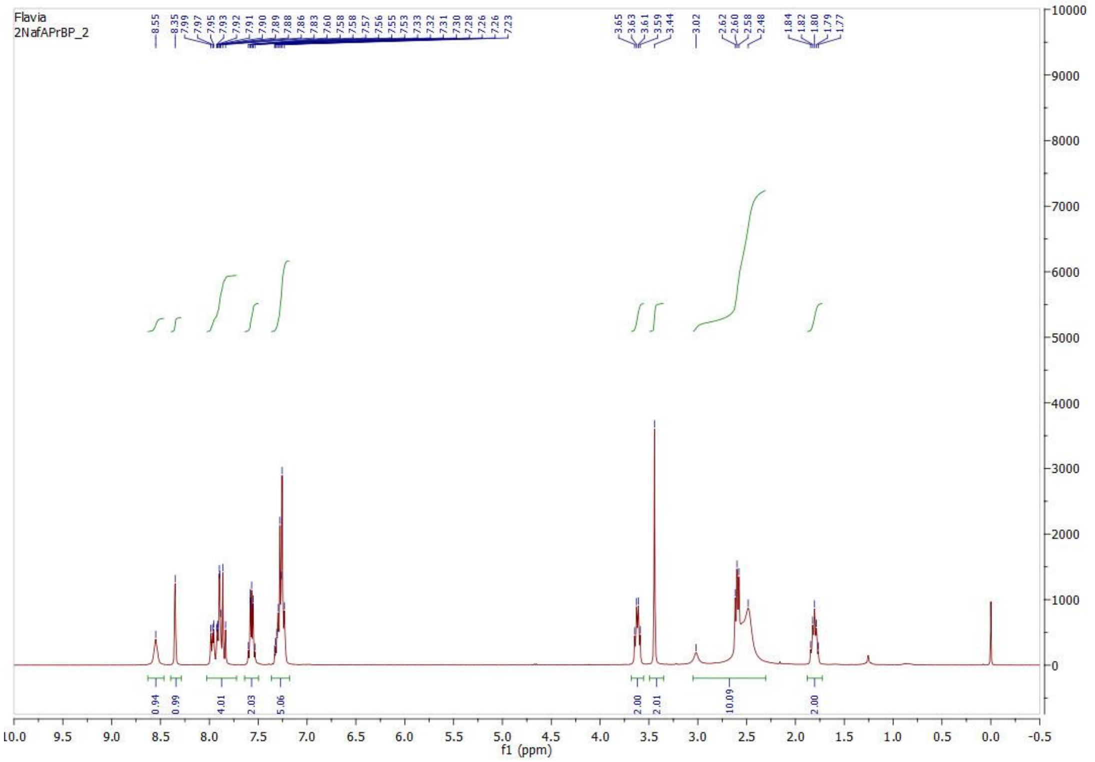

Figure S49. *N*-[3-(4-Benzylpiperazin-1-yl)propyl]naphthalene-2-carboxamide (**LINS05513**). ^{1}H NMR (300 MHz, $CDCl_3$) δ 8.55 (sl, 1H), 8.35 (s, 1H), 8.12 – 7.79 (m, 4H), 7.75 – 7.50 (m, 2H), 7.45 – 7.19 (m, 5H), 3.61 (dd, J = 10.9, 5.4 Hz, 2H), 3.44 (s, 2H), 2.93 – 2.24 (m, 10H), 1.80 (quint, J = 5.4 Hz, 2H).

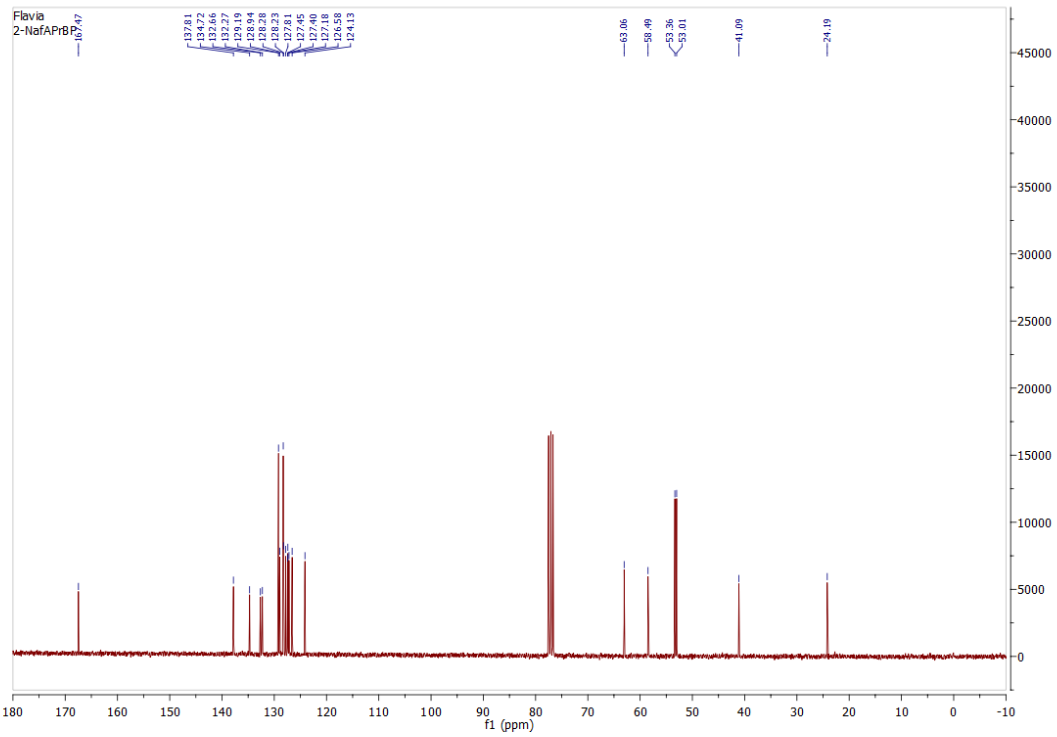

Figure S50. *N*-[3-(4-Benzylpiperazin-1-yl)propyl]naphthalene-2-carboxamide (**LINS05513**). ^{13}C NMR (75 MHz, $CDCl_3$) δ 167.5, 137.8, 134.7, 132.7, 132.3, 129.2, 128.9, 128.3, 128.2, 127.8, 127.4, 127.4, 127.2, 126.6, 124.1, 63.1, 58.5, 53.4, 53.0, 41.1, 24.2.

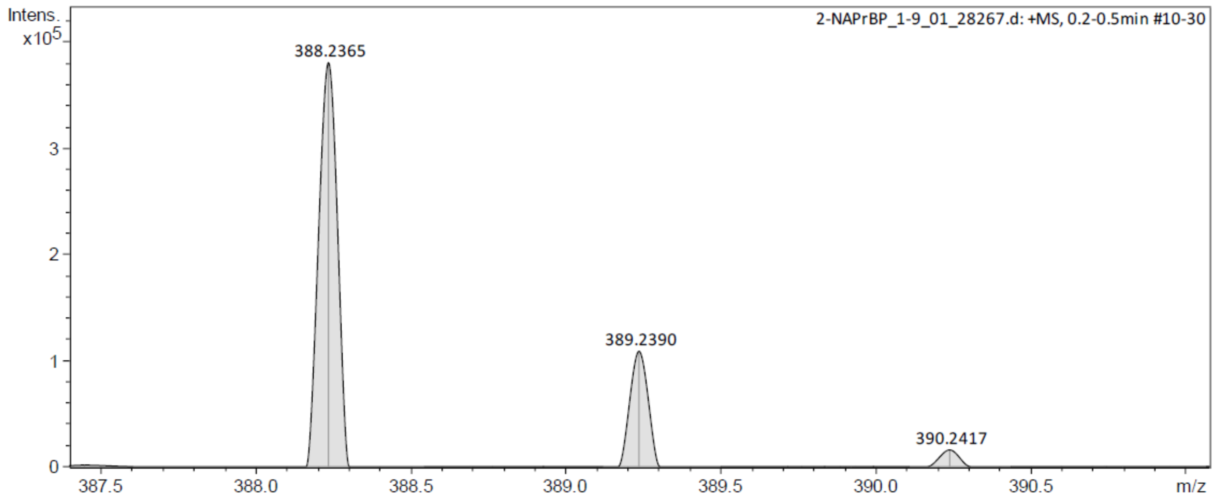

Figure S51. *N*-[3-(4-Benzylpiperazin-1-yl)propyl]naphthalene-2-carboxamide (**LINS05513**). HRMS (ESI) *m/z*: $[M+H]^+$ calcd.: 388.2383; $[M+H]^+$ found: 388.2365.

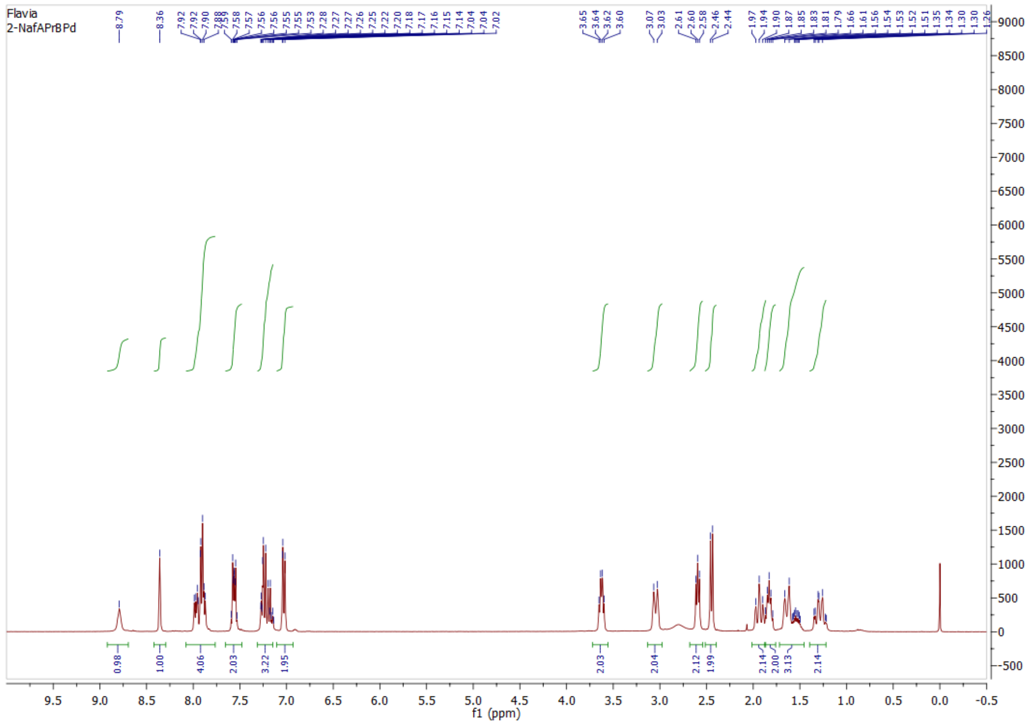

Figure S52. *N*-[3-(4-Benzyl-1-piperidyl)propyl]naphthalene-2-carboxamide (**LINS05516**). ^{1}H NMR (300 MHz, $CDCl_3$) δ 8.79 (sl, 1H), 8.36 (s, 1H), 8.02 – 7.84 (m, 4H), 7.62 – 7.50 (m, 2H), 7.31 – 7.11 (m, 3H), 7.08 – 6.98 (m, 2H), 3.63 (dd, *J* = 11.0, 5.6 Hz, 2H), 3.05 (d, *J* = 11.7 Hz, 2H), 2.67 – 2.54 (m, 2H), 2.45 (d, *J* = 7.0 Hz, 2H), 1.93 (t, *J* = 11.0 Hz, 2H), 1.83 (quint, *J* = 5.6 Hz, 2H), 1.71 – 1.45 (m, 3H), 1.38 – 1.17 (m, 2H).

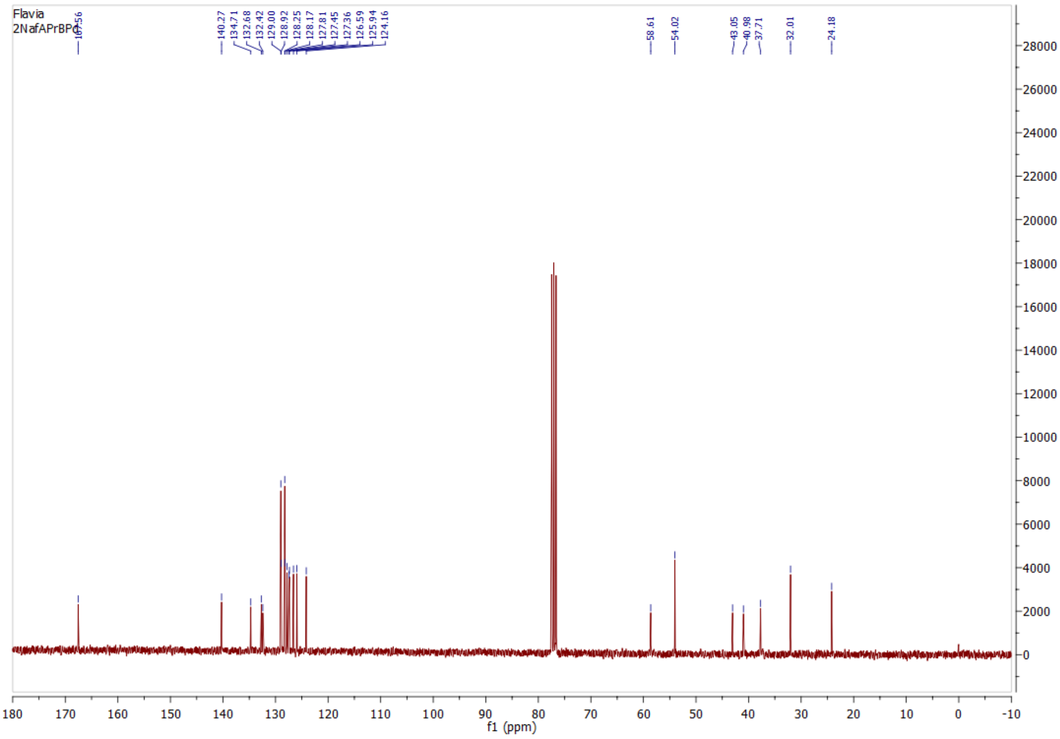

Figure S53. *N*-[3-(4-Benzyl-1-piperidyl)propyl]naphthalene-2-carboxamide (**LINS05516**). ^{13}C NMR (75 MHz, $CDCl_3$) δ 167.6, 140.3, 134.7, 132.7, 132.4, 129.0, 128.9, 128.2, 128.2, 127.8, 127.4, 127.4, 126.6, 125.9, 124.2, 58.6, 54.0, 43.0, 41.0, 37.7, 32.0, 24.2.

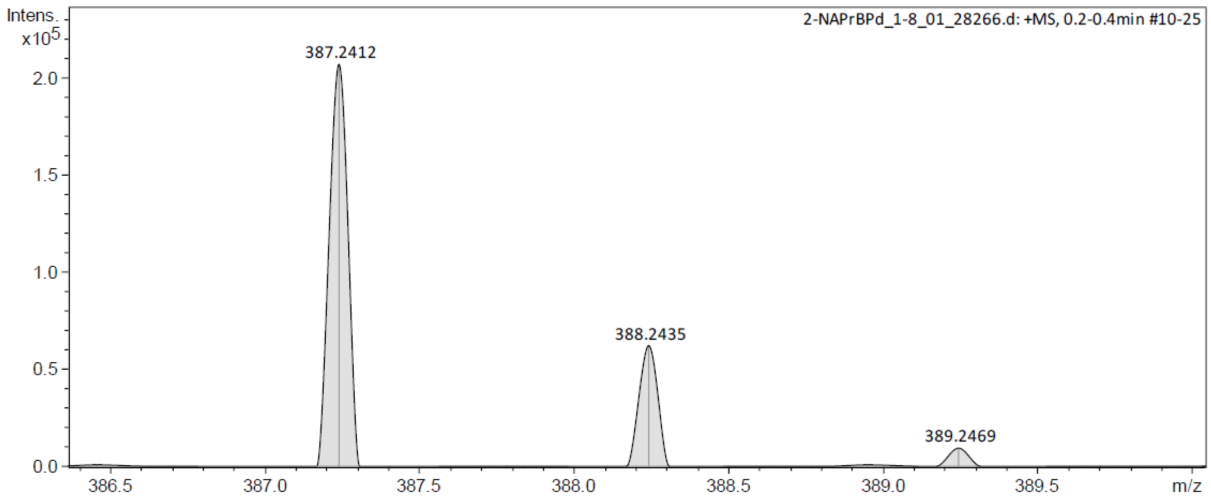

Figure S54. *N*-[3-(4-Benzyl-1-piperidyl)propyl]naphthalene-2-carboxamide (**LINS05516**). HRMS (ESI) *m/z*: $[M+H]^+$ calcd.: 387.2430; $[M+H]^+$ found: 387.2412.

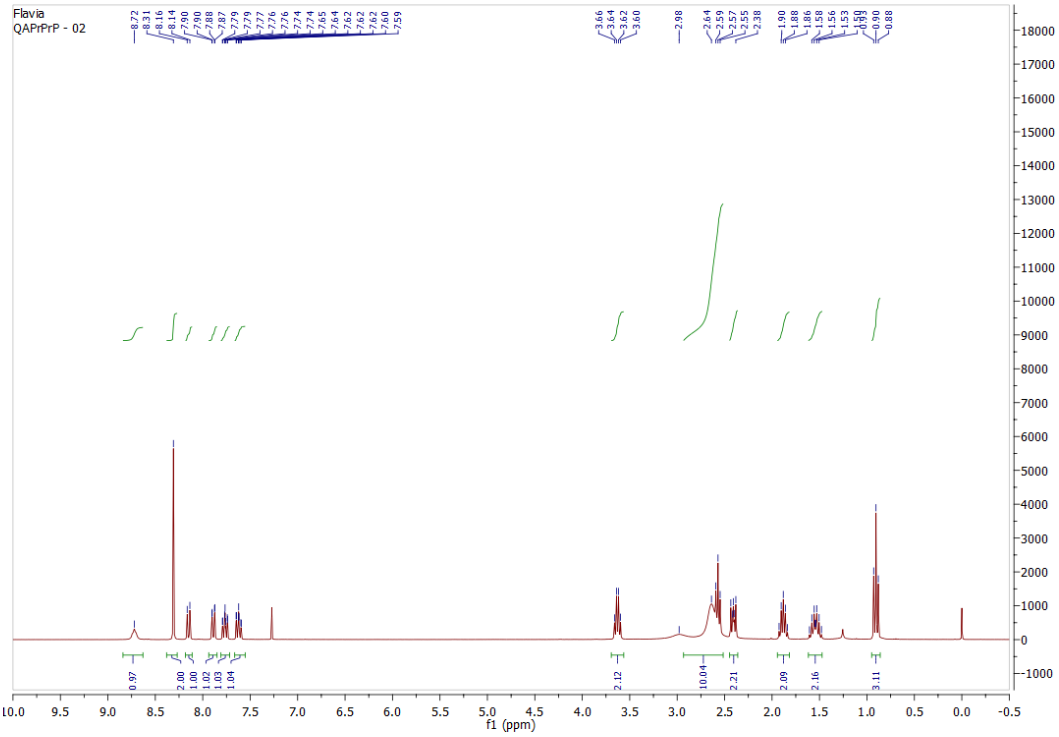

Figure S55. *N*-[3-(4-Propylpiperazin-1-yl)propyl]quinoline-2-carboxamide (**LINS05611**). ^{1}H NMR (300 MHz, $CDCl_3$) δ 8.72 (s, 1H), 8.31 (s, 2H), 8.15 (d, J = 8.3 Hz, 1H), 7.89 (dd, J = 8.5, 1.1 Hz, 1H), 7.76 (ddd, J = 8.3, 7.0, 1.1 Hz, 1H), 7.62 (ddd, J = 8.5, 7.0, 0.8 Hz, 1H), 3.63 (dd, J = 11.8, 6.5 Hz, 2H), 3.02 – 2.52 (m, 10H), 2.45 – 2.36 (m, 2H), 1.88 (quint, J = 6.5 Hz, 2H), 1.62 – 1.46 (m, 2H), 0.90 (t, J = 7.4 Hz, 3H).

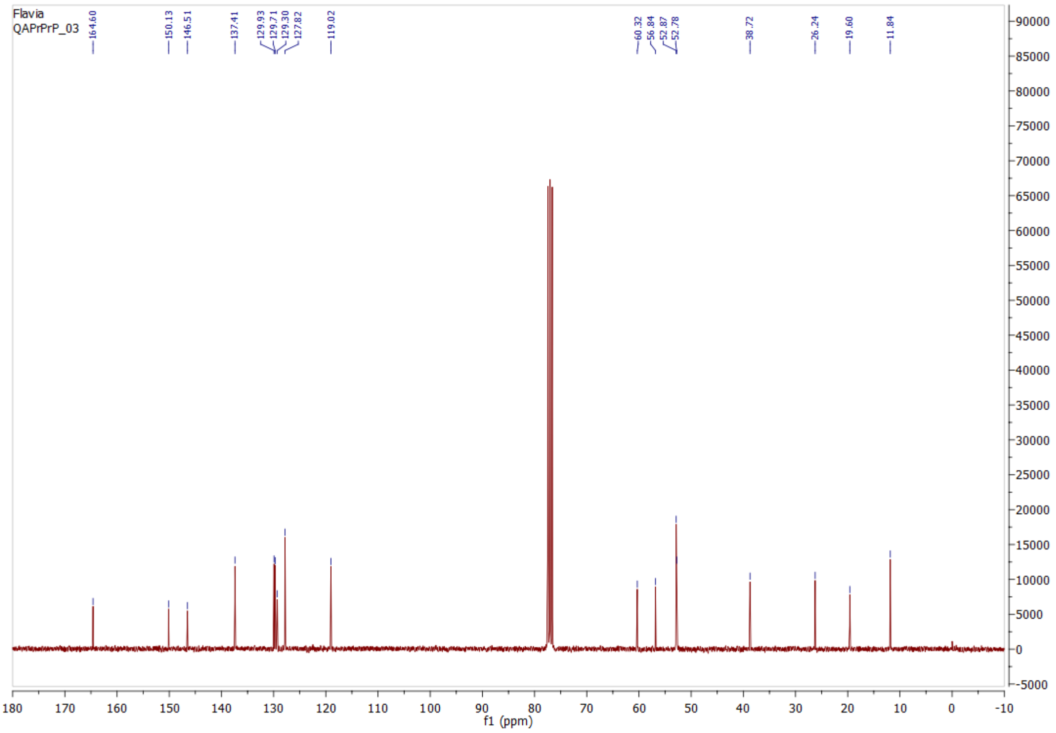

Figure S56. *N*-[3-(4-Propylpiperazin-1-yl)propyl]quinoline-2-carboxamide (**LINS05611**). ^{13}C NMR (75 MHz, $CDCl_3$) δ 164.6, 150.1, 146.5, 137.4, 129.9, 129.7, 129.3, 127.8, 127.8, 119.0, 60.3, 56.8, 52.9, 52.8, 38.7, 26.2, 19.6, 11.8.

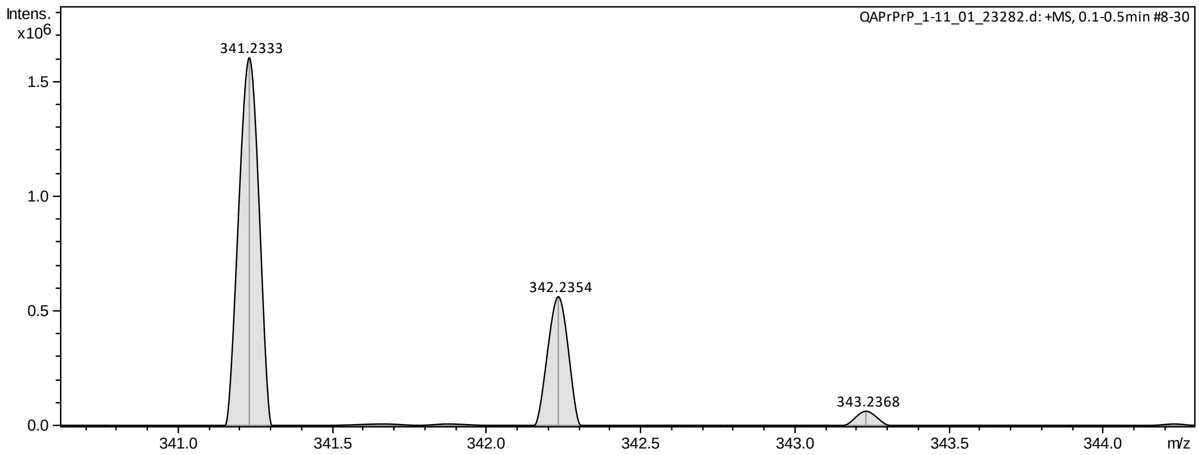

Figure S57. *N*-[3-(4-Propylpiperazin-1-yl)propyl]quinoline-2-carboxamide (**LINS05611**). HRMS (ESI) *m/z*: $[M+H]^+$ calcd.: 341.2335; $[M+H]^+$ found: 341.2333.

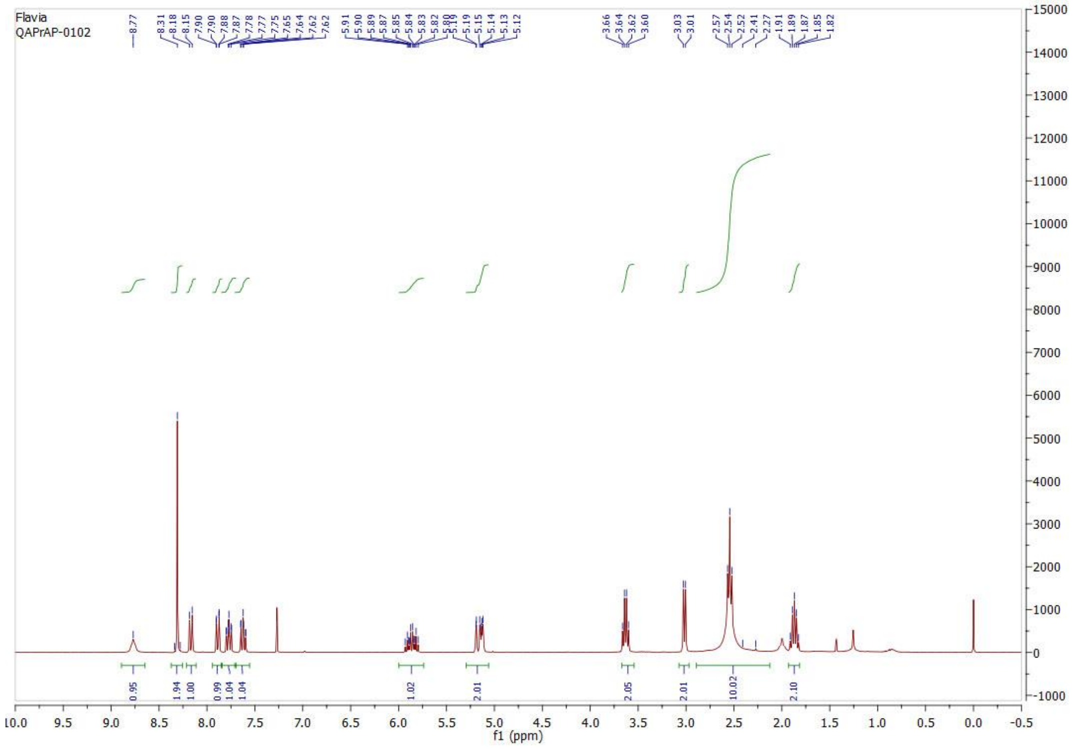

Figure S58. *N*-[3-(4-Allylpiperazin-1-yl)propyl]quinoline-2-carboxamide (**LINS05612**). ^{1}H NMR (300 MHz, $CDCl_3$) δ 8.77 (s, 1H), 8.36 – 8.26 (m, 2H), 8.17 (d, J = 8.4 Hz, 1H), 7.89 (dd, J = 8.2, 1.1 Hz, 1H), 7.77 (ddd, J = 8.4, 6.9, 1.1 Hz, 1H), 7.66 – 7.58 (m, 1H), 5.86 (ddt, J = 16.8, 10.1, 6.6 Hz, 1H), 5.25 – 5.06 (m, 2H), 3.63 (dd, J = 12.7, 6.5 Hz, 2H), 3.02 (d, J = 6.6 Hz, 2H), 2.69 – 1.94 (m, 10H), 1.87 (quint, J = 6.5 Hz, 2H).

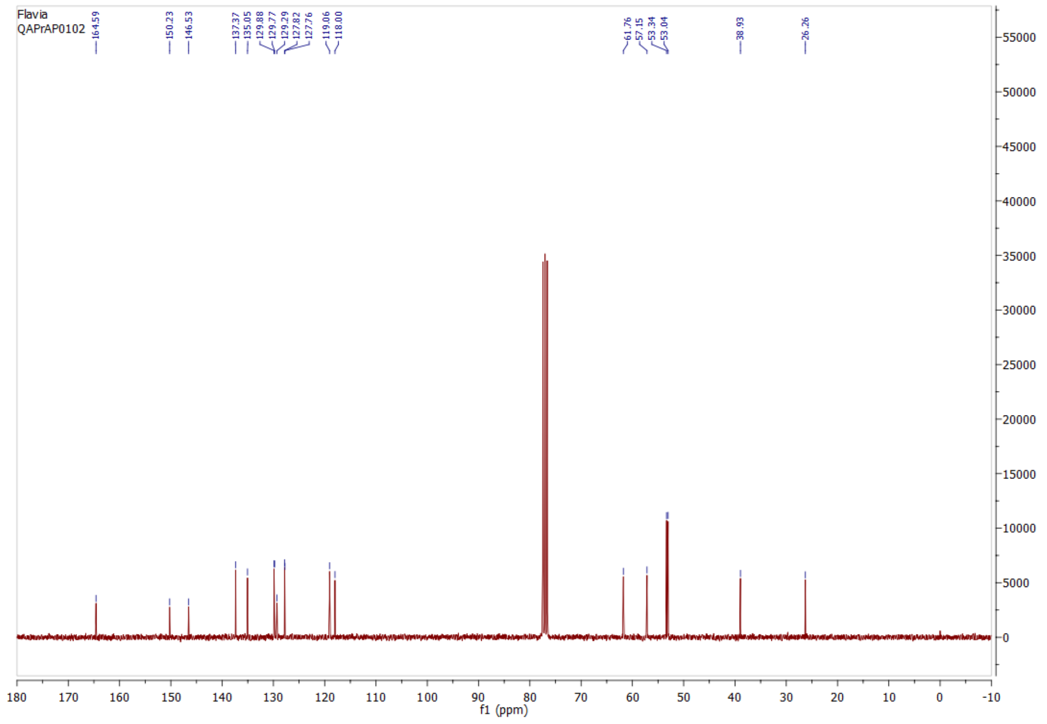

Figure S59. *N*-[3-(4-Allylpiperazin-1-yl)propyl]quinoline-2-carboxamide (**LINS05612**). ^{13}C NMR (75 MHz, $CDCl_3$) δ 164.6, 150.2, 146.5, 137.4, 135.1, 129.9, 129.8, 129.3, 127.8, 127.8, 119.1, 118.0, 61.8, 57.2, 53.3, 53.0, 38.9, 26.3.

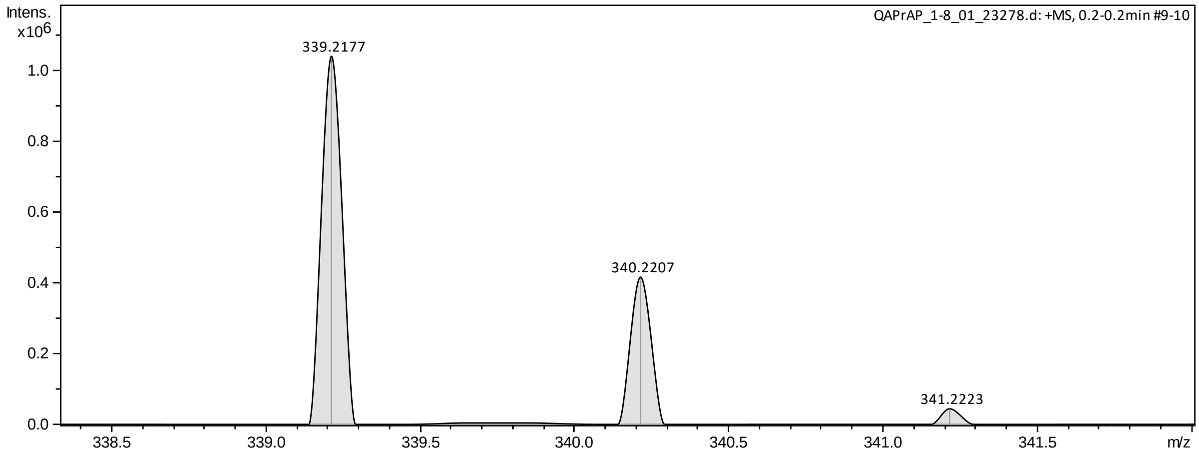

Figure S60. *N*-[3-(4-Allylpiperazin-1-yl)propyl]quinoline-2-carboxamide (**LINS05612**). HRMS (ESI) *m/z*: $[M+H]^+$ calcd.: 339.2179; $[M+H]^+$ found: 339.2177.

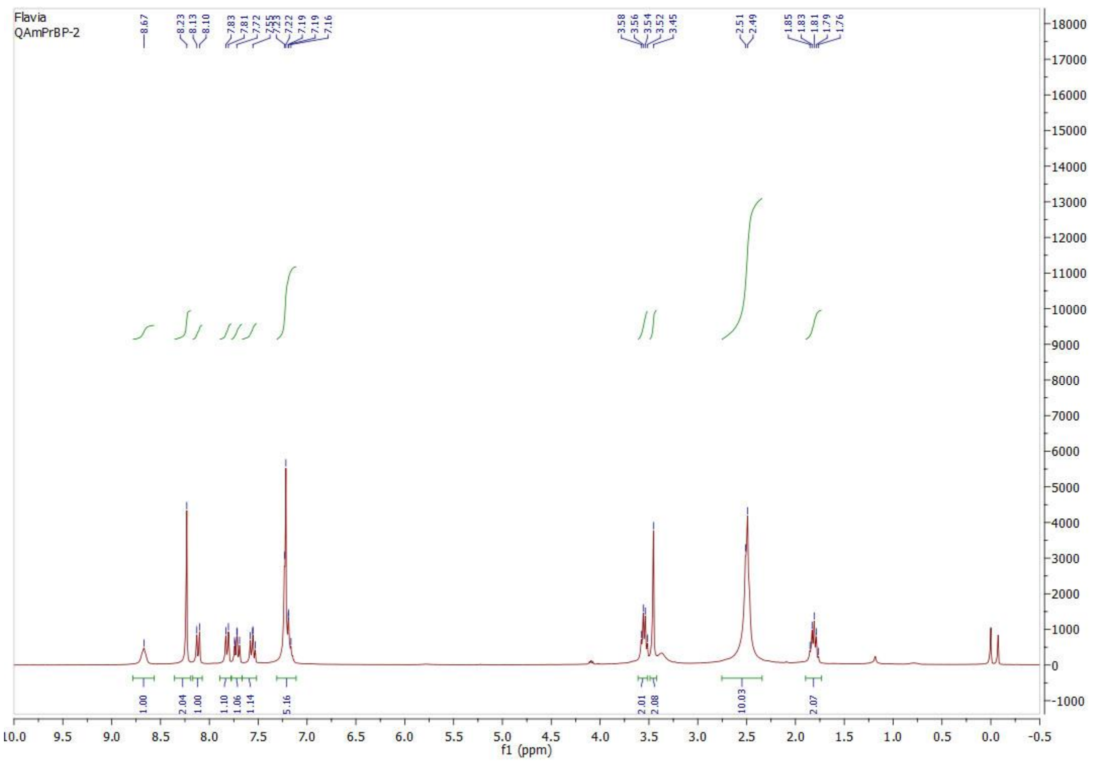

Figure S61. *N*-[3-(4-Benzylpiperazin-1-yl)propyl]quinoline-2-carboxamide (**LINS05613**). ^{1}H NMR (300 MHz, $CDCl_3$) δ 8.67 (s, 1H), 8.23 (s, 2H), 8.11 (d, J = 8.3 Hz, 1H), 7.82 (d, J = 8.3 Hz, 1H), 7.76 – 7.67 (m, 1H), 7.61 – 7.51 (m, 1H), 7.37 – 7.10 (m, 5H), 3.55 (dd, J = 11.8, 6.1 Hz, 2H), 3.45 (s, 2H), 2.69 – 2.35 (m, 10H), 1.89 – 1.74 (m, 2H).

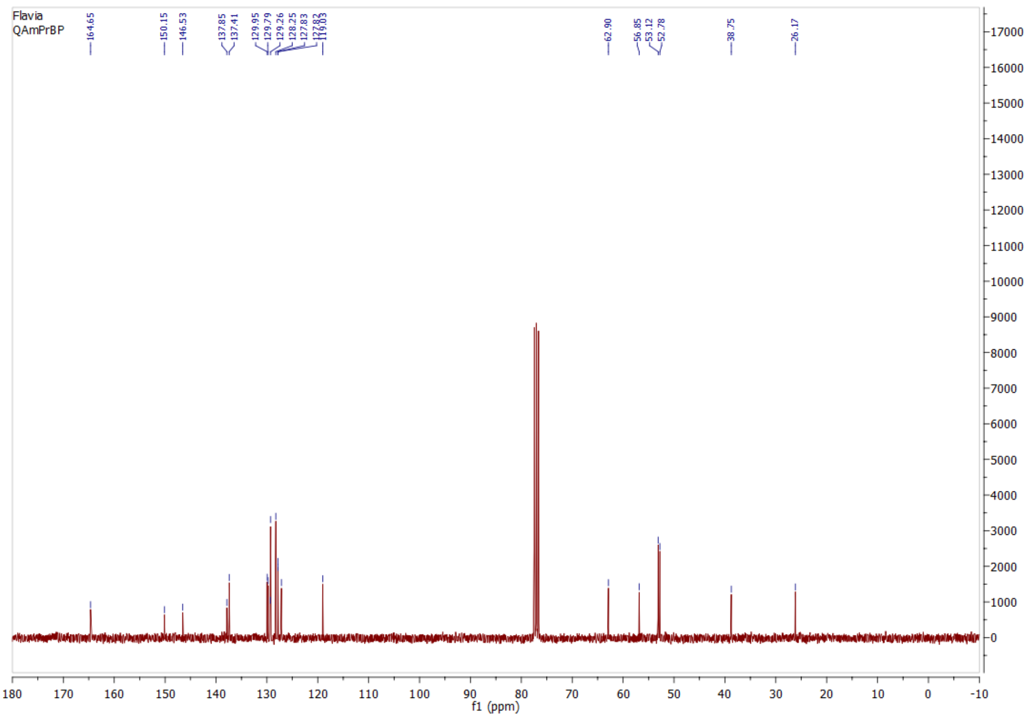

Figure S62. *N*-[3-(4-Benzylpiperazin-1-yl)propyl]quinoline-2-carboxamide (**LINS05613**). ^{13}C NMR (75 MHz, $CDCl_3$) δ 164.6, 150.2, 146.5, 137.8, 137.4, 129.9, 129.8, 129.3, 129.3, 128.2, 127.8, 127.8, 127.1, 119.0, 62.9, 56.8, 53.1, 52.8, 38.8, 26.2.

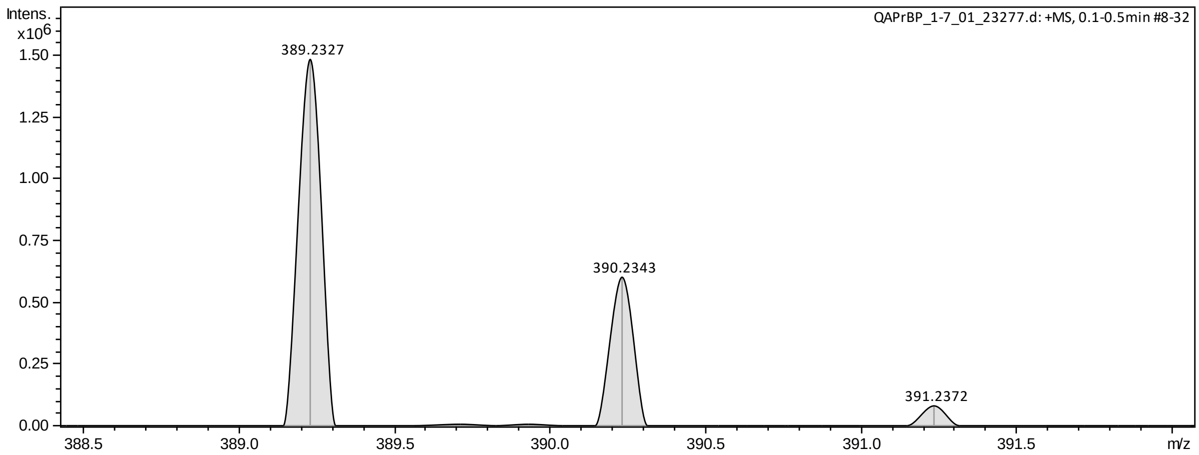

Figure S63. *N*-[3-(4-Benzylpiperazin-1-yl)propyl]quinoline-2-carboxamide (**LINS05613**). HRMS (ESI) *m/z*: $[M+H]^+$ calcd.: 389.2335; $[M+H]^+$ found: 389.2327.

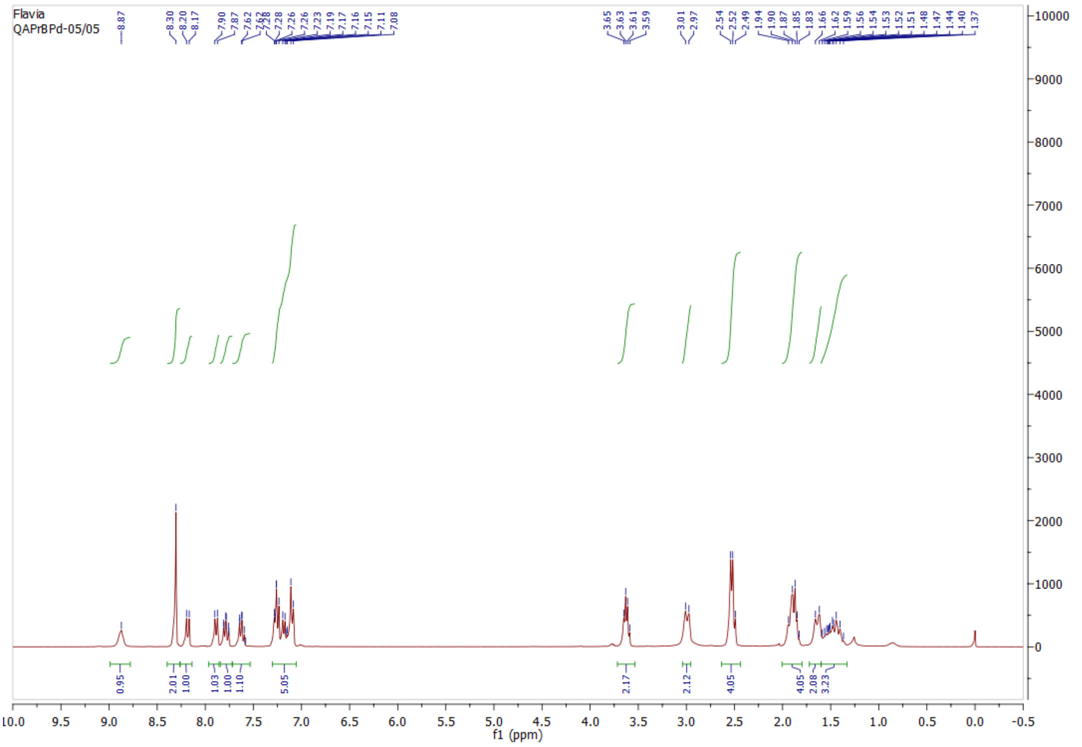

Figure S64. *N*-[3-(4-Benzyl-1-piperidyl)propyl]quinoline-2-carboxamide (**LINS05616**). ^{1}H NMR (300 MHz, $CDCl_3$) δ 8.87 (sl, 1H), 8.30 (s, 2H), 8.18 (d, J = 8.4 Hz, 1H), 7.89 (d, J = 8.1 Hz, 1H), 7.78 (dt, J = 8.1, 1.5 Hz, 1H), 7.71 – 7.58 (m, 1H), 7.33 – 7.06 (m, 5H), 3.72 – 3.55 (m, 2H), 2.99 (d, J = 10.6 Hz, 2H), 2.63 – 2.44 (m, 4H), 2.00 – 1.79 (m, 4H), 1.64 (d, J = 12.2 Hz, 2H), 1.59 – 1.31 (m, 3H).

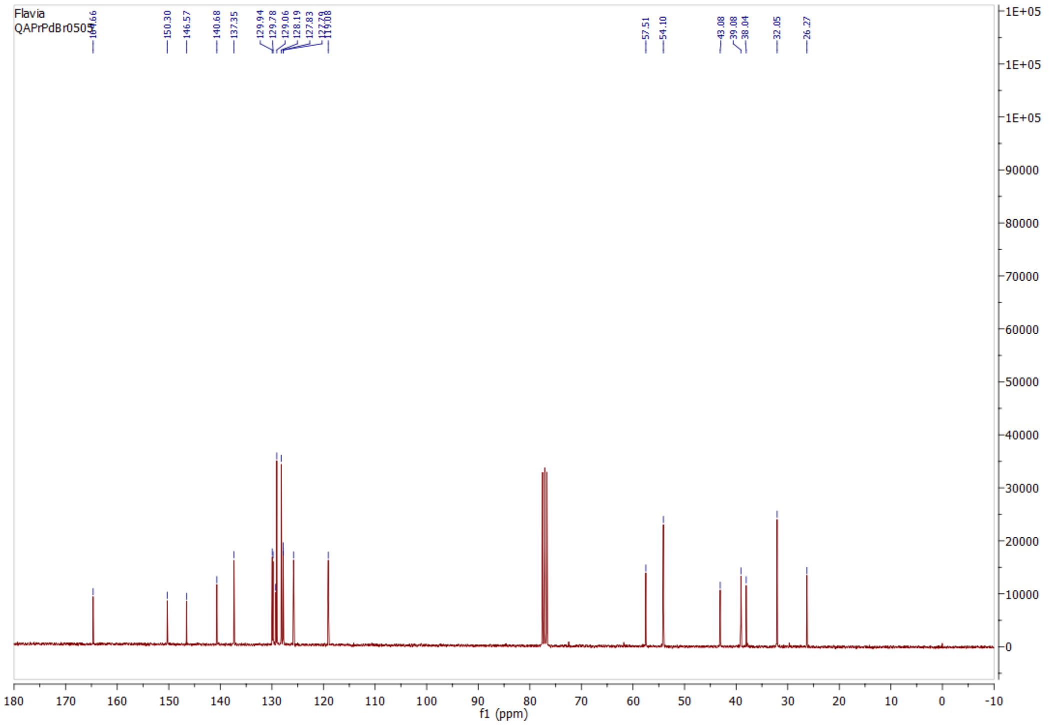

Figure S65. *N*-[3-(4-Benzyl-1-piperidyl)propyl]quinoline-2-carboxamide (**LINS05616**). ^{13}C NMR (75 MHz, $CDCl_3$) δ 164.7, 150.3, 146.6, 140.7, 137.3, 129.9, 129.8, 129.3, 129.1, 128.2, 127.8, 127.8, 125.8, 119.1, 57.5, 54.1, 43.1, 39.1, 38.0, 32.0, 26.3.

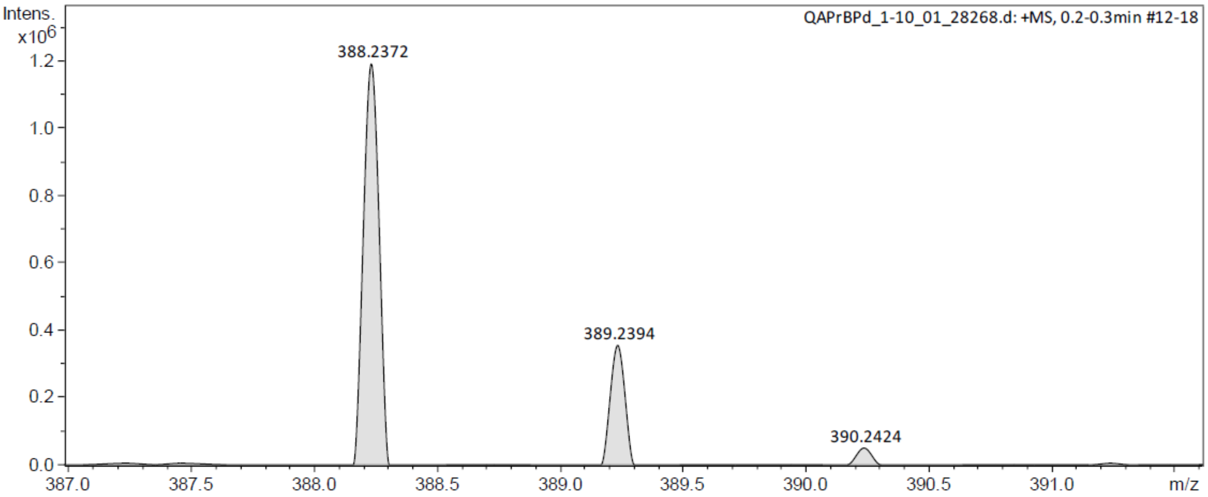

Figure S66. *N*-[3-(4-Benzyl-1-piperidyl)propyl]quinoline-2-carboxamide (**LINS05616**). HRMS (ESI) *m/z*: $[M+H]^+$ calcd.: 388.2383; $[M+H]^+$ found: 388.2372.

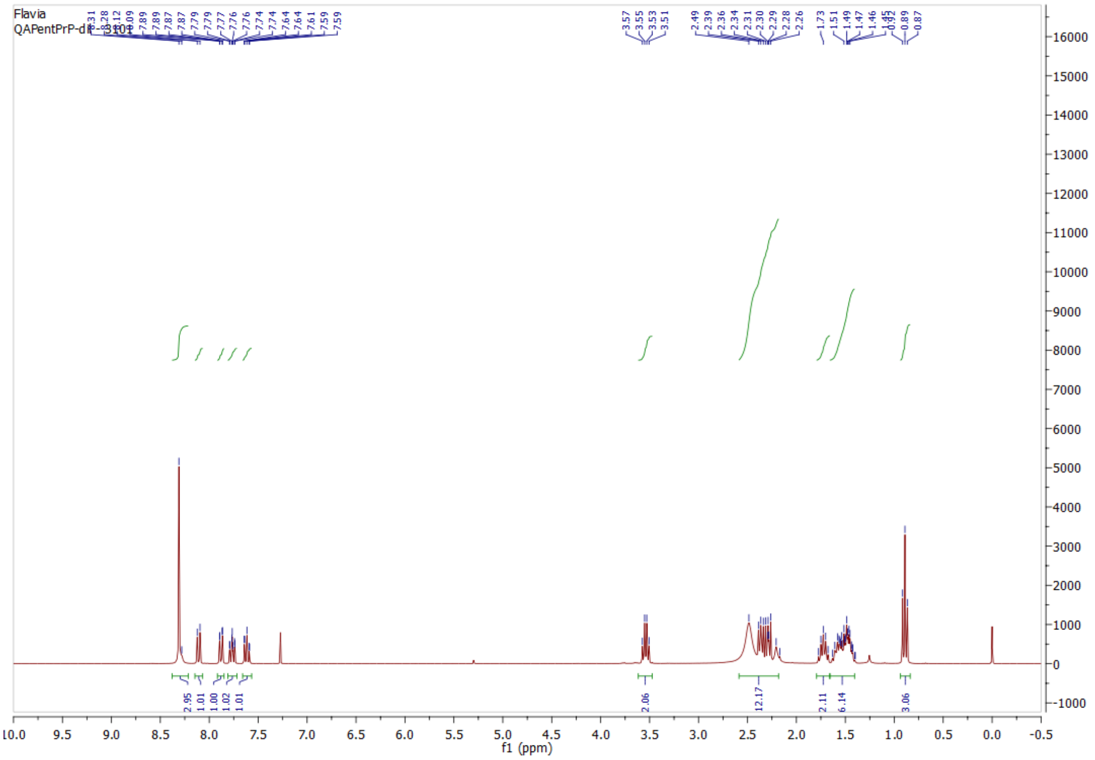

Figure S67. *N*-[5-(4-Propylpiperazin-1-yl)pentyl]quinoline-2-carboxamide (**LINS05631**). ^{1}H NMR (300 MHz, $CDCl_3$) δ 8.40 – 8.21 (m, 3H), 8.11 (d, J = 8.5 Hz, 1H), 7.88 (dd, J = 8.2, 0.8 Hz, 1H), 7.80 – 7.72 (m, 1H), 7.65 – 7.58 (m, 1H), 3.54 (dd, J = 12.4, 6.8 Hz, 2H), 2.58 – 2.17 (m, 12H), 1.78 – 1.67 (m, 2H), 1.64 – 1.40 (m, 6H), 0.89 (t, J = 7.4 Hz, 3H).

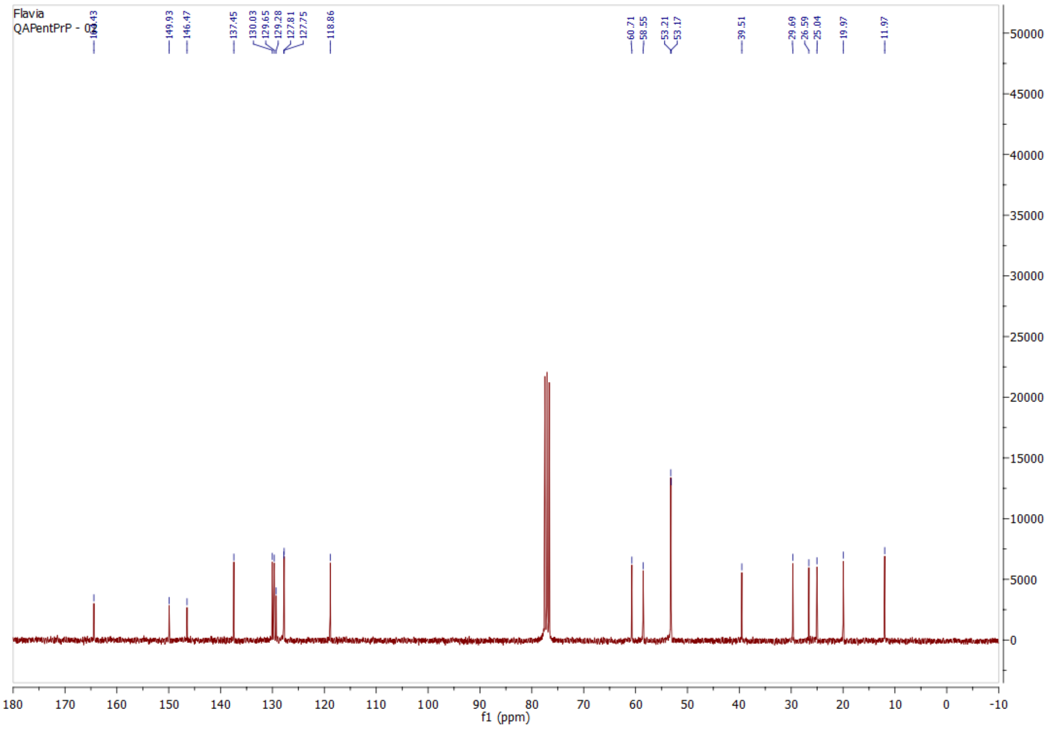

Figure S68. *N*-[5-(4-Propylpiperazin-1-yl)pentyl]quinoline-2-carboxamide (**LINS05631**). ^{13}C NMR (75 MHz, $CDCl_3$) δ 164.4, 149.9, 146.5, 137.4, 130.0, 129.6, 129.3, 127.8, 127.8, 118.9, 60.7, 58.5, 53.2, 53.2, 39.5, 29.7, 26.6, 25.0, 19.9, 11.9.

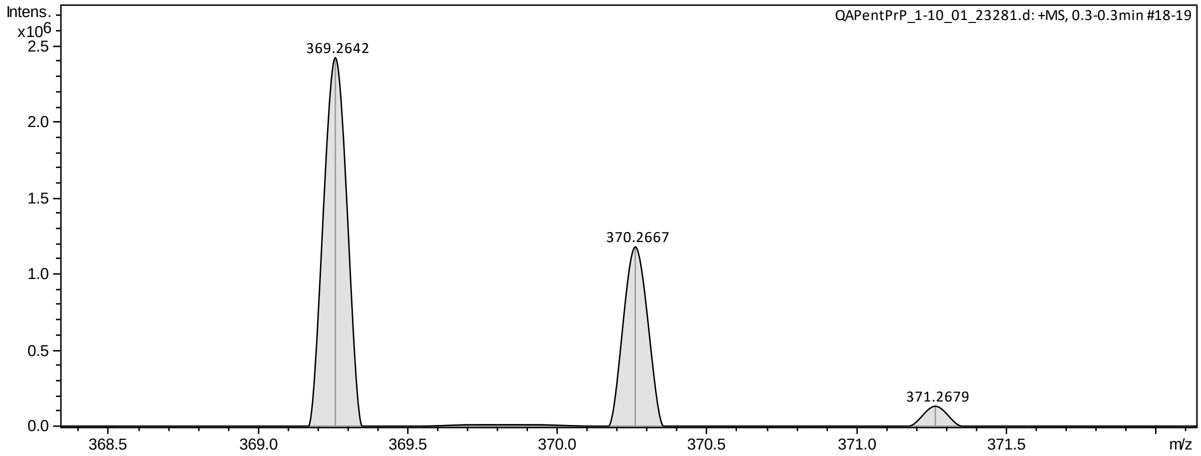

Figure S69. *N*-[5-(4-Propylpiperazin-1-yl)pentyl]quinoline-2-carboxamide (**LINS05631**). HRMS (ESI) *m/z*: $[M+H]^+$ calcd.: 369.2648; $[M+H]^+$ found: 369.2642.

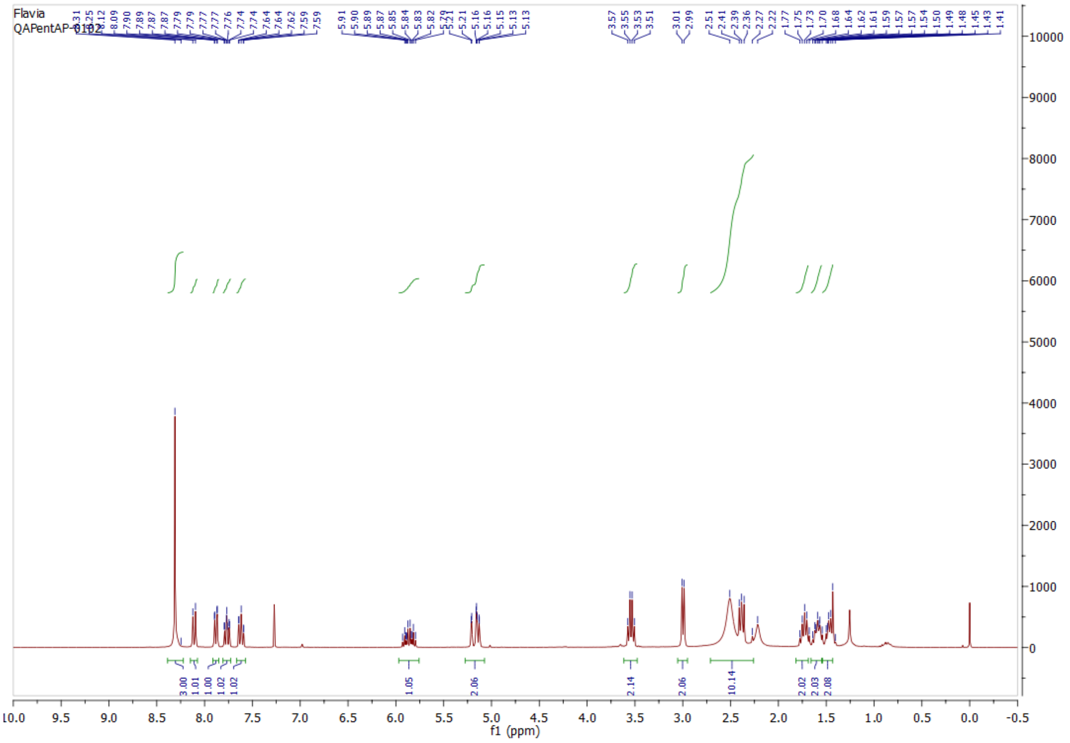

Figure S70. *N*-[5-(4-Allylpiperazin-1-yl)pentyl]quinoline-2-carboxamide (**LINS05632**). ^{1}H NMR (300 MHz, $CDCl_3$) δ 8.42 – 8.21 (m, 3H), 8.11 (d, J = 8.4 Hz, 1H), 7.88 (dd, J = 8.2, 0.8 Hz, 1H), 7.77 (ddd, J = 8.4, 6.9, 0.8 Hz, 1H), 7.67 – 7.56 (m, 1H), 5.86 (ddt, J = 16.8, 10.1, 6.6 Hz, 1H), 5.24 – 5.08 (m, 2H), 3.54 (dd, J = 13.5, 6.9 Hz, 2H), 3.00 (d, J = 6.6 Hz, 2H), 2.64 – 2.15 (m, 10H), 1.82 – 1.66 (m, 2H), 1.65 – 1.51 (m, 2H), 1.51 – 1.38 (m, 2H).

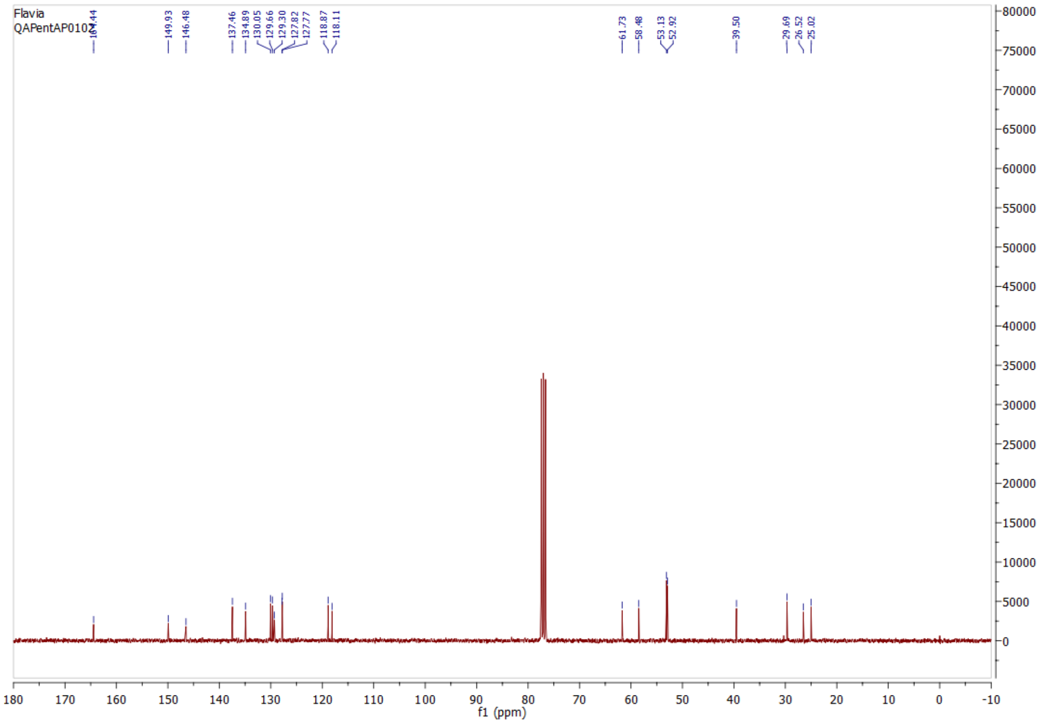

Figure S71. *N*-[5-(4-Allylpiperazin-1-yl)pentyl]quinoline-2-carboxamide (**LINS05632**). ^{13}C NMR (75 MHz, $CDCl_3$) δ 164.4, 149.9, 146.5, 137.5, 134.9, 130.1, 129.7, 129.3, 127.8, 127.8, 118.9, 118.1, 61.7, 58.5, 53.1, 52.9, 39.5, 29.7, 26.5, 25.0.

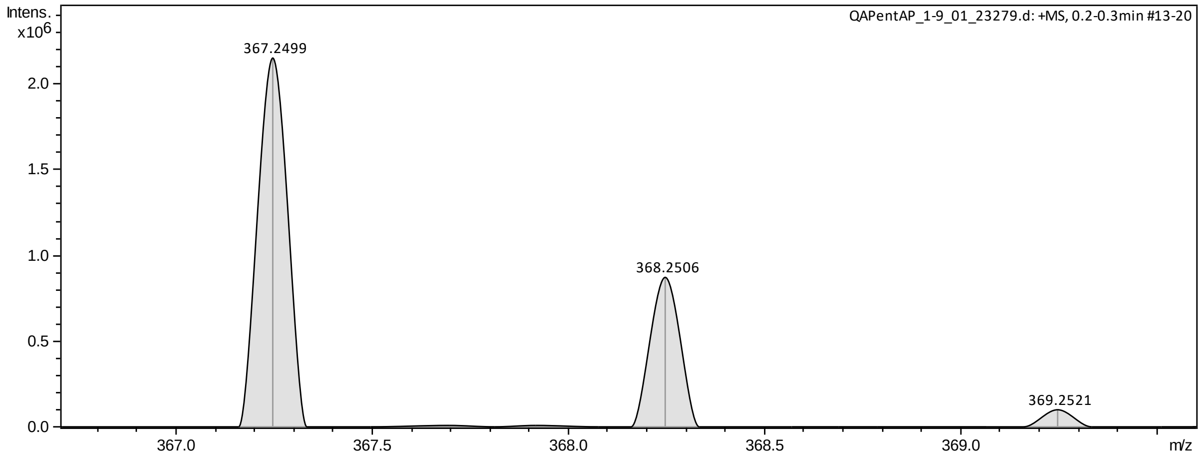

Figure S72. *N*-[5-(4-Allylpiperazin-1-yl)pentyl]quinoline-2-carboxamide (**LINS05632**). HRMS (ESI) *m/z*: $[M+H]^+$ calcd.: 367.2492; $[M+H]^+$ found: 367.2499.

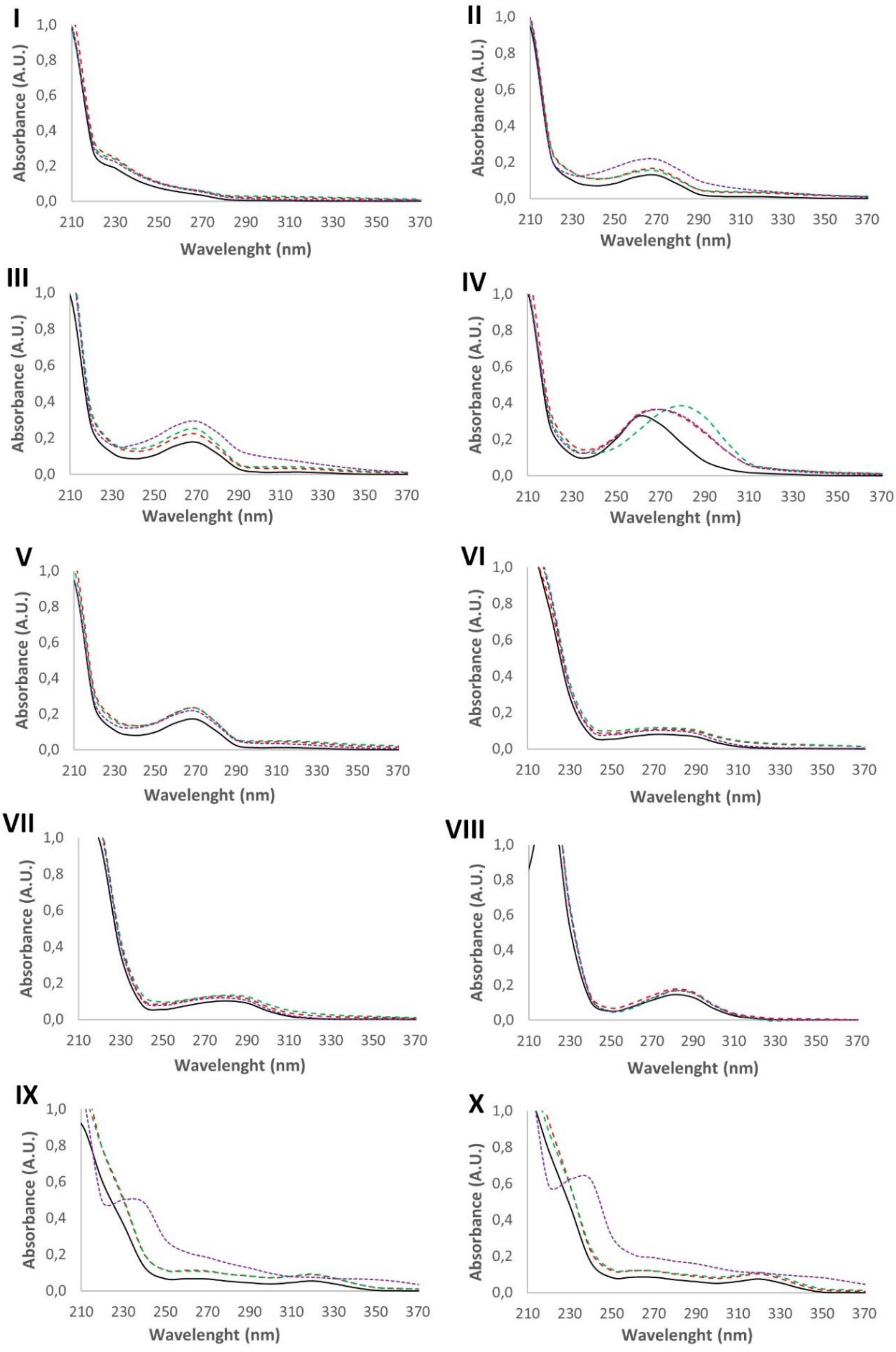

Figure S73. UV Absorption spectra of compounds **113** (I), **210** (II), **213** (III), **214** (IV), **215** (V), **310** (VI), **313** (VII), **316** (VIII) **410** (IX), **412** (X) alone (50 µM, black lines) or in the presence of Fe^{2+} (red dashed lines), Fe^{3+} (green dashed lines) or Cu^{2+} (purple dashed lines) ions in equimolar concentrations.
